# Insights from basic adjunctive examinations of GCK‐MODY, HNF1A‐MODY, and type 2 diabetes: A systemic review and meta‐analysis

**DOI:** 10.1111/1753-0407.13390

**Published:** 2023-05-24

**Authors:** Jing Liu, Xinhua Xiao, Qian Zhang, Miao Yu

**Affiliations:** ^1^ Key Laboratory of Endocrinology of National Health Commission, Diabetes Research Center of Chinese Academy of Medical Sciences, Department of Endocrinology, Translational Medicine Center, Peking Union Medical College Hospital Chinese Academy of Medical Sciences and Peking Union Medical College Beijing People's Republic of China

**Keywords:** clinical laboratory techniques, diabetes mellitus, type 2, maturity‐onset diabetes of the young, type 2, maturity‐onset diabetes of the young, type 3, 临床实验室技术, 青少年发病的成人糖尿病2型, 青少年发病的成人糖尿病3型, 2型糖尿病

## Abstract

**Background:**

Glucokinase maturity‐onset diabetes of the young (GCK‐MODY) is difficult to distinguish from other diabetic forms. This article aims to characterize the differences in results from routine examinations between GCK‐MODY and hepatocyte nuclear factor 1‐α (HNF1A)‐MODY or type 2 diabetes (T2D) patients in different periods of diabetes.

**Methods:**

Ovid Medline, Embase, and the Cochrane Library were searched up until October 9, 2022 for articles containing baseline characteristics of GCK‐MODY, HNF1A‐MOFY, and T2D, excluding pregnant women. The pooled standardized mean differences were derived using a random‐effects model.

**Results:**

Compared to HNF1A‐MODY, GCK‐MODY patients had lower indicators of glucose metabolism. Total triglycerides (TG) (−0.93 [−1.66, −0.21] mmol/l) were consistently lower in GCK‐MODY patients in the all‐family‐members subgroup analysis. Compared to T2D, GCK‐MODY patients were younger at diagnosis and had lower body mass index (BMI), lower high‐sensitivity C‐reactive protein (hsCRP) (−0.60 [−0.75, −0.44] mg/l), lower fasting C‐peptide (FCP), and lower 2‐hour postprandial glucose (2‐h PG). Indicators of glycated hemoglobin (HbA1c) and fasting blood glucose (FPG) were consistently lower in subgroup studies with all family members of GCK‐MODY patients as well.

**Conclusions:**

Lower HbA1c, FPG, 2‐h PG, and change in 2‐h PG may help to diagnose GCK‐MODY differentially from HNF1A‐MODY at an early stage, and lower TG may strengthen such a diagnosis in the follow‐up stages. Younger age combined with lower BMI, FCP, hsCRP, and 2‐h PG may be useful to distinguish GCK‐MODY from MODY‐like T2D, whereas results of glucose metabolism indicators such as HbA1c and FPG may not help physicians until after a long follow‐up period.

## Graphical abstract

**Highlights**

- For distinguishing glucokinase maturity‐onset diabetes of the young (GCK‐MODY) from hepatocyte nuclear factor 1‐α MODY (HNF1A‐MODY), total triglycerides may help during the follow‐up period. In order to discriminate GCK‐MODY from MODY‐like type 2 diabetes, high‐sensitivity C‐reactive protein may be a suitable indicator in the early stage, whereas HbA1c and fasting blood glucose may not help until the follow‐up stage. The role of the aforementioned four indicators for identifying GCK‐MODY in the shorter or longer duration from corresponding diabetic forms in the general clinical setting should be reassessed.

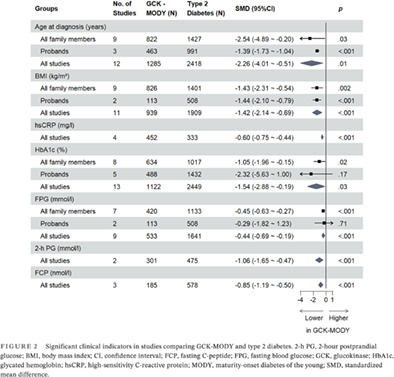

## 摘要

**【摘要】 背景** 青少年起病的成人型糖尿病(MODY)2 型(GCK‐MODY)很难与其他类型的糖尿病区分。本文旨在探讨GCK‐MODY与肝细胞核因子1α(HNF1A)‐MODY或2型糖尿病患者在糖尿病不同阶段的常规检查结果的差异。**方法** 通过Ovid Medline、Embase和Cochrane图书馆检索截至2022年10月9日的文献，收集包含GCK‐MODY、HNF1A‐MODY和2型糖尿病基线特征的研究，排除孕妇。采用随机效应模型计算汇总标准化均数差。**结果** 与HNF1A‐MODY相比，GCK‐MODY患者的葡萄糖代谢指标较低。在全家庭成员亚组分析中，GCK‐MODY患者的总甘油三酯(TG)[‐0.93(‐1.66，‐0.21)mmol/L]始终较低。与2型糖尿病患者相比，GCK‐MODY患者的诊断年龄较小，体质指数(BMI)较低，高敏C‐反应蛋白(hsCRP)较低[‐0.60(‐0.75，‐0.44)mg/L]，空腹C肽(FCP)较低，餐后2小时血糖(2h PG)较低。HbA_1C_和空腹血糖(FPG)指标在包括GCK‐MODY患者所有家族成员的亚组研究中也一致较低。**结论** 较低的HbA_1C_、FPG、2hPG和2hPG的变化可帮助在早期阶段区分GCK‐MODY与HNF1A‐MODY，而较低的TG在随访阶段可增强此类诊断。较年轻的年龄结合较低的BMI、FCP、hsCRP和2hPG可能有助于区分GCK‐MODY和类MODY的2型糖尿病，而HbA_1C_和FPG等糖尿病代谢指标的结果可能需要长期随访后才能对医生有所帮助。

## 1 INTRODUCTION

Diabetes is a chronic disease that affects approximately 537 million people (2021) and is predicted to affect 106 million more by 2030.^1^ As health practitioners have tried to mitigate the incidence of the disease, their efforts have been hampered by the fact that accurate diagnosis is difficult due to the many different subtypes of diabetes. One disease that is particularly troublesome to identify is maturity‐onset diabetes of the young (MODY), which is a monogenic diabetic disease whose most prevalent types are glucokinase (GCK)‐MODY, hepatocyte nuclear factor 1‐α (HNF1A)‐MODY, and hepatocyte nuclear factor 4‐α MODY. GCK‐MODY is caused by glucokinase mutation and typically results in mild hyperglycemia with a low prevalence of diabetic microvascular and macrovascular complications.^2^, ^3^ Patients with GCK‐MODY generally may not need to take hypoglycemic agents, except in some circumstances during pregnancy.^4^ By contrast, HNF1A‐MODY involves obvious hyperglycemia and a high predisposition to diabetic vascular complications,^2^, ^5^ but it responds well to sulfonylurea hypoglycemic agents.^4^

Genetic testing is the best way to confirm genetic diabetes, but this technology is relatively expensive for patients and therefore past studies have been conducted for the purpose of exploring if certain clinical indicators can effectively help distinguish between GCK‐MODY, HNF1A‐MODY, and type 2 diabetes that should be further tested for MODY. For example, high‐sensitivity C‐reactive protein (hsCRP),^6^ 1,5‐anhydroglucitol,^7^ and plasma‐fucosylated glycans^8^ are evidently lower in HNF1A‐MODY patients. There are also some prediction tools or indices for physicians to use in differentiating between MODY and type 1 diabetes or type 2 diabetes.^7^, ^9^ However, most studies have been regional and restricted to the low population of MODY patients because of its low incidence compared to type 1 diabetes and type 2 diabetes. Furthermore, indicators such as 1,5‐anhydroglucitol and plasma‐fucosylated glycans are rarely tested in routine clinical settings, making it difficult to use these indicators to distinguish between different diabetes subtypes. Additionally, the specific differences in basic clinical results, especially indicators for lipid metabolism, between GCK‐MODY, HNF1A‐MODY, and type 2 diabetes, as well as the difference between the proband group and the all‐family‐members group in MODY patients, have yet to be comprehensively characterized. Hence, we conducted this systematic review and meta‐analysis in order to compare the worldwide trends in basic clinical diabetes results so that we may be able to provide a reference for more accurate diagnosis with common indices in the future, which will facilitate the differential diagnosis of GCK‐MODY patients and their ensuing medical treatments. At the same time, this study will allow for more accurate identification awaiting the later genetic testing confirmation, thus offering a more cost‐effective strategy for patients.

## 2 METHODS

This meta‐analysis was conducted and reported in accordance with the 2020 version of the Preferred Reporting Items for Systematic Reviews and Meta‐Analyses Guidelines (PRISMA) (Supplementary Table S1 in Appendix S1)^10^ and Meta‐analysis of Observational Studies in Epidemiology (MOOSE) reporting guidelines (Supplementary Table S2 in Appendix S1).^11^ The study protocol was registered on Research Registry (reviewregistry1456). No human or animal subjects were involved and no ethical statement for this study.

### 2.1 Objectives

The primary objective of this study was to compare the baseline clinical characteristics of GCK‐MODY, HNF1A‐MODY, and type 2 diabetes. The PICO (population, intervention, comparison, outcome) setting of the meta‐analysis was as follows: (1) P: patients with GCK‐MODY; (2) I: the confirmation of GCK‐MODY or HNF1A‐MODY requiring genetic testing; (3) C: the control group had to contain HNF1A‐MODY or type 2 diabetes patients; and (4) O: all of the studies had to contain necessary basic baseline information and clinical examinations. Our primary outcome was the existence of differences between patients with GCK‐MODY and those with HNF1A‐MODY or type 2 diabetes in any kind of characteristic measured at baseline.

### 2.2 Search strategy and eligibility criteria

In order to include articles involving basic clinical examination results in populations with GCK‐MODY, HNF1A‐MODY, and type 2 diabetes, we searched Ovid Medline and Embase up until October 9, 2022 using the MeSH and Emtree keywords containing “Maturity‐onset diabetes of the young 2,” “biomarker*” OR “diagno*” OR “screen*” OR “profile*” OR “predict*” OR “algorithm, *” including these words' variations and synonyms (Supplementary Table S3 in Appendix S1). We ruled out articles without human subjects and those that included pregnant women. To retrieve all eligible studies, we set no limitations on included article types, nor were any language restrictions set.

The Cochrane Library was searched to assess gray literature and unpublished studies, and the latest articles published from randomized controlled trials protocols were also retrieved to avoid omitting any eligible studies. With the aim of choosing populations with better representativeness for GCK‐MODY and HNF1A‐MODY, we included only studies that diagnosed patients using genetic testing. The number of patients with GCK‐MODY or type 2 diabetes/HNF1A‐MODY in studies had to be greater than 10, and the other group had to contain >5 patients. For multiple papers that relied on previously published data, we selected the one with more outcomes reported or with more appropriate criteria in the comparison group.

Studies with the following characteristics were excluded: (1) studies containing pregnant diabetic patients; (2) studies not containing the target population or outcome; (3) studies not containing enough patients; and (4) studies for which the full text was not available.

### 2.3 Data extraction

The overall screening and selection strategy included two phases. The first stage screened papers by the title and abstract, and the second was full‐text screening and selection. One author (Liu) assessed the eligibility of all studies and extracted data in accordance with a prespecified data form, and another author (Xiao) quality‐checked all extracted data. Data extracted included variables needed for this study's objective analysis: author, publication year, study design, the sample size of the two patient groups, age at clinical examination, duration of diabetes, positive family history and its principles, positive pancreatic auto‐antibodies, baseline data (age at diagnosis of diabetes, body mass index [BMI], birthweight, hsCRP), glucose metabolism (glycated hemoglobin [HbA1c]; blood glucose at fasting (FPG), 1‐hour postprandial time and 2‐hour postprandial time (2‐h PG); change in 2‐h PG (Δ2‐h PG, the reference baseline was fasting blood glucose of the individual patient); C‐peptide and insulin at fasting and 2‐hour postprandial time); lipid metabolism (total cholesterol [TC], total triglycerides [TG], high‐density lipoprotein [HDL], and low‐density lipoprotein [LDL]) and treatment protocols. As HNF1A‐MODY and type 2 diabetes are two distinctly different diseases, we compared them to GCK‐MODY separately. In order to obtain more eligible data from articles, if a study group provided data comparing GCK‐MODY with HNF1A‐MODY and type 2 diabetes in separate articles or the same article, we extracted separate information for each disease from corresponding articles. Because the study of Campos et al^12^ provided separate clinical data from probands and relatives, we extracted the data of these two groups from this article as two separate data sources to analyze and categorized the subgroup with relatives into the subgroup with all family members when meta‐analyzing the data. Because all studies involved patients with hyperglycemia who had been examined prior to genetic confirmation, we considered all of them to be case–control studies.

### 2.4 Bias assessment and sensitivity analysis

Bias assessment was conducted by one author (Liu) according to the Newcastle‐Ottawa criteria (Supplementary Table S5 in Appendix S1),^13^ and another author (Xiao) quality‐checked all evaluative data. Contour‐enhanced funnel plots with Egger's regression (*p* < .10)^14^ were used to evaluate potentially small study effects and publication bias in characteristics assessed in 10 or more studies, and the common‐random model was applied using the trim‐and‐fill method.^15^ Furthermore, for characteristics assessed in five or more studies, a subgroup analysis was conducted that focused on studies with patients from different sources. The Grading of Recommendations Assessment, Development and Evaluation (GRADE) instrument^16^ was used to evaluate the quality of evidence in indices assessed in two or more studies.

### 2.5 Statistical analysis

Quantitative data were expressed as mean ± SD using SPSS 26.0. For studies that reported the minimum, median, interquartile range, and maximum of the clinical results, the transformation to mean was conducted using Luo's method. Wan's method was used to calculate the SD when all four of the indicators were not given, and Shi's method was used when they were.^17^ The SD was calculated by multiplying the standard error by the square root of the number of patients, if applicable. To obtain the pooled effect size, a restricted maximum likelihood estimator random‐effects meta‐analysis model was used from a meta‐package in R statistical software (version 4.1.3). Plots in the article were also done with the meta package, or with the forestploter package. The method of Hedges' was used to estimate the standardized mean difference (SMD),^18^, ^19^ and the pooled SMD of each clinical result was reported with their 95% confidence interval (CI). Heterogeneity of studies was assessed by Q and I^2^ statistics, and the cutoff values of 50% for I^2^ and <.10 for *P* ^*h*^ (*p* for heterogeneity) values were taken to indicate the presence of heterogeneity. A two‐tailed test was applied in all comparisons, and a *p* value <.05 was considered to indicate statistically significant test results.

### 2.6 Data and resource availability

The data sets generated and analyzed during the current study are available from the corresponding author upon reasonable request.

## 3 RESULTS

A total of 3860 records were retrieved from our primary search (1186, 2526, and 148 records from Ovid Medline, Embase, and the Cochrane Library, respectively), yielding 544 eligible articles. Of these, 32 studies were finally included in the systematic review^2^, ^6^, ^12^, ^20^, ^21^, ^22^, ^23^, ^24^, ^25^, ^26^, ^27^, ^28^, ^29^, ^30^, ^31^, ^32^, ^33^, ^34^, ^35^, ^36^, ^37^, ^38^, ^39^, ^40^, ^41^, ^42^, ^43^, ^44^, ^45^, ^46^, ^47^, ^48^ (Supplementary Figure S1 in Appendix S1). There were 23 and 14 articles that compared GCK‐MODY with HNF1A‐MODY (HNF1A‐MODY studies) and type 2 diabetes (type 2 diabetes studies), respectively. The basic information of all included articles is provided in the Supplementary Table S4 in Appendix S1. For categories of patients, 7 studies (30.4%) limited their subjects to the pediatric group, 6 (26.1%) studies limited them to adult patients, and 10 studies (43.5%) set no limitation on the age of patients in the HNF1A‐MODY studies, and the corresponding numbers for type 2 diabetes studies were 6, 4, and 4, respectively. There were 13 articles (56.5%) that contained probands themselves, 5 articles involving all mutation‐carrying family members, 1 study that reported the clinical results of relatives of the probands, and 4 studies that did not specify a patient category in the HNF1A‐MODY studies. For type 2 diabetes studies, these numbers were five, eight, zero, and one, respectively. For the four studies^24^, ^26^, ^37^, ^47^ that did not specify whether they contained family members, we categorized them into the group that contained family members due to their wide range of age at recruitment and at diagnosis. For the study done by Barrio et al^41^ because they involved only pediatric probands and pediatric relatives, we classified this study into the proband group. Male patients accounted for 43.99%, 41.08%, and 44.82% in GCK‐MODY, HNF1A‐MODY, and type 2 diabetes, respectively. Type 2 diabetes studies were divided into seven (50%) that recruited young‐onset diabetes patients, two studies (14.3%) with well‐controlled type 2 diabetes patients, two studies (14.3%) with late‐onset type 2 diabetes patients, two studies (14.3%) without extra limitations on the types of type 2 diabetes patients, and one study (7.1%) with newly diagnosed type 2 diabetes patients.

**FIGURE 1:**
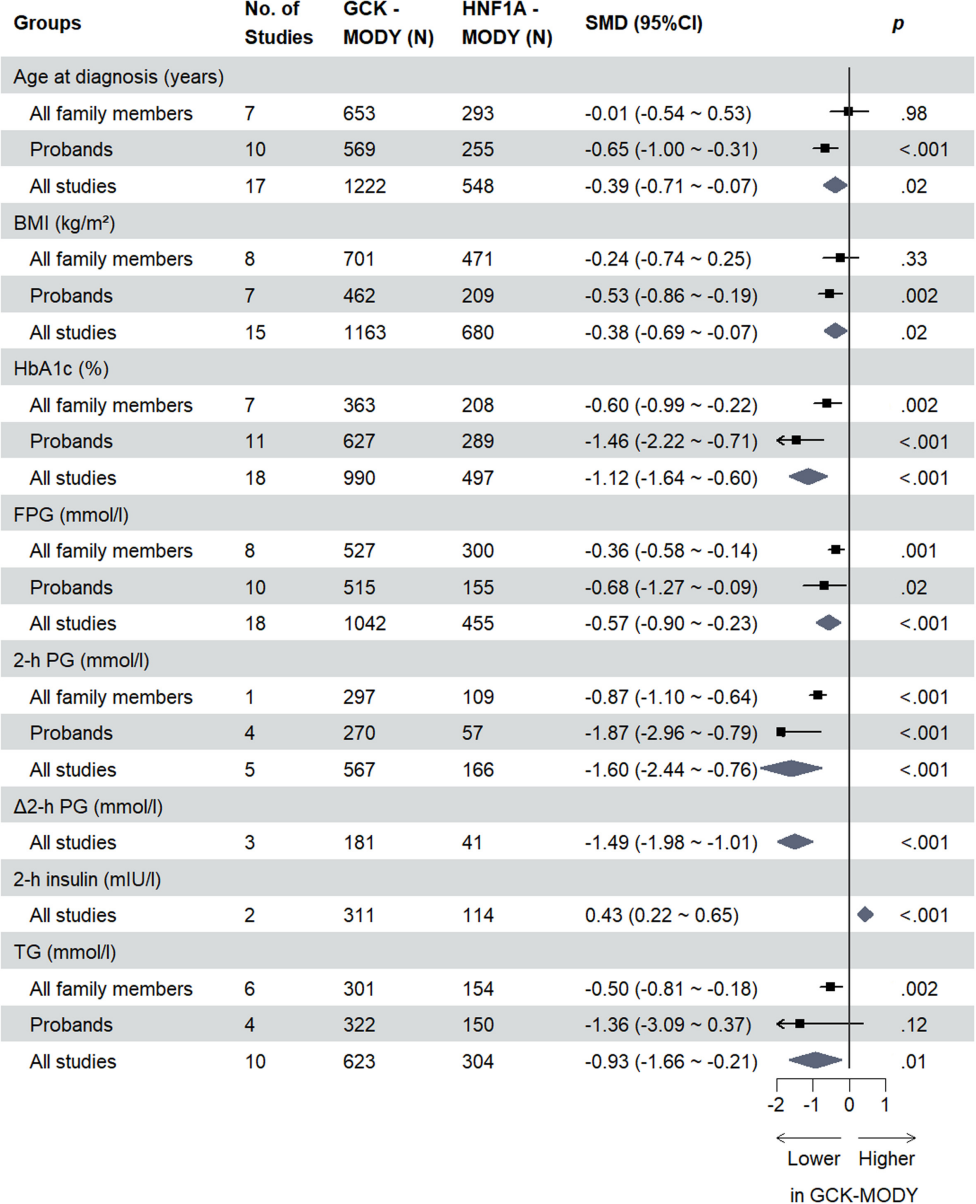
Significant clinical indicators in studies comparing GCK‐MODY and HNF1A‐MODY. 2‐h PG, 2‐hour postprandial glucose; Δ2‐h PG, change in 2‐h PG; 2‐h insulin, 2‐hour insulin; BMI, body mass index; CI, confidence interval; FPG, fasting blood glucose; GCK, glucokinase; HbA1c, glycated hemoglobin; HNF1A, hepatocyte nuclear factor 1‐α; MODY, maturity‐onset diabetes of the young; SMD, standardized mean difference; TG, total triglycerides.

**FIGURE 2:**
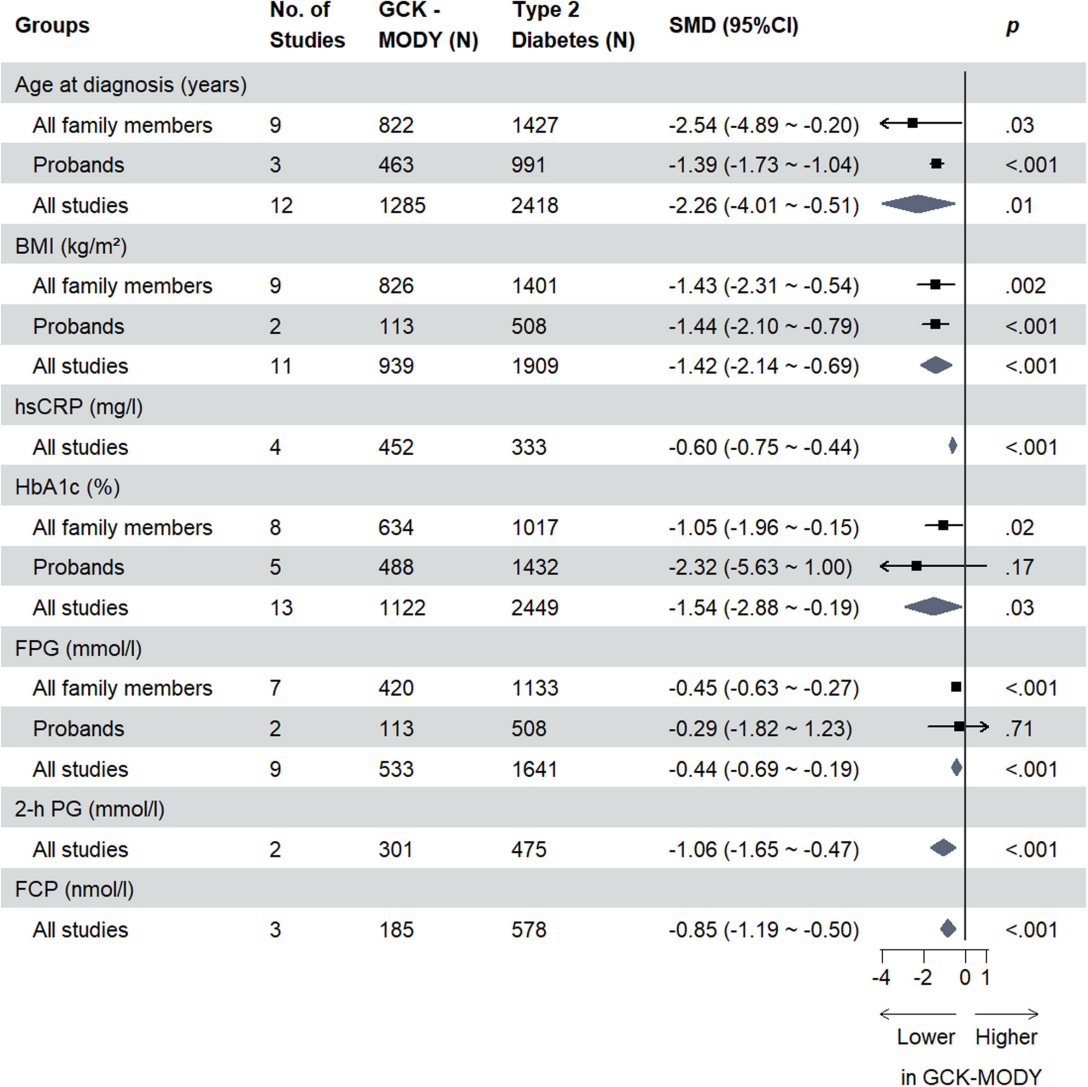
Significant clinical indicators in studies comparing GCK‐MODY and type 2 diabetes. 2‐h PG, 2‐hour postprandial glucose; BMI, body mass index; CI, confidence interval; FCP, fasting C‐peptide; FPG, fasting blood glucose; GCK, glucokinase; HbA1c, glycated hemoglobin; hsCRP, high‐sensitivity C‐reactive protein; MODY, maturity‐onset diabetes of the young; SMD, standardized mean difference.

For all clinical indicators assessed, the overall SMDs are presented in Tables 1 and 2, respectively. Indicators assessed in two or more studies with statistically significant results are shown in Figures 1 and 2. All forest plots relating to respective clinical indicators are provided in the Supplementary Figure S2–S30 in Appendix S1.

**TABLE 1:** The SMD effect of clinical indicators in studies comparing GCK‐MODY with HNF1A‐MODY.

Clinical indicators	No. of studies	GCK‐MODY (N)	HNF1A‐MODY (N)	SMD (95% CI)	*p*	I^2^	*P* ^*h*^
Age at diagnosis (years)	17	1222	548	−0.39 (−0.71, −0.07)	.02	84%	<.01
Birth Weight (kg)	5	318	81	−0.53 (−1.95, 0.88)	.46	96%	<.01
BMI (SDS)	5	245	131	−0.71 (−2.50, 1.08)	.44	98%	<.01
BMI (kg/m^2^)	15	1163	680	−0.38 (−0.69, −0.07)	.02	89%	<.01
hsCRP (mg/l)	3	192	132	0.31 (−0.25, 0.87)	.27	78%	.01
HbA1c (%)	18	990	497	−1.12 (−1.64, −0.60)	<.001	94%	<.01
FPG (mmol/l)	18	1042	455	−0.57 (−0.90, −0.23)	<.001	82%	<.01
2‐h PG (mmol/l)	5	567	166	−1.60 (−2.44, −0.76)	<.001	82%	<.01
Δ2‐h PG (mmol/l)	3	181	41	−1.49 (−1.98, −1.01)	<.001	0%	.37
FCP (nmol/l)	9	445	245	−0.06 (−0.24, 0.11)	.49	36%	.13
2‐h CP (nmol/l)	1	106	34	0.44 (0.55, 0.83)	.03	N/A	N/A
Insulin (mIU/l)	6	449	203	0.18 (−0.21, 0.58)	.36	66%	.01
2‐h insulin (mIU/l)	2	311	114	0.43 (0.22, 0.65)	<.001	0%	.56
TC (mmol/l)	10	631	299	−0.27 (−0.55, 0.01)	.06	58%	.01
LDL (mmol/l)	7	455	220	0.06 (−0.17, 0.29)	.59	14%	.32
HDL (mmol/l)	9	619	280	0.30 (−0.36, 0.97)	.38	95%	<.01
TG (mmol/l)	10	623	304	−0.93 (−1.66, −0.21)	.01	96%	<.01

Abbreviations: 2‐h CP, 2‐hour C‐peptide; 2‐h insulin, 2‐hour insulin; 2‐h PG, 2‐hour postprandial glucose; BMI, body mass, index; CI, confidence interval; FCP, fasting C‐peptide; FPG, fasting blood glucose; GCK, glucokinase; HbA1c, glycated hemoglobin; HDL, high‐density lipoprotein; HNF1A, hepatocyte nuclear factor 1‐α; hsCRP, high‐sensitivity C‐reactive protein; LDL, low‐density lipoprotein; MODY, maturity‐onset diabetes of the young; SDS, standardized; SMD, standardized mean difference; TC, total cholesterol; TG, total triglycerides; Δ2‐h PG, change in 2‐h PG.

**TABLE 2:** The SMD effect of clinical indicators in studies comparing GCK‐MODY with type 2 diabetes.

Clinical indicators	No. of studies	GCK‐MODY (N)	Type 2 diabetes (N)	SMD (95% CI)	*p*	I^2^	*P* ^*h*^
Age at diagnosis (years)	12	1285	2418	−2.26 (−4.01, −0.51)	.01	99%	<.01
BMI (SDS)	3	364	1335	−11.79 (−31.68, 8.11)	.25	100%	<.01
BMI (kg/m^2^)	11	939	1909	−1.42 (−2.14, −0.69)	<.001	97%	<.01
hsCRP (mg/l)	4	452	333	−0.60 (−0.75, −0.44)	<.001	13%	.33
HbA1c (%)	13	1122	2449	−1.54 (−2.88, −0.19)	.03	99%	<.01
FPG (mmol/l)	9	533	1641	−0.44 (−0.69, −0.19)	<.001	69%	<.01
2‐h PG (mmol/l)	2	301	475	−1.06 (−1.65, −0.47)	<.001	90%	<.01
Δ2‐h PG (mmol/l)	1	106	82	−1.02 (−1.32, −0.71)	<.001	N/A	N/A
FCP (nmol/l)	3	185	578	−0.85 (−1.19, −0.50)	<.001	50%	.13
2‐h CP (nmol/l)	1	106	82	−0.71 (−1.01, −0.41)	<.001	N/A	N/A
Insulin (mIU/l)	3	211	1149	−0.60 (−1.24, 0.04)	.07	77%	.01
2‐h insulin (mIU/l)	1	195	393	−0.57 (−0.74, −0.39)	<.001	N/A	N/A
TC (mmol/l)	6	392	1494	−1.05 (−2.60, 0.49)	.18	99%	<.01
LDL (mmol/l)	6	392	1494	−0.75 (−2.11, 0.61)	.28	99%	<.01
HDL (mmol/l)	5	383	1164	2.33 (−0.66, 5.32)	.13	100%	<.01
TG (mmol/l)	6	392	1494	−2.92 (−6.81, 0.97)	.14	100%	<.01

Abbreviations: 2‐h CP, 2‐hour C‐peptide; 2‐h insulin, 2‐hour insulin; 2‐h PG, 2‐hour postprandial glucose; BMI, body mass index; CI, confidence interval; FCP, fasting C‐peptide; FPG, fasting blood glucose; GCK, glucokinase; HbA1c, glycated hemoglobin; HDL, high‐density lipoprotein; hsCRP, high‐sensitivity C‐reactive protein; LDL, low‐density lipoprotein; MODY, maturity‐onset diabetes of the young; SDS, standardized; SMD, standardized mean difference; N/A, not applicable; TC, total cholesterol; TG, total triglycerides; Δ2‐h PG, change in 2‐h PG.

### 3.1 Bias assessment

Except for the “Exposure” indicator that addressed which item was evaluated in relation to each clinical indicator in each article (Supplementary Table S6 and S7), there were 6 studies (6 of 23) that scored 6 full stars, 5 studies (5 of 23) that scored 0, and 12 studies that scored between 1 and 5 stars according to the Newcastle‐Ottawa criteria in the HNF1A‐MODY group (Supplementary Table S8 in Appendix S1). For type 2 diabetes studies, these numbers were 2, 2, and 10 (of 14 studies), respectively (Supplementary Table S9 in Appendix S1). In the HNF1A‐MODY studies, most had a risk of bias due to case or control definitions (17 of 23), but some studies had risks of bias for evaluation items such as selection of controls (11 of 23), representatives of cases (10 of 23), and comparability (7 of 23). For type 2 diabetes studies, these numbers were 10, 6, 5, and 4 (of 14 studies), respectively.

According to the GRADE guidelines, for clinical indicators assessed in HNF1A‐MODY studies, there was only one1 indicator rated as “high quality of evidence,” whereas three, six, and five indicators were rated as “moderate,” “low,” and “very low” evidence levels, respectively. For type 2 diabetes studies, these numbers were 0, 1, 11, and 1, respectively (Supplementary Table S10 and S11 in Appendix S1).

### 3.2 Baseline differences between GCK‐MODY and HNF1A‐MODY/type 2 diabetes

GCK‐MODY patients were younger on average than those with HNF1A‐MODY (SMD −0.39 [95% CI −0.71, −0.07] years, *p* = .02, *n* = 17; I^2^ = 84%, *P* ^*h*^ <.01) or type 2 diabetes (SMD −2.26 [95% CI −4.01, −0.51] years, *p* = .01, *n* = 12; I^2^ = 99%, *P* ^*h*^ <.01) at the time of diagnosis. Except for the subgroup of studies that contained all family members and compared GCK‐MODY and HNF1A‐MODY, this result was consistent within subgroup analyses of probands and all family members. The difference in birthweight between GCK‐MODY and HNF1A‐MODY patients was not statistically significant (SMD −0.53 [95% CI −1.95, 0.88] kg, *p* = .46, *n* = 5; I^2^ = 96%, *P* ^*h*^ <.01), but this indicator was only involved in the proband HNF1A‐MODY studies. No included studies compared birthweight between GCK‐MODY and type 2 diabetes patients.

The difference in BMI in pediatric patients, expressed as a *Z*‐score, did not attain statistical significance for studies that compared GCK‐MODY and HNF1A‐MODY/type 2 diabetes, and this indicator was assessed only in proband studies. The pooled SMD for HNF1A‐MODY studies was −0.71 (95% CI −2.50, 1.08) (*p* = .44, *n* = 5; I^2^ = 98%, *P* ^*h*^ <.01) and was −11.79 (95% CI −31.68, 8.11) (*p* = .25, *n* = 3; I^2^ = 100%, *P* ^h^ <.01) for type 2 diabetes studies.

Differences in BMI, expressed in kilograms per meters squared, were statistically significant between GCK‐MODY and HNF1A‐MODY (SMD −0.38 [95% CI −0.69, −0.07] kg/m^2^, *p* = .02, *n* = 15; I^2^ = 89%, *P* ^*h*^ <.01). However, in our subgroup analysis, this difference was concordant only with the subgroup of studies that contained probands, not those that contained all family members. Because the article of Bacon et al^45^ matched BMI among patients, we excluded this study when analyzing the BMI parameter. In type 2 diabetes studies, the difference in BMI was consistent between studies of probands and those that included all family members (overall SMD −1.42 [95% CI −2.14, −0.69] kg/m^2^, *p* < .001, *n* = 11; I^2^ = 97%, *P* ^*h*^ <.01).

GCK‐MODY patients had lower hsCRP compared to type 2 diabetes patients (SMD −0.60 [95% CI −0.75, −0.44] mg/l, *p* < .001, *n* = 4; I^2^ = 13%, *P* ^*h*^ = .33), regardless of patient type, but for HNF1A‐MODY studies there was no distinct difference in hsCRP compared to HNF1A‐MODY (SMD 0.31 [95% CI −0.25, 0.87] mg/l, *p* = .27, *n* = 3; I^2^ = 78%, *P* ^*h*^ = .01).

### 3.3 Glucose metabolism differences between GCK‐MODY and HNF1A‐MODY/type 2 diabetes

GCK‐MODY patients had lower HbA1c compared to HNF1A‐MODY patients (SMD −1.12 [95% CI −1.64, −0.60] %, *p* < .001, *n* = 18; I^2^ = 94%, *P* ^*h*^ <.01), irrespective of patient type, and in type 2 diabetes studies, the difference was only significant for subgroups that contained all family members (overall SMD −1.54 [95% CI −2.88, −0.19] %, *p* = .03, *n* = 13; I^2^ = 99%, *P* ^*h*^ <.01).

FPG was lower for GCK‐MODY patients compared to those with HNF1A‐MODY (SMD −0.57 [95% CI −0.90, −0.23] mmol/l, *p* < .001, *n* = 18; I^2^ = 82%, *P* ^*h*^ <.01), which was consistent in the subgroup analysis, and we found a similar result for GCK‐MODY patients compared to type 2 diabetes patients (overall SMD −0.44 [95% CI −0.69, −0.19] mmol/l, *p* < .001, *n* = 9; I^2^ = 69%, *P* ^*h*^ <.01), though this difference was statistically significant only in the subgroup that contained all family members.

No studies compared 1‐hour postprandial glucose of GCK‐MODY with HNF1A‐MODY/type 2 diabetes. However, GCK‐MODY patients had lower 2‐h PG compared to both HNF1A‐MODY (SMD −1.60 [95% CI −2.44, −0.76] mmol/l, *p* < .001, *n* = 5; I^2^ = 82%, *P* ^*h*^ <.01) and type 2 diabetes patients (SMD −1.06 [95% CI −1.65, −0.47] mmol/l, *p* < .001, *n* = 2; I^2^ = 90%, *P* ^*h*^ <.01). For Δ2‐h PG, GCK‐MODY patients also had a lower Δ2‐h PG compared to both HNF1A‐MODY (SMD −1.49 [95% CI −1.98, −1.01] mmol/l, *p* < .001, *n* = 3; I^2^ = 0%, *P* ^*h*^ = .37) and type 2 diabetes (SMD −1.02 [95% CI −1.32, −0.71] mmol/l, *p* < .001, *n* = 1) as well.

Results for fasting C‐peptide (FCP) did not show a statistically significant difference between GCK‐MODY and HNF1A‐MODY patients (SMD −0.06 [95% CI −0.24, 0.11] nmol/l, *p* = .49, *n* = 9; I^2^ = 36%, *P* ^*h*^ = .13), regardless of whether the studies contained family members or not. By contrast, FCP was lower in GCK‐MODY patients than in type 2 diabetes patients (SMD −0.85 [95% CI −1.19, −0.50] nmol/l, *p* < .001, *n* = 3; I^2^ = 50%, *P* ^*h*^ = .13). Only one study compared 2‐hour C‐peptide (2‐h CP) between GCK‐MODY and HNF1A‐MODY (SMD 0.44 [95% CI 0.05, 0.83] nmol/l, *p* = .03, *n* = 1)/type 2 diabetes (SMD −0.71 [95% CI −1.01, −0.41] nmol/l, *p* < .001, *n* = 1) patients.

We observed no statistical differences for fasting insulin (HNF1A‐MODY studies: SMD 0.18 [95% CI −0.21, 0.58] mIU/l, *p* = .36, *n* = 6; I^2^ = 66%, *P* ^*h*^ = .01; type 2 diabetes studies: SMD −0.60 [95% CI −1.24, 0.04] mIU/l, *p* = .07, *n* = 3; I^2^ = 77%, *P* ^*h*^ = .01). For 2‐hour insulin, GCK‐MODY patients had higher insulin concentration on average compared to HNF1A‐MODY patients (SMD 0.43 [95% CI 0.22, 0.65] mIU/l, *p* < .001, *n* = 2; I^2^ = 0%, *P* ^*h*^ = .56) but had lower insulin concentration compared to type 2 diabetes patients (SMD −0.57 [95% CI −0.74, −0.39] mIU/l, *p* < .001, *n* = 1).

### 3.4 Lipid metabolism differences between GCK‐MODY and HNF1A‐MODY/type 2 diabetes

Except for the result that TG was lower in GCK‐MODY patients compared to those with HNF1A‐MODY (SMD −0.93 [95% CI −1.66, −0.21] mmol/l, *p* = .01, *n* = 10; I^2^ = 96%, *P* ^*h*^ <.01), there were no statistical differences observed in regard to other lipid indices in studies that compared GCK‐MODY with HNF1A‐MODY/type 2 diabetes.

### 3.5 Publication bias and sensitivity analysis

The funnel plots and Egger's regression showed that there was no risk of publication bias for all clinical indicators that were assessed in 10 or more studies (all contour‐enhanced funnel plots and trim‐and‐fill funnel plots are available in the Supplementary Figures S31–S48 in Appendix S1).

## 4 DISCUSSION

To the best of our knowledge, this meta‐analysis is the first article to compare results from multiple countries for routine examinations that involved indicators for baseline data and glucose and lipid metabolism between GCK‐MODY, HNF1A‐MODY, and type 2 diabetes, as well as the difference between proband and all‐family‐members subgroups. The results strengthen and expand on previous studies. Because the included studies all used methods of genetic testing to confirm the diagnoses of monogenic diabetes, and because almost all studies recruited patients who met the criteria for MODY or recruited type 2 diabetes who had good glycemic control or had no vascular complications, we expect our pooled results to be suitable for health practitioners trying to diagnose patients who exhibit similar characteristics to MODY.^49^ We also expect that our research can help increase the probability of accurately diagnosing subtypes of diabetes and giving appropriate genetic testing suggestions to target patients.

First, for discrimination between GCK‐MODY and HNF1A‐MODY, indices of glucose metabolism such as lower HbA1c, lower FPG, lower 2‐h PG, and lower Δ2‐h PG may help physicians to identify GCK‐MODY probands in the early stage, and in the follow‐up period, lower TG may strengthen this diagnosis. Second, GCK‐MODY may exhibit younger onset age, lower BMI, lower FCP, lower 2‐h PG, and hsCRP compared to type 2 diabetes, especially for patients who almost meet the criteria of MODY in the early stage. Lower HbA1c and FPG might also further help to discriminate GCK‐MODY from type 2 diabetes after a follow‐up period. According to the data (Supplementary Tables S12–S15 in Appendix S1), studies that contained all family members involved patients with a longer diabetic duration and older age at recruitment compared to studies that contained only probands. This finding is likely due to most studies' specific inclusion criteria for MODY patients and the exclusion of MODY probands who developed hyperglycemia after 25 years of age, which were in accord with the diagnostic criteria of MODY,^49^ resulting in a younger average age and shorter average duration for the proband group compared to the all‐family‐members group in our study. As a result, we interpreted subgroups with all family members or probands as patients with a longer duration of diabetes or in the early stage of diabetes, respectively. This assumption also accords with the phenomenon that patients in a family with a genetic disease often have an earlier visit to the hospital relative to their older generations due to increased health consciousness on the part of the younger members.

Our study showed that probands with GCK‐MODY were about 0.65 years younger than those with HNF1A‐MODY at diagnosis. Combined with the result that GCK‐MODY probands were about 0.87 years younger at recruitment compared to those with HNF1A‐MODY (Supplementary Figure S49 in Appendix S1), we speculate that there may be no actual difference in age at diagnosis of diabetes between patients with GCK‐MODY and those with HNF1A‐MODY. The overall SMD of age at diagnosis (around 0.39 years younger for GCK‐MODY patients) and age at recruitment (around 0.63 years younger for GCK‐MODY patients) also indicate that this may be the case. For studies comparing GCK‐MODY to type 2 diabetes, the results showed that patients with the *GCK* mutation were around 2.46 years younger than type 2 diabetes patients at diagnosis, which agrees with the typically younger‐onset age for MODY patients compared to type 2 diabetes.

The birthweight of GCK‐MODY was not statistically different from that of HNF1A‐MODY. Furthermore, the BMI *Z*‐score results showed that for pediatric patients who almost met MODY criteria, patients with GCK‐MODY, HNF1A‐MODY, or type 2 diabetes did not have not a statistically significant difference in growth stages. The BMI (kg/m^2^) results themselves showed that probands with GCK‐MODY were lighter on average than HNF1A‐MODY probands. However, this is to be expected considering that GCK‐MODY subjects were also younger than HNF1A‐MODY patients in almost all studies involved (Supplementary Figure S49 in Appendix S1), and we think the younger age difference may partly account for the lower BMI result. For BMI (kg/m^2^) differences between GCK‐MODY and type 2 diabetes patients, our results are consistent with a previous study done by Shields et al^9^ that found that GCK‐MODY patients have lower BMIs than type 2 diabetes patients, and this may be due to the fact that type 2 diabetes patients are typically overweight.

This meta‐analysis also found that hsCRP was lower in GCK‐MODY patients than in type 2 diabetes patients, which suggests that GCK‐MODY has lower inflammation levels than type 2 diabetes. In past research, HNF1A‐MODY has been found to have lower hsCRP compared to other diabetic forms^6^ because HNF1 is the transactivating factor of CRP promoter.^50^ However, the pathogenicity of gene mutation seems to correlate with the level of hsCRP,^8^ and the HNF1A‐MODY studies that involved the hsCRP parameter are few, which may explain the reason for the statistically insignificant result for hsCRP in HNF1A‐MODY studies.

Surprising results came from glucose metabolism indicators. GCK‐MODY is already known to cause mild hyperglycemia, and our study results also showed that there were lower HbA1c and lower FPG of GCK‐MODY compared to HNF1A‐MODY in both subgroups. However, we found that in the early stage, HbA1c might not help to distinguish GCK‐MODY patients from type 2 diabetes patients who also almost met the criteria for MODY and that GCK‐MODY likely exhibits lower HbA1c compared to type 2 diabetes in the follow‐up period. We also reached the same conclusion for the outcomes in FPG compared GCK‐MODY patients with type 2 diabetes patients.

The results in FCP are consistent with a previous study^7^ that found that the concentration of FCP was higher in type 2 diabetes patients, reflecting the insulin resistance inherent to type 2 diabetes, which was also exhibited in the 2‐h CP result. However, no significant difference in FCP was observed between the two MODY subtypes when taking the higher blood glucose presented by HNF1A‐MODY patients into consideration (Supplementary Figures S11,S13,S15,S17). This result may indicate that the islet function of HNF1A‐MODY patients in the early stage is not sufficient to lower blood glucose compared to that of GCK‐MODY patients. The result of higher 2‐h CP in GCK‐MODY patients compared to HNF1A‐MODY patients also agrees with this conclusion in the context of higher 2‐h PG in HNF1A‐MODY patients. Combined with the results for 2‐h PG and Δ2‐h PG, we can presume that GCK‐MODY patients have a better glucose metabolism compared to those with HNF1A‐MODY and type 2 diabetes; they exhibit lower glucose excursion, which means their islet cells function well in antagonizing postprandial hyperglycemia. However, the results on insulin are only ancillary to these conclusions; for some of the patients who took insulin, we cannot accurately derive any metabolic information from this adjunctive measurement.

From the results of lipid metabolism, we found that GCK‐MODY patients have lower TG than HNF1A‐MODY patients in the follow‐up period, and comparison with type 2 diabetes patients also showed a tendency toward lower TG in GCK‐MODY in the all‐family‐members subgroup (with longer diabetes duration). Other clinical^48^, ^51^, ^52^ experiments have also shown decreased TG and free fatty acid in GCK‐MODY compared to normal individuals or those with other types of diabetes, and our team found that localized increases of choline/ethanolamine phosphotransferase 1 and adipose triglyceride lipase in HDLs may explain this phenomenon.^51^

TC and LDL were not suitable indicators for physicians to distinguish between the three types of diabetes of interest. For HDL however, this adjunctive examination is not helpful in distinguishing GCK‐MODY from HNF1A‐MODY, but its difference may be helpful in distinguishing GCK‐MODY from type 2 diabetes. In the subgroup with all family members, HDL was higher in GCK‐MODY patients compared to type 2 diabetes patients. Our previous studies have also shown that HDL is higher in GCK‐MODY than in type 2 diabetes.^51^ However, the number of studies in this meta‐analysis is most likely insufficient to highlight this difference. What's more, the study of Arslanian et al^20^ had clearly imbalanced group numbers between GCK‐MODY and type 2 diabetes patients and a very small number of GCK‐MODY patients, so ideally we can involve more eligible studies in the future to help reveal the underlying HDL difference between these two diseases.

This study has some limitations, however. First, for lipid metabolism and other indicators, there are limited studies that have reported such results. Thus, the pooled results may be more robust in the future when there are more studies that present these clinical results. Second, some studies contained patients with variants of uncertain significance, which may bias the characteristics of monogenic diabetes. Third, for the intrinsic traits and goals of the involved studies, it is only possible to examine potential useful indicators to help with diagnosis. For discriminatory abilities such as the sensitivity and specificity of these indicators, more focused diagnostic trials are needed to give useful receiver operating characteristics analysis results. Furthermore, the value of potential indicators needs to be validated and compared in diagnostic trials. In the future, studies that exert rigorous genetic standards for confirmation of genetic diabetes and report more results of basic adjunctive measurements like lipid metabolism are necessary to give further insight into the differences between GCK‐MODY and HNF1A‐MODY/type 2 diabetes. Last, we excluded the pregnant population in this study for the specific inclusion criteria of most studies, but this special population deserves more attention for their different treatment strategies, and focus is needed to put on the fetal abdominal circumference, blood glucose of the mother,^53^, ^54^, ^55^ and so on in future studies.

In summary, this study is the first worldwide meta‐analysis to explore the differences in basic clinical results between GCK‐MODY, HNF1A‐MODY, and type 2 diabetes. In the early period of diabetes, age, BMI, hsCRP, FCP, and 2‐h PG may help to distinguish GCK‐MODY from type 2 diabetes with good glycemic control or diagnosis at a young age, and results of HbA1c and FPG may be good indicators for this distinction after a long follow‐up period. When distinguishing GCK‐MODY from HNF1A‐MODY, indicators of glucose metabolism may be helpful at an early period, and TG may be useful after a long follow‐up period.

## AUTHOR CONTRIBUTIONS

J.L. and X.H.X. conceived and designed the study. All authors were involved in the acquisition, analysis, and interpretation of data. J.L. drafted the manuscript. X.H.X., Q.Z., and M.Y. contributed to the critical revision of the manuscript for important intellectual content. All authors approved the final manuscript for publication. X.H.X is the guarantor, maintaining full access to all the data in the study and taking responsibility for the integrity of the data and the accuracy of the analysis.

## FUNDING INFORMATION

The funding sources did not play a role in the design or execution of the study, including the collection, management, analysis, and interpretation of the data; the preparation, review, and approval of the manuscript; and the decision to submit the manuscript for publication.

## CONFLICT OF INTEREST STATEMENT

The authors declare that they have no conflicts of interest.

## Supporting information


**Appendix S1.** Supporting Information.

**Table S6.** Bias assessment of the “Exposure” item for each clinical indicator in hepatocyte nuclear factor 1‐α‐maturity‐onset diabetes of the young (HNF1A‐MODY) studies.

**Table S7.** Bias assessment of the “Exposure” item for each clinical indicator in type 2 diabetes studies.
