# Supplementary material for: Insights from basic adjunctive examinations of GCK‐MODY, HNF1A‐MODY, and type 2 diabetes: A systemic review and meta‐analysis
Source: J Diabetes. 2023 May 24;15(6):519–31. doi: 10.1111/1753-0407.13390 (PMC10270746; doi:10.1111/1753-0407.13390)
Supplement: Supplementary file 1 — Appendix S1. Supporting Information. [file JDB-15-519-s002.docx]

**Supplementary Material**

**Insights from Basic Adjunctive Examinations of GCK-MODY, HNF1A-MODY, and Type 2 Diabetes: A Systemic Review and Meta-Analysis**

Jing Liu ^1^, Xinhua Xiao ^1^, Qian Zhang ^1^, Miao Yu ^1^

1 Key Laboratory of Endocrinology of National Health Commission, Diabetes Research Center of Chinese Academy of Medical Sciences, Department of Endocrinology, Translational Medicine Center, Peking Union Medical College Hospital, Chinese Academy of Medical Sciences and Peking Union Medical College, Beijing, 100730, People’s Republic of China

**CONTENTS**

[Table S1. PRISMA 2020 Checklist 5](#_Toc115721428)

[Table S2. MOOSE Checklist 10](#_Toc115721429)

[Table S3. Data sources and search strategies 13](#_Toc115721430)

[Figure S1. PRISMA 2020 flow diagram 14](#_Toc115721431)

[Table S4. Characteristics of included articles 15](#_Toc115721432)

[Forest Plots for Baseline Differences between GCK-MODY and HNF1A-MODY/Type 2 Diabetes 18](#_Toc115721433)

[Figure S2. The forest plot for age at diagnosis in HNF1A-MODY studies 18](#_Toc115721434)

[Figure S3. The forest plot for age at diagnosis in type 2 diabetes studies 18](#_Toc115721435)

[Figure S4. The forest plot for birthweight in HNF1A-MODY studies 19](#_Toc115721436)

[Figure S5. The forest plot for BMI (SDS) in HNF1A-MODY studies 19](#_Toc115721437)

[Figure S6. The forest plot for BMI (SDS) in type 2 diabetes studies 19](#_Toc115721438)

[Figure S7. The forest plot for BMI (kg/m^2^) in HNF1A-MODY studies 20](#_Toc115721439)

[Figure S8. The forest plot for BMI (kg/m^2^) in type 2 diabetes studies 20](#_Toc115721440)

[Figure S9. The forest plot for hsCRP in HNF1A-MODY studies 21](#_Toc115721441)

[Figure S10. The forest plot for hsCRP in type 2 diabetes studies 21](#_Toc115721442)

[Forest Plots for Glucose Metabolism Differences between GCK-MODY and HNF1A-MODY/Type 2 Diabetes 22](#_Toc115721443)

[Figure S11. The forest plot for HbA1c in HNF1A-MODY studies 22](#_Toc115721444)

[Figure S12. The forest plot for HbA1c in type 2 diabetes studies 22](#_Toc115721445)

[Figure S13. The forest plot for fasting blood glucose in HNF1A-MODY studies 23](#_Toc115721446)

[Figure S14. The forest plot for fasting blood glucose in type 2 diabetes studies 23](#_Toc115721447)

[Figure S15. The forest plot for 2-hour postprandial glucose in HNF1A-MODY studies 24](#_Toc115721448)

[Figure S16. The forest plot for 2-hour postprandial glucose in type 2 diabetes studies 24](#_Toc115721449)

[Figure S17. The forest plot for change in 2-hour postprandial glucose in HNF1A-MODY studies 24](#_Toc115721450)

[Figure S18. The forest plot for fasting C peptide in HNF1A-MODY studies 25](#_Toc115721451)

[Figure S19. The forest plot for fasting C peptide in type 2 diabetes studies 25](#_Toc115721452)

[Figure S20. The forest plot for fasting insulin concentration in HNF1A-MODY studies 26](#_Toc115721453)

[Figure S21. The forest plot for fasting insulin concentration in type 2 diabetes studies 26](#_Toc115721454)

[Figure S22. The forest plot for 2-hour insulin concentration in HNF1A-MODY studies 26](#_Toc115721455)

[Forest Plots for Lipid Metabolism Differences between GCK-MODY and HNF1A-MODY/Type 2 Diabetes 27](#_Toc115721456)

[Figure S23. The forest plot for TC in HNF1A-MODY studies 27](#_Toc115721457)

[Figure S24. The forest plot for TC in type 2 diabetes studies 27](#_Toc115721458)

[Figure S25. The forest plot for HDL in HNF1A-MODY studies 28](#_Toc115721459)

[Figure S26. The forest plot for HDL in type 2 diabetes studies 28](#_Toc115721460)

[Figure S27. The forest plot for LDL in HNF1A-MODY studies 29](#_Toc115721461)

[Figure S28. The forest plot for LDL in type 2 diabetes studies 29](#_Toc115721462)

[Figure S29. The forest plot for TG in HNF1A-MODY studies 30](#_Toc115721463)

[Figure S30. The forest plot for TG in type 2 diabetes studies 30](#_Toc115721464)

[Materials Relating with the Newcastle-Ottawa Criteria 31](#_Toc115721465)

[Table S5. Definition of terms for bias assessment according to the Newcastle-Ottawa criteria for case-control studies 31](#_Toc115721466)

[Bias assessment according to the Newcastle-Ottawa criteria for case-control studies 33](#_Toc115721467)

[Table S8. HNF1A-MODY studies except the “Exposure” items 33](#_Toc115721468)

[Table S9. Type 2 diabetes studies except the “Exposure” items 35](#_Toc115721469)

[GRADE Profiles 36](#_Toc115721470)

[Table S10. GRADE evidence profile for clinical indicators quantified in ≧2 studies in HNF1A-MODY studies 36](#_Toc115721471)

[Table S11. GRADE evidence profile for clinical indicators quantified in ≧2 studies in type 2 diabetes studies 37](#_Toc115721472)

[Funnel Plots for Publication Bias and Sensitivity Analysis 39](#_Toc115721473)

[HNF1A-MODY studies 39](#_Toc115721474)

[Figure S31. The contour-enhanced funnel plot for age at diagnosis in HNF1A-MODY studies 39](#_Toc115721475)

[Figure S32. The trim-and-fill funnel plot for age at diagnosis in HNF1A-MODY studies 39](#_Toc115721476)

[Figure S33. The contour-enhanced funnel plot for BMI (kg/m^2^) in HNF1A-MODY studies 40](#_Toc115721477)

[Figure S34. The trim-and-fill funnel plot for BMI (kg/m^2^) in HNF1A-MODY studies 40](#_Toc115721478)

[Figure S35. The contour-enhanced funnel plot for HbA1c in HNF1A-MODY studies 41](#_Toc115721479)

[Figure S36. The trim-and-fill funnel plot for HbA1c in HNF1A-MODY studies 41](#_Toc115721480)

[Figure S37. The contour-enhanced funnel plot for fasting blood glucose in HNF1A-MODY studies 42](#_Toc115721481)

[Figure S38. The trim-and-fill funnel plot for fasting blood glucose in HNF1A-MODY studies 42](#_Toc115721482)

[Figure S39. The contour-enhanced funnel plot for TC in HNF1A-MODY studies 43](#_Toc115721483)

[Figure S40. The trim-and-fill funnel plot for TC in HNF1A-MODY studies 43](#_Toc115721484)

[Figure S41. The contour-enhanced funnel plot for TG in HNF1A-MODY studies 44](#_Toc115721485)

[Figure S42. The trim-and-fill funnel plot for TG in HNF1A-MODY studies 44](#_Toc115721486)

[Type 2 diabetes studies 45](#_Toc115721487)

[Figure S43. The contour-enhanced funnel plot for age at diagnosis in type 2 diabetes studies 45](#_Toc115721488)

[Figure S44. The trim-and-fill funnel plot for age at diagnosis in type 2 diabetes studies 45](#_Toc115721489)

[Figure S45. The contour-enhanced funnel plot for BMI (kg/m^2^) in type 2 diabetes studies 46](#_Toc115721490)

[Figure S46. The trim-and-fill funnel plot for BMI (kg/m^2^) in type 2 diabetes studies 46](#_Toc115721491)

[Figure S47. The contour-enhanced funnel plot for HbA1c in type 2 diabetes studies 47](#_Toc115721492)

[Figure S48. The trim-and-fill funnel plot for HbA1c in type 2 diabetes studies 47](#_Toc115721493)

[Materials for Other Indicators in Three Diabetic Subtypes 48](#_Toc115721494)

[Table S12. Diabetes duration of patients in HNF1A-MODY studies 48](#_Toc115721495)

[Table S13. Diabetes duration of patients in type 2 diabetes studies 48](#_Toc115721496)

[Table S14. Age at recruitment of patients in HNF1A-MODY studies 48](#_Toc115721497)

[Table S15. Age at recruitment of patients in type 2 diabetes studies 48](#_Toc115721498)

[Figure S49. The forest plot for age at recruitment in HNF1A-MODY studies 49](#_Toc115721499)

[Figure S50. The forest plot for age at recruitment in type 2 diabetes studies 49](#_Toc115721500)

[Reference 50](#_Toc115721501)

# Table S1. PRISMA 2020 Checklist

PRISMA 2020 Main Checklist

| **Topic** | **No.** | **Item** | **Location where item is reported** |
| --- | --- | --- | --- |
| **TITLE** |  |  |  |
| **Title** | 1 | Identify the report as a systematic review. | p1 |
| **ABSTRACT** |  |  |  |
| **Abstract** | 2 | See the PRISMA 2020 for Abstracts checklist |  |
| **INTRODUCTION** |  |  |  |
| **Rationale** | 3 | Describe the rationale for the review in the context of existing knowledge. | p4-p5 |
| **Objectives** | 4 | Provide an explicit statement of the objective(s) or question(s) the review addresses. | p5 |
| **METHODS** |  |  |  |
| **Eligibility criteria** | 5 | Specify the inclusion and exclusion criteria for the review and how studies were grouped for the syntheses. | p5-p6 |
| **Information sources** | 6 | Specify all databases, registers, websites, organisations, reference lists and other sources searched or consulted to identify studies. Specify the date when each source was last searched or consulted. | p6,  Table S3,  Figure S1 |
| **Search strategy** | 7 | Present the full search strategies for all databases, registers and websites, including any filters and limits used. | Table S3 |
| **Selection process** | 8 | Specify the methods used to decide whether a study met the inclusion criteria of the review, including how many reviewers screened each record and each report retrieved, whether they worked independently, and if applicable, details of automation tools used in the process. | p7-p8 |
| **Data collection process** | 9 | Specify the methods used to collect data from reports, including how many reviewers collected data from each report, whether they worked independently, any processes for obtaining or confirming data from study investigators, and if applicable, details of automation tools used in the process. | p7-p8 |
| **Data items** | 10a | List and define all outcomes for which data were sought. Specify whether all results that were compatible with each outcome domain in each study were sought (e.g. for all measures, time points, analyses), and if not, the methods used to decide which results to collect. | p7 |
|  | 10b | List and define all other variables for which data were sought (e.g. participant and intervention characteristics, funding sources). Describe any assumptions made about any missing or unclear information. | p7 |
| **Study risk of bias assessment** | 11 | Specify the methods used to assess risk of bias in the included studies, including details of the tool(s) used, how many reviewers assessed each study and whether they worked independently, and if applicable, details of automation tools used in the process. | p8 |
| **Effect measures** | 12 | Specify for each outcome the effect measure(s) (e.g. risk ratio, mean difference) used in the synthesis or presentation of results. | p8-p9 |
| **Synthesis methods** | 13a | Describe the processes used to decide which studies were eligible for each synthesis (e.g. tabulating the study intervention characteristics and comparing against the planned groups for each synthesis (item 5)). | p7, p9, Table S4 |
|  | 13b | Describe any methods required to prepare the data for presentation or synthesis, such as handling of missing summary statistics, or data conversions. | p8 |
|  | 13c | Describe any methods used to tabulate or visually display results of individual studies and syntheses. | p8 |
|  | 13d | Describe any methods used to synthesize results and provide a rationale for the choice(s). If meta-analysis was performed, describe the model(s), method(s) to identify the presence and extent of statistical heterogeneity, and software package(s) used. | p8-p9 |
|  | 13e | Describe any methods used to explore possible causes of heterogeneity among study results (e.g. subgroup analysis, meta-regression). | p8 |
|  | 13f | Describe any sensitivity analyses conducted to assess robustness of the synthesized results. | p8 |
| **Reporting bias assessment** | 14 | Describe any methods used to assess risk of bias due to missing results in a synthesis (arising from reporting biases). | p8,  Table S5 |
| **Certainty assessment** | 15 | Describe any methods used to assess certainty (or confidence) in the body of evidence for an outcome. | p8 |
| **RESULTS** |  |  |  |
| **Study selection** | 16a | Describe the results of the search and selection process, from the number of records identified in the search to the number of studies included in the review, ideally using a flow diagram. | p9,  Figure S1 |
|  | 16b | Cite studies that might appear to meet the inclusion criteria, but which were excluded, and explain why they were excluded. | Figure S1 |
| **Study characteristics** | 17 | Cite each included study and present its characteristics. | Table S4 |
| **Risk of bias in studies** | 18 | Present assessments of risk of bias for each included study. | p10,  Table S5-S9 |
| **Results of individual studies** | 19 | For all outcomes, present, for each study: (a) summary statistics for each group (where appropriate) and (b) an effect estimate and its precision (e.g. confidence/credible interval), ideally using structured tables or plots. | Table 1, Table 2, Figure 1, Figure 2 |
| **Results of syntheses** | 20a | For each synthesis, briefly summarise the characteristics and risk of bias among contributing studies. | Table S4, Table S6-S9 |
|  | 20b | Present results of all statistical syntheses conducted. If meta-analysis was done, present for each the summary estimate and its precision (e.g. confidence/credible interval) and measures of statistical heterogeneity. If comparing groups, describe the direction of the effect. | p11-p14, Figure S2-S30 |
|  | 20c | Present results of all investigations of possible causes of heterogeneity among study results. | p11-p14, Figure S2-S30 |
|  | 20d | Present results of all sensitivity analyses conducted to assess the robustness of the synthesized results. | p14, Figure S31-S48 |
| **Reporting biases** | 21 | Present assessments of risk of bias due to missing results (arising from reporting biases) for each synthesis assessed. | p10,  Table S5-S9 |
| **Certainty of evidence** | 22 | Present assessments of certainty (or confidence) in the body of evidence for each outcome assessed. | p10-p11, Table S10-S11 |
| **DISCUSSION** |  |  |  |
| **Discussion** | 23a | Provide a general interpretation of the results in the context of other evidence. | p14-p19 |
|  | 23b | Discuss any limitations of the evidence included in the review. | p19 |
|  | 23c | Discuss any limitations of the review processes used. | p19 |
|  | 23d | Discuss implications of the results for practice, policy, and future research. | p19 |
| **OTHER INFORMATION** |  |  |  |
| **Registration and protocol** | 24a | Provide registration information for the review, including register name and registration number, or state that the review was not registered. | p1, p5 |
|  | 24b | Indicate where the review protocol can be accessed, or state that a protocol was not prepared. | p1, p5 |
|  | 24c | Describe and explain any amendments to information provided at registration or in the protocol. | p1, p5 |
| **Support** | 25 | Describe sources of financial or non-financial support for the review, and the role of the funders or sponsors in the review. | p20 |
| **Competing interests** | 26 | Declare any competing interests of review authors. | p21 |
| **Availability of data, code and other materials** | 27 | Report which of the following are publicly available and where they can be found: template data collection forms; data extracted from included studies; data used for all analyses; analytic code; any other materials used in the review. | p9 |

PRIMSA Abstract Checklist

| **Topic** | **No.** | **Item** | **Reported?** |
| --- | --- | --- | --- |
| **TITLE** |  |  |  |
| **Title** | 1 | Identify the report as a systematic review. | Yes |
| **BACKGROUND** |  |  |  |
| **Objectives** | 2 | Provide an explicit statement of the main objective(s) or question(s) the review addresses. | Yes |
| **METHODS** |  |  |  |
| **Eligibility criteria** | 3 | Specify the inclusion and exclusion criteria for the review. | Yes |
| **Information sources** | 4 | Specify the information sources (e.g. databases, registers) used to identify studies and the date when each was last searched. | Yes |
| **Risk of bias** | 5 | Specify the methods used to assess risk of bias in the included studies. | No |
| **Synthesis of results** | 6 | Specify the methods used to present and synthesize results. | Yes |
| **RESULTS** |  |  |  |
| **Included studies** | 7 | Give the total number of included studies and participants and summarise relevant characteristics of studies. | No |
| **Synthesis of results** | 8 | Present results for main outcomes, preferably indicating the number of included studies and participants for each. If meta-analysis was done, report the summary estimate and confidence/credible interval. If comparing groups, indicate the direction of the effect (i.e. which group is favoured). | Yes |
| **DISCUSSION** |  |  |  |
| **Limitations of evidence** | 9 | Provide a brief summary of the limitations of the evidence included in the review (e.g. study risk of bias, inconsistency and imprecision). | No |
| **Interpretation** | 10 | Provide a general interpretation of the results and important implications. | Yes |
| **OTHER** |  |  |  |
| **Funding** | 11 | Specify the primary source of funding for the review. | No |
| **Registration** | 12 | Provide the register name and registration number. | No |

*From:* Page MJ, McKenzie JE, Bossuyt PM, Boutron I, Hoffmann TC, Mulrow CD, et al. The PRISMA 2020 statement: an updated guideline for reporting systematic reviews. MetaArXiv. 2020, September 14. DOI: 10.31222/osf.io/v7gm2. For more information, visit: <www.prisma-statement.org>

# Table S2. MOOSE Checklist

From: Stroup DF, Berlin JA, Morton SC, et al (2000) Meta-analysis of observational studies in epidemiology: A proposal for reporting. JAMA 283:2008–2012. doi:10.1001/jama.283.15.2008.

| Item | Reported on page |
| --- | --- |
| **Reporting of background should include** | |
| Problem definition | p4-p5 |
| Hypothesis statement | p4 |
| Description of study outcome(s) | p5 |
| Type of exposure or intervention used | p5 |
| Type of study designs used | p5 |
| Study population | p5-p6 |
| **Reporting of search strategy should include** | |
| Qualifications of searchers (e.g. librarians and investigators) | p6 |
| Search strategy, including time period used in the synthesis and key words | p6, Table S3 |
| Effort to include all available studies, including contact with authors | p6 |
| Databases and registries searched | p6, Table S3, Figure S1 |
| Search software used, name and version, including special features used (e.g. explosion) | p6-p7, Table S3, Figure S1 |
| Use of hand searching (e.g. reference lists of obtained articles) | p6, Table S3, Figure S1 |
| List of citations located and those excluded, including justification | Table S4 |
| Method of addressing articles published in languages other than English | p6 |
| Method of handling abstracts and unpublished studies | p6 |
| Description of any contact with authors | p6 |
| **Reporting of methods should include** | |
| Description of relevance or appropriateness of studies assembled for assessing the hypothesis to be tested | p6 |
| Rationale for the selection and coding of data (e.g. sound clinical principles or convenience) | p7 |
| Documentation of how data were classified and coded (e.g. multiple raters, blinding and interrater reliability) | p7 |
| Assessment of confounding (e.g. comparability of cases and controls in studies where appropriate) | p8,  Table S5-S9 |
| Assessment of study quality, including blinding of quality assessors, stratification or regression on possible predictors of study results | p8,  Table S10-S11 |
| Assessment of heterogeneity | p8 |
| Description of statistical methods (e.g. complete description of fixed or random effects models, justification of whether the chosen models account for predictors of study results, dose-response models, or cumulative meta-analysis) in sufficient detail to be replicated | p8 |
| Provision of appropriate tables and graphics | Supplementary, Figure 1 and 2,  Table 1 and 2 |
| **Reporting of results should include** | |
| Graphic summarizing individual study estimates and overall estimate | Figure 1 and 2 |
| Table giving descriptive information for each study included | Table S4 |
| Results of sensitivity testing (e.g. subgroup analysis) | p11-p14,  Figure S2-S30 |
| Indication of statistical uncertainty of findings | Figure S2-S30,  Table 1 and 2 |
| **Reporting of discussion should include** | |
| Quantitative assessment of bias (e.g. publication bias) | p14,  Figure S31-S48 |
| Justification for exclusion (e.g. exclusion of non-English language citations) | p6, Figure S1 |
| Assessment of quality of included studies | p10,  Table S10-S11 |
| **Reporting of conclusions should include** | |
| Consideration of alternative explanations for observed results | p14-p19 |
| Generalization of the conclusions (i.e. appropriate for the data presented and within the domain of the literature review) | p14-p19 |
| Guidelines for future research | p14-p15,  p19 |
| Disclosure of funding source | p21 |

# Table S3. Data sources and search strategies

| Databases | Search strategies |
| --- | --- |
| Ovid MEDLINE(R)  (n = 1186)  2022/10/09    (Ovid 1946 to 2022/10/07) | ("Maturity onset diabetes of the young 2" or "Maturity-onset diabetes of the young 2" or GCK-MODY or GCK-DM or MODY2 or (GCK and MODY) or ((MODY or "monogenic diabet*" or "Maturity-onset diabetes of the young" or "Maturity onset diabetes of the young") and (biomarker* or diagno* or screen* or profile* or predict* or algorithm*))).ti,ab. not (animals not humans).sh. |
| Embase  (n = 2526)  2022/10/09 | ('maturity onset diabetes of the young 2':ti,ab,tt OR 'maturity-onset diabetes of the young 2':ti,ab,tt OR 'gck mody':ti,ab,tt OR 'gck dm':ti,ab,tt OR mody2:ti,ab,tt OR (gck:ti,ab,tt AND mody:ti,ab,tt) OR ((mody:ti,ab,tt OR 'monogenic diabet*':ti,ab,tt OR 'maturity-onset diabetes of the young':ti,ab,tt OR 'maturity onset diabetes of the young':ti,ab,tt) AND (biomarker*:ti,ab,tt OR diagno*:ti,ab,tt OR screen*:ti,ab,tt OR profile*:ti,ab,tt OR predict*:ti,ab,tt OR algorithm*:ti,ab,tt))) NOT (('animal experiment'/exp OR 'animal experiment') NOT ('human experiment'/exp OR 'human experiment' OR 'human'/exp OR 'human'))  In Advanced Option |
| Central  (n = 148)  2022/10/09 | "Maturity onset diabetes of the young 2" or "Maturity-onset diabetes of the young 2" or GCK-MODY or GCK-DM or MODY2 or (GCK and MODY) or ((MODY or "monogenic diabet*" or "Maturity-onset diabetes of the young" or "Maturity onset diabetes of the young") and (biomarker* or diagno* or screen* or profile* or predict* or algorithm*))  In Search manager |

# Figure S1. PRISMA 2020 flow diagram

**Identification of studies via databases and registers**

Records removed *before screening*:

Duplicate records removed (n = 923)

Records identified from:

Databases (n = 3,860)

**Identification**

Records excluded

(n = 2,379)

Records screened

(n = 2,937)

Reports sought for retrieval

(n = 558)

Reports not retrieved

(n = 14)

**Screening**

Reports excluded:

Review (n = 49)

Insufficient patients or clinical results (n = 121)

No MODY2 patients or involving pregnant patients or no separate data for MODY2 (n = 122)

No type 2 diabetes or HNF1A-MODY patients (n = 203)

No genetic testing for confirmation of MODY (n = 6)

Articles from same research teams (n = 43)

Reports assessed for eligibility

(n = 544)

Studies included in review (n = 32):

Type 2 diabetes studies (n = 14)

HNF1A-MODY studies (n = 23)

**Included**

*From:*  Page MJ, McKenzie JE, Bossuyt PM, Boutron I, Hoffmann TC, Mulrow CD, et al. The PRISMA 2020 statement: an updated guideline for reporting systematic reviews. BMJ 2021;372:n71. doi: 10.1136/bmj.n71

For more information, visit: <http://www.prisma-statement.org/>

# Table S4. Characteristics of included articles

| **Study** | **Year** | **Recurit-ment Duration** | **Region** | **Single center or Multicenter** | **Category of Patient** | **Group** | **MODY2 (N)** | **Male of MODY2 (%)** | **Compare disease** | **Compare group (N)** | **Male of Compare group (%)** |
| --- | --- | --- | --- | --- | --- | --- | --- | --- | --- | --- | --- |
| Bacon et al ^1^ | 2012 | N/A | Ireland | Single center | adult patients | All Families | 13 | N/A | HNF1A-MODY | 37 | N/A |
| Chèvre et al. ^2^ | 1998 | 1991-1997 | France, Brazil, Belgium | Single center | all-age | All Families | 297 | 149 (50.2%) | HNF1A-MODY | 109 | 49 (45.0%) |
| Costa et al. ^3^ | 2000 | N/A | Spain | Single center | all-age | All Families | 12 | 9 (75.0%) | HNF1A-MODY | 19 | 7 (36.8%) |
| Fendler et al. ^4^ | 2011 | 2009.10-2010.08 | Poland | Single center | all-age | All Families | 148 | 77 (52%) | HNF1A-MODY | 37 | 26 (70%) |
| Owen et al. ^5^ | 2010 | N/A | UK | Multicenter | adult patients | All Families | 24 | 10 (41.7%) | HNF1A-MODY | 31 | 11 (35.5%) |
| Nowak et al. ^6^ | 2013 | N/A | UK | Single center | adult patients | N/A^†^ | 170 | 56 (32.9%) | HNF1A-MODY | 179 | 74 (41.3%) |
| Millan et al. ^7^ | 2021 | N/A | Argentina | Single center | all-age | N/A^†^ | 35 | N/A | HNF1A-MODY | 12 | N/A |
| Szopa et al. ^8^ | 2015 | N/A | Poland | Single center | adult patients | N/A^†^ | 72 | 43 (59.7%) | HNF1A-MODY | 72 | 46 (63.9%) |
| Huang X et al. ^9^ | 2018 | N/A | China | Single center | all-age | N/A^†^ | 11 | 4 (33.3%) | HNF1A-MODY | 12 | 7 (63.6%) |
| Johansen et al. ^10^ | 2005 | 1997-2003 | Denmark | Single center | adult patients | Probands | 8 | 2 (25.0%) | HNF1A-MODY | 28 | 8 (28.6%) |
| Donath et al. ^11^ | 2019 | 2014.01-2017.10 | France | Multicenter | adult patients | Probands | 109 | 32 (29.4%) | HNF1A-MODY | 82 | 25 (30.5%) |
| Campos et al. ^12^ | 2022 | 2011-2020 | Brazil | Single center | all-age | Probands | 82 | 42 (51.2%) | HNF1A-MODY | 21 | 5 (23.8%) |
| Pruhova et al. ^13^ | 2003 | N/A | Czech Republic | Single center | all-age | Probands | 19 | 11 (57.9%) | HNF1A-MODY | 7 | 3 (42.9%) |
| Stankute et al. ^14^ | 2020 | 2013.01-2015.04 | Lithuania – Switzerland | Single center | all-age | Probands | 23 | 9 (39.1%) | HNF1A-MODY | 8 | 2 (25.0%) |
| Barrio et al. ^15^ | 2002 | N/A | Spain | Single center | pediatric patients | Probands^‡^ | 14 | 10 (71.4%) | HNF1A-MODY | 5 | 2 (40.0%) |
| Passanisi et al. ^16^ | 2021 | 2009.01-2021.02 | Southern Italy | Single center | pediatric patients | Probands | 30 | 13 (43.3%) | HNF1A-MODY | 5 | N/A |
| Lorini et al. ^17^ | 2009 | 1992-1999 | Italy | Multicenter | pediatric patients | Probands | 109 | N/A | HNF1A-MODY | 12 | N/A |
| Gökşen et al. ^18^ | 2021 | 2016.03-2017.04 | Turkey | Multicenter | pediatric patients | Probands | 100 | 59 (59.0%) | HNF1A-MODY | 31 | 14 (45.2%) |
| Pihoker et al. ^19^ | 2013 | 2001-2006 | US | Multicenter | pediatric patients | Probands | 14 | 8 (57.1%) | HNF1A-MODY | 26 | 7 (26.9%) |
| Yorifuji et al. ^20^ | 2012 | 2005-2011 | Japan | Single center | pediatric patients | Probands | 18 | 13 (72.2%) | HNF1A-MODY | 11 | 5 (45.5%) |
| Schober et al. ^21^ | 2009 | 1991-2008.09 | Germany and Austria | Multicenter | pediatric patients | Probands | 169 | 93 (55.0%) | HNF1A-MODY | 84 | 25 (29.8%) |
| Fu et al. ^22^ | 2021 | 2014.01-2018.12 | China | Single center | all-age | Probands | 106 | 44 (41.5%) | HNF1A-MODY | 34 | 14 (41.2%) |
| Campos et al. ^12^ | 2022 | 2011-2021 | Brazil | Single center | all-age | Relatives^†^ | 108 | 55 (50.9%) | HNF1A-MODY | 17 | 7 (41.2%) |
| Owen et al. ^5^ | 2010 | N/A | UK | Multicenter | adult patients | All Families | 24 | 10 (41.7%) | Type 2 diabetes | 240 | 132 (55.0%) |
| Franco et al. ^23^ | 2021 | N/A | Brazil | Single center | adult patients | All Families | 29 | 6 (20.7%) | Type 2 diabetes | 24 | 9 (37.5%) |
|  |  |  |  |  |  |  |  |  |  |  |  |
| Dusatkova et al. ^24^ | 2022 | 1999-2014 | Czech Republic | Single center | adult patients | All Families | 162 | 56 (34.6%) | Type 2 diabetes | 84 | 54 (64.3%) |
| McDonald et al. ^25^ | 2011 | N/A | UK | Single center | adult patients | All Families | 245 | 86 (35.1%) | Type 2 diabetes | 157 | 88 (56.1%) |
| Ma Y et al. ^26^ | 2019 | 2012.03-2013.05 | China | Single center | adult patients | All Families | 9 | 4 (44.4%) | Type 2 diabetes | 330 | 184 (55.8%) |
| Sagen et al. ^27^ | 2008 | 1997-N/A | Norway | Single center | all-age | All Families | 56 | N/A | Type 2 diabetes | 99 | N/A |
| Elias-Assad et al. ^28^ | 2021 | N/A | Israel | Single center | all-age | All Families | 42 | 16 (38.1%) | Type 2 diabetes | 39 | 16 (41.0%) |
| Velho et al. ^29^ | 1996 | N/A | France | Single center | all-age | All Families | 195 | 98 (50.3%) | Type 2 diabetes | 393 | 192 (48.9%) |
| Szopa et al. ^8^ | 2015 | N/A | Poland | Single center | adult patients | N/A^†^ | 72 | 43 (59.7%) | Type 2 diabetes | 70 | 31 (44.3%) |
| Fu et al. ^22^ | 2021 | 2014.01-2018.12 | China | Single center | all-age | Probands | 106 | 44 (41.5%) | Type 2 diabetes | 82 | 37 (45.1%) |
| Arslanian et al. ^30^ | 2020 | 2004.07-2009.02 | US | Multicenter | pediatric patients | Probands | 7 | 3 (42.9%) | Type 2 diabetes | 426 | 149 (35.0%) |
| Pacaud et al. ^31^ | 2016 | 2006-2016.02 | Multi-region | Multicenter | pediatric patients | Probands | 188 | 54 (28.7%) | Type 2 diabetes | 347 | 139 (40.1%) |
| Agladioglu et al. ^32^ | 2016 | N/A | Turkey | Single center | pediatric patients | Probands | 18 | 11 (61.1%) | Type 2 diabetes | 15 | 8 (53.3%) |
| Schober et al. ^21^ | 2009 | 1991-2008.09 | Germany and Austria | Multicenter | pediatric patients | Probands | 169 | 93 (55.0%) | Type 2 diabetes | 562 | 202 (35.9%) |

N/A: not provided in the original article

†: These studies were categorized to “All Families” group when analyzing

‡: The study done by Barrio et al. ^15^ involved pediatric probands and pediatric relatives.

# Forest Plots for Baseline Differences between GCK-MODY and HNF1A-MODY/Type 2 Diabetes

## Figure S2. The forest plot for age at diagnosis in HNF1A-MODY studies


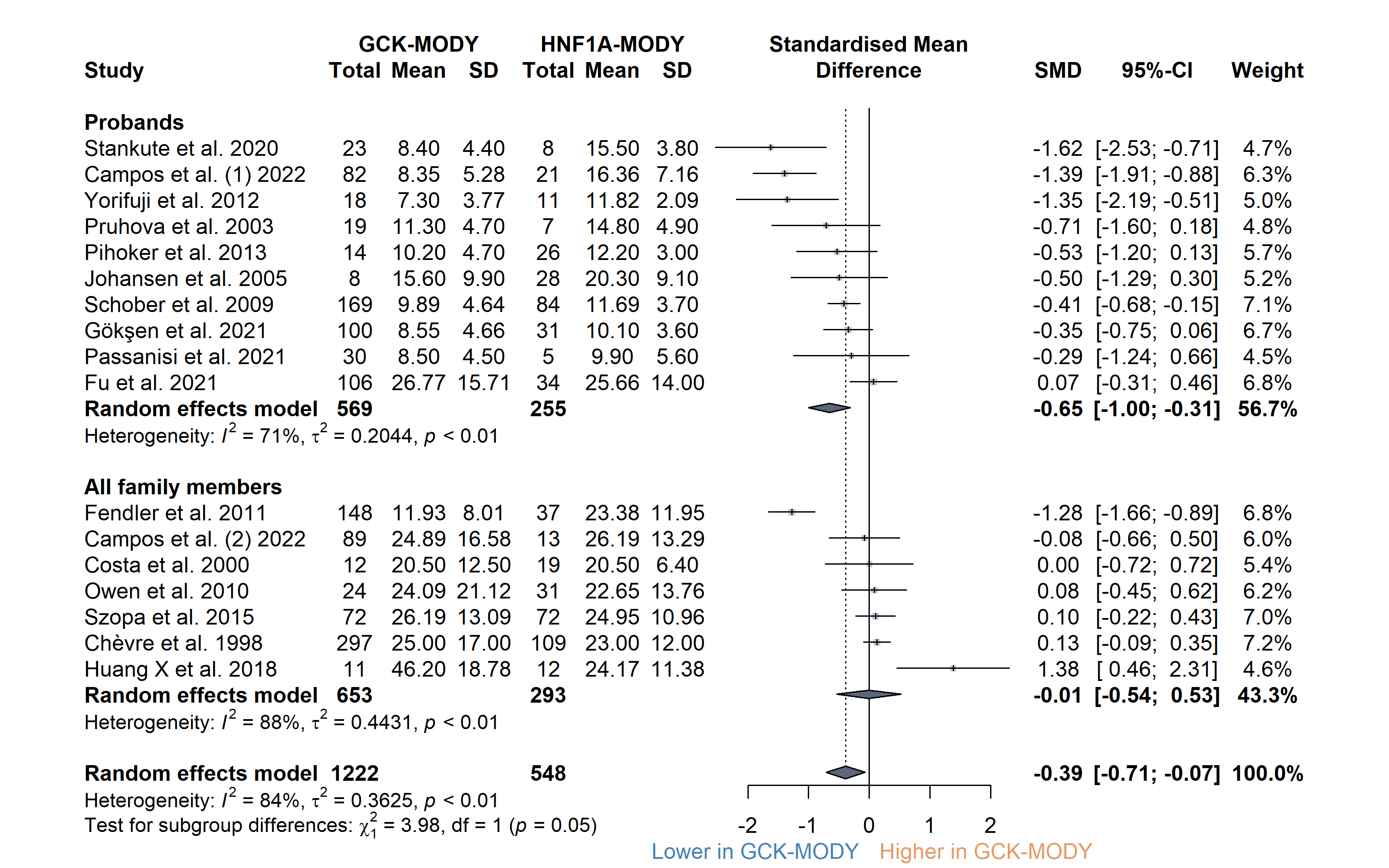


## Figure S3. The forest plot for age at diagnosis in type 2 diabetes studies


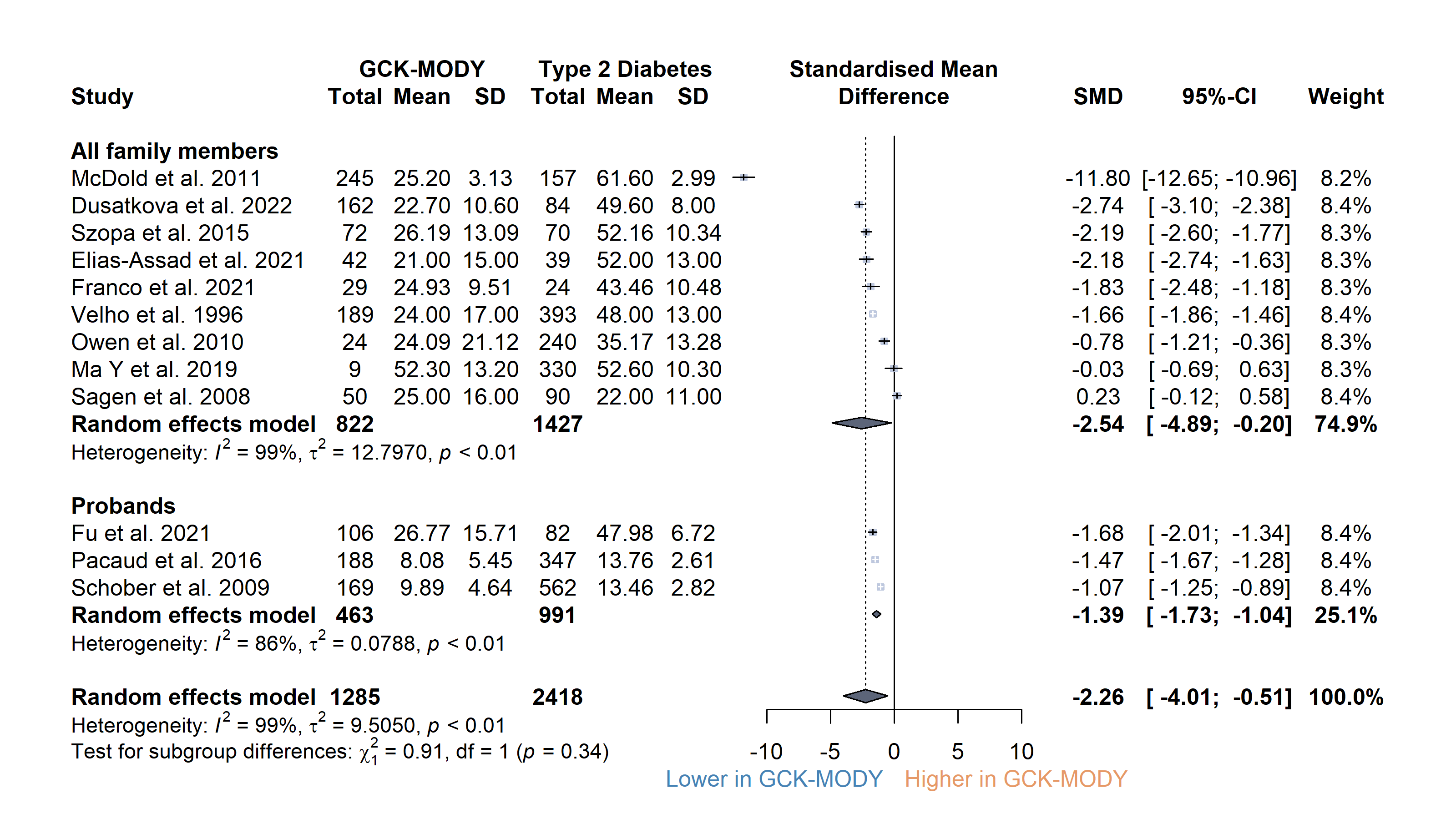


## Figure S4. The forest plot for birthweight in HNF1A-MODY studies


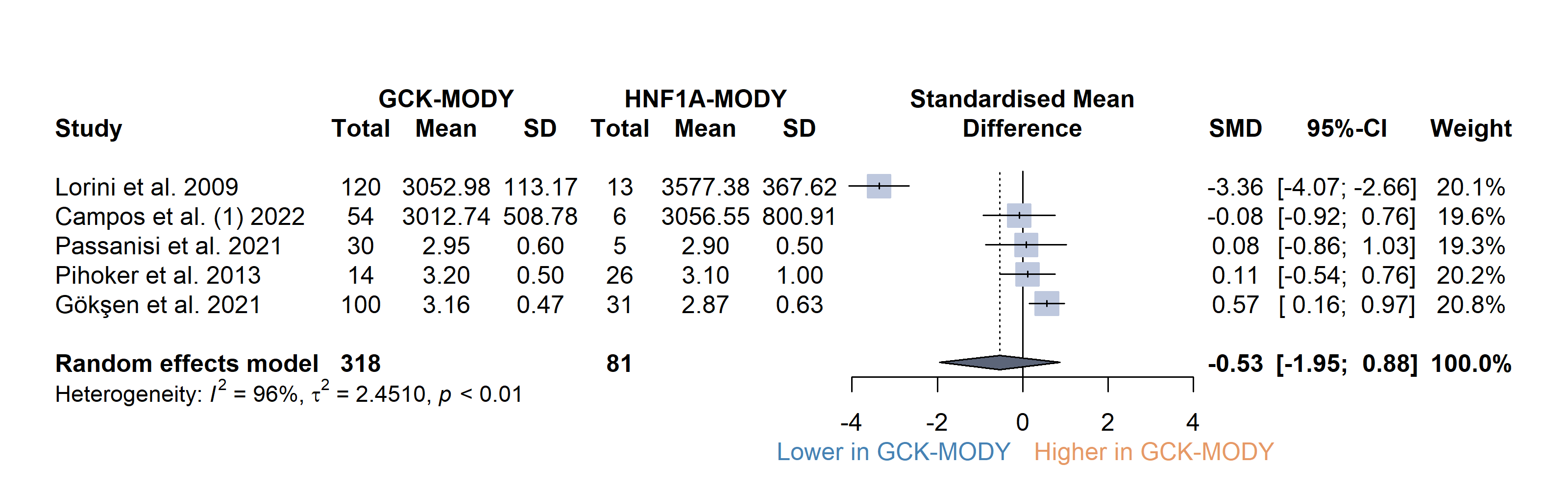


## Figure S5. The forest plot for BMI (SDS) in HNF1A-MODY studies


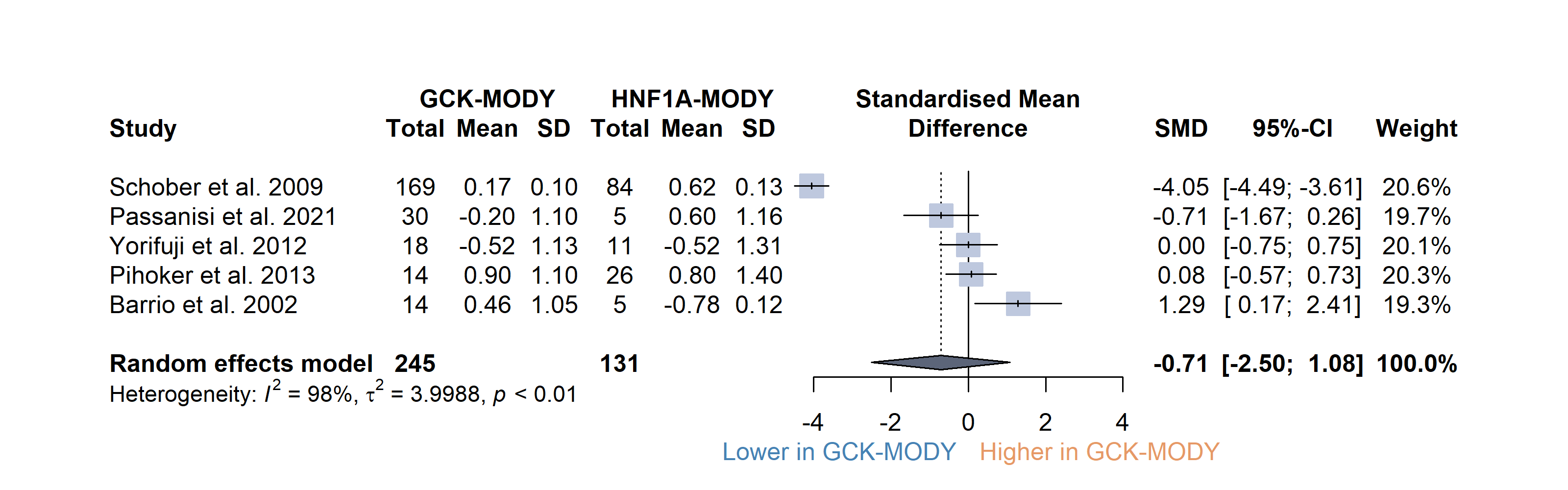


## Figure S6. The forest plot for BMI (SDS) in type 2 diabetes studies


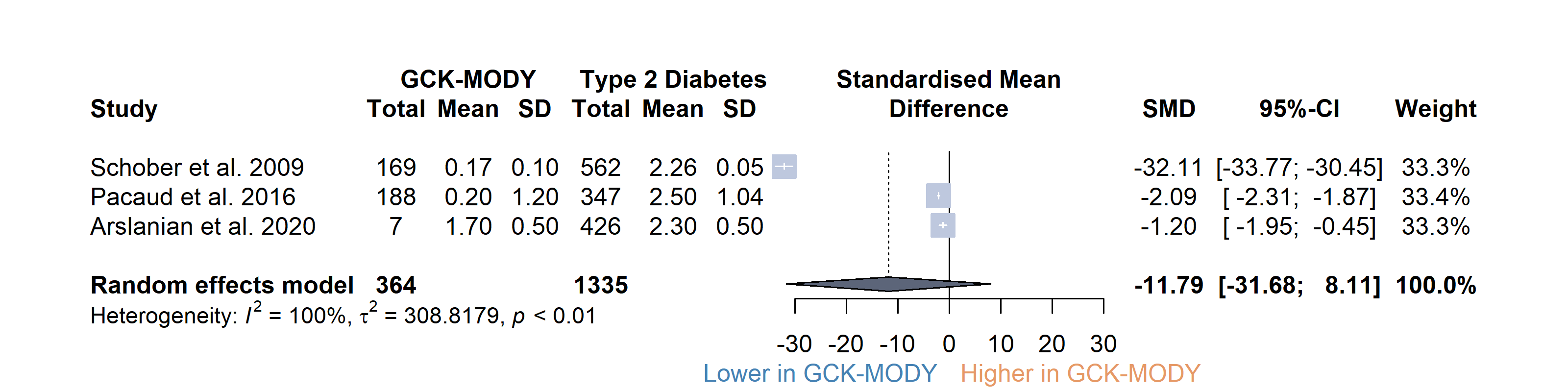


## Figure S7. The forest plot for BMI (kg/m^2^) in HNF1A-MODY studies


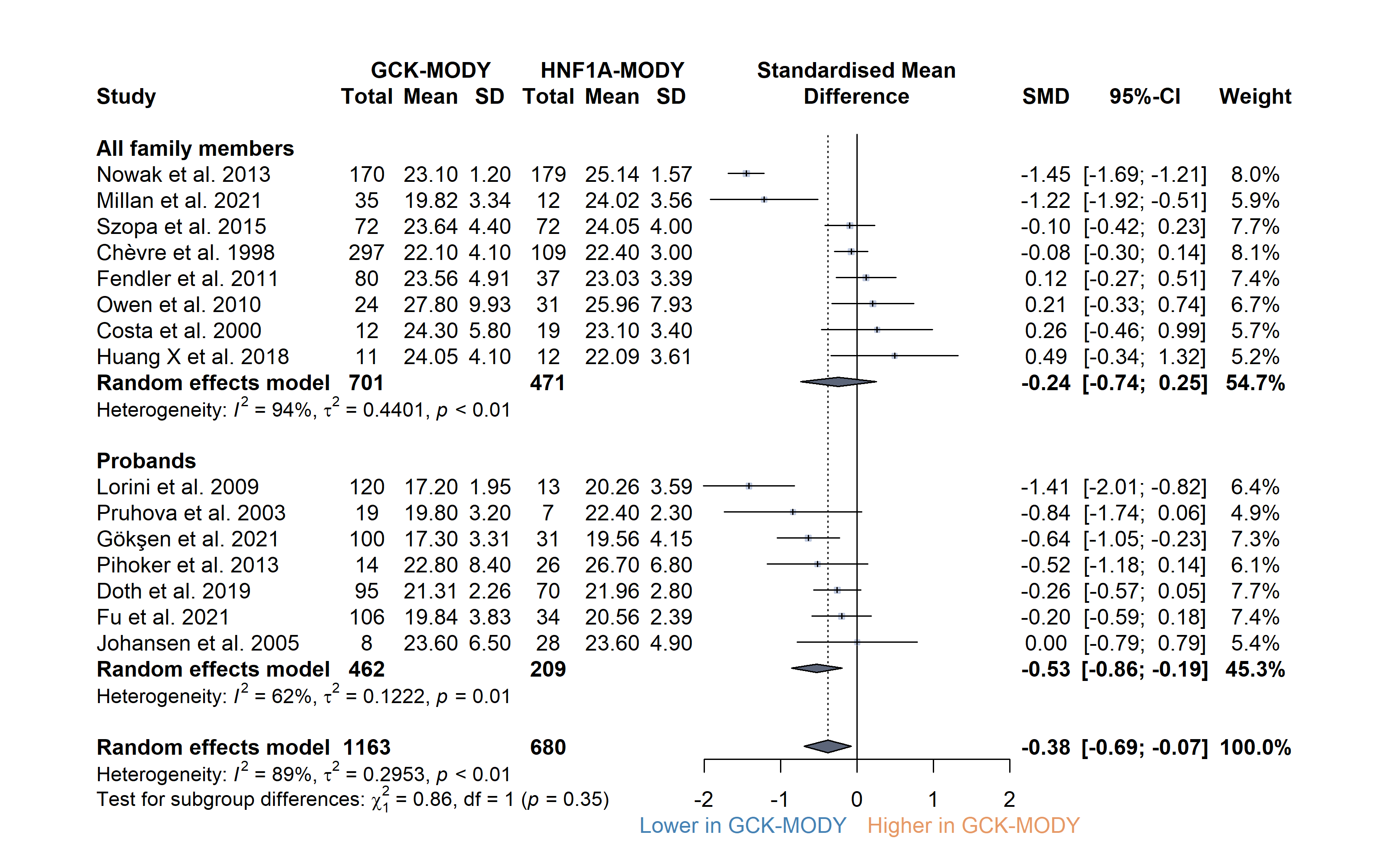


## Figure S8. The forest plot for BMI (kg/m^2^) in type 2 diabetes studies


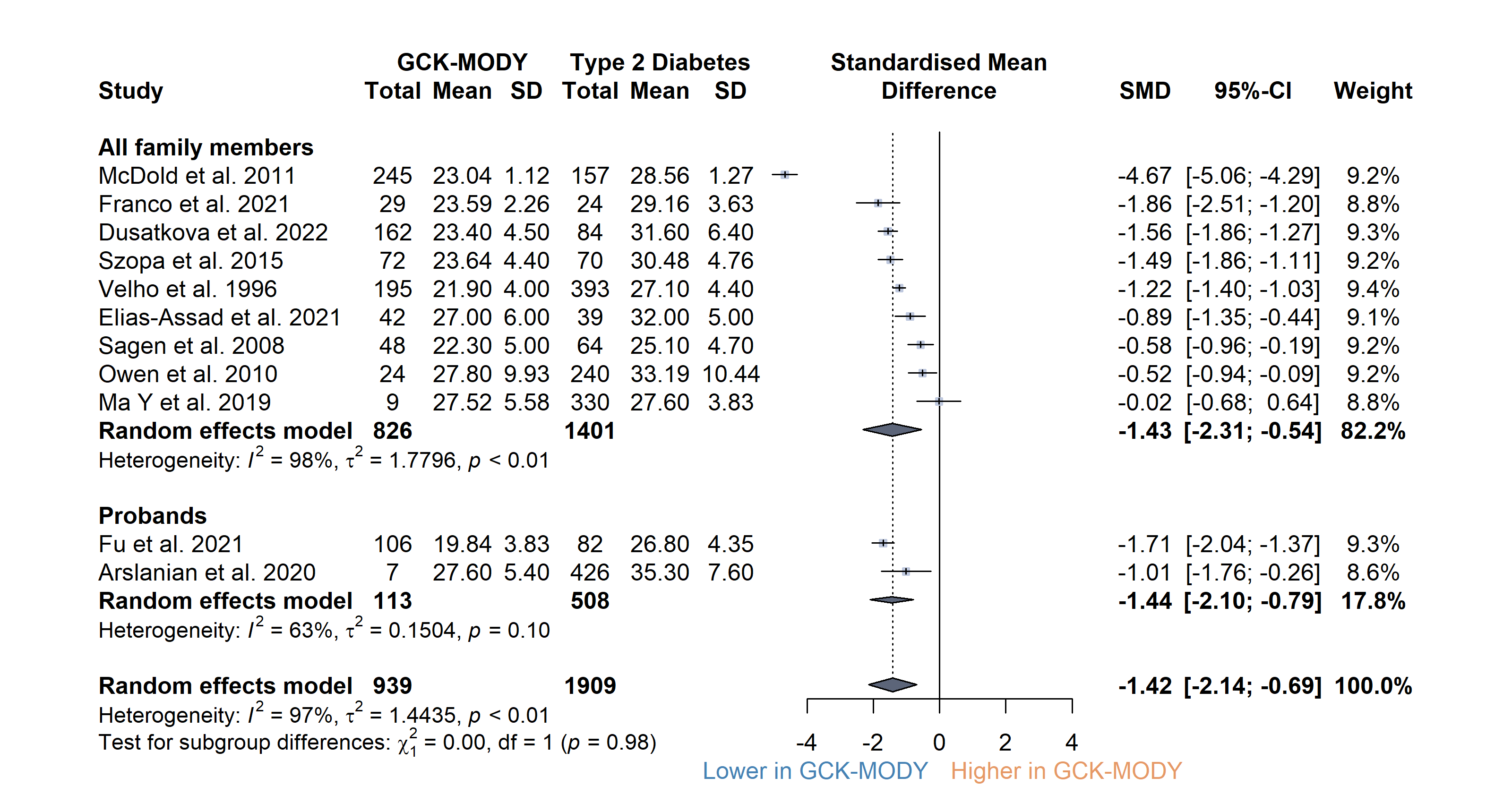


## Figure S9. The forest plot for hsCRP in HNF1A-MODY studies


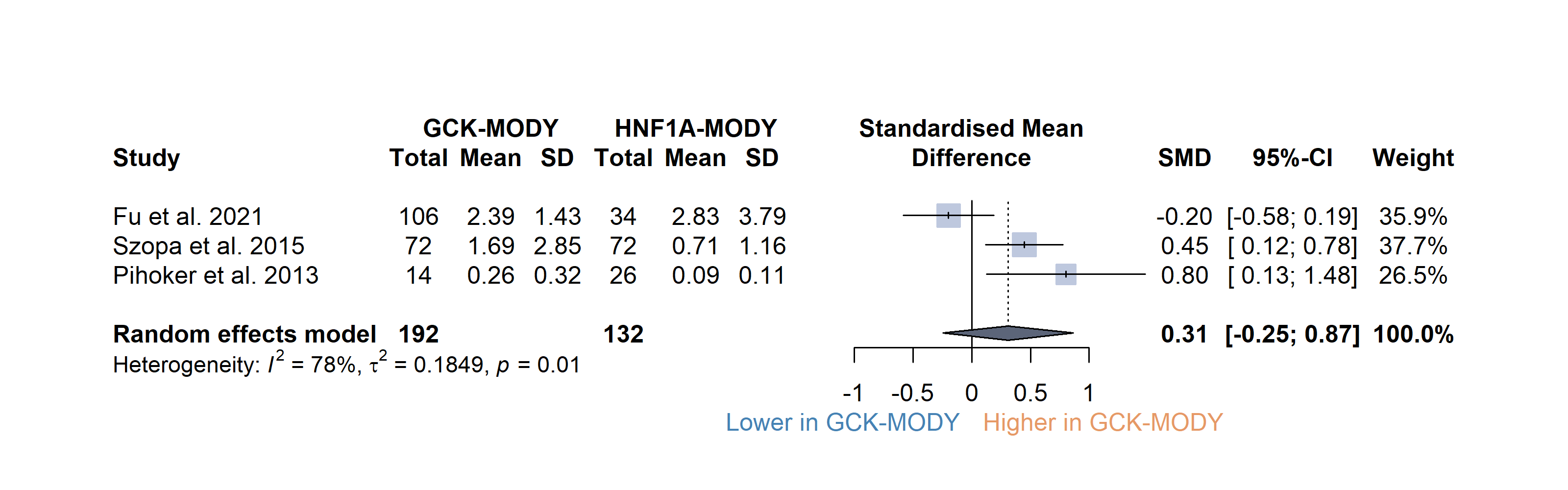


## Figure S10. The forest plot for hsCRP in type 2 diabetes studies


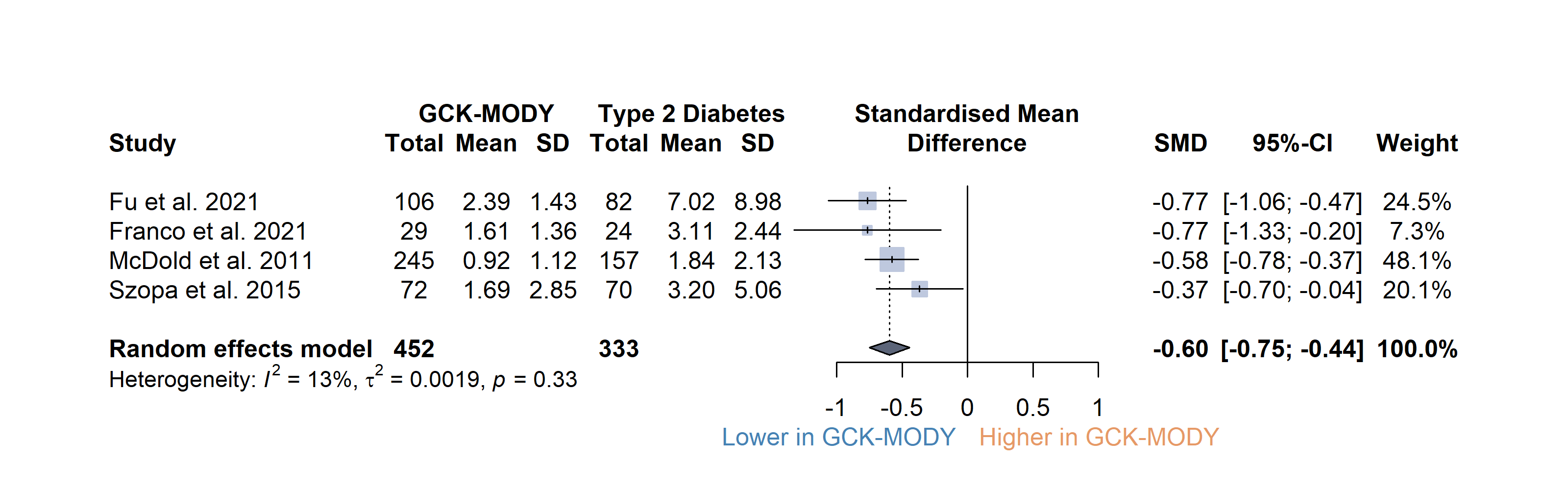


# Forest Plots for Glucose Metabolism Differences between GCK-MODY and HNF1A-MODY/Type 2 Diabetes

## Figure S11. The forest plot for HbA1c in HNF1A-MODY studies


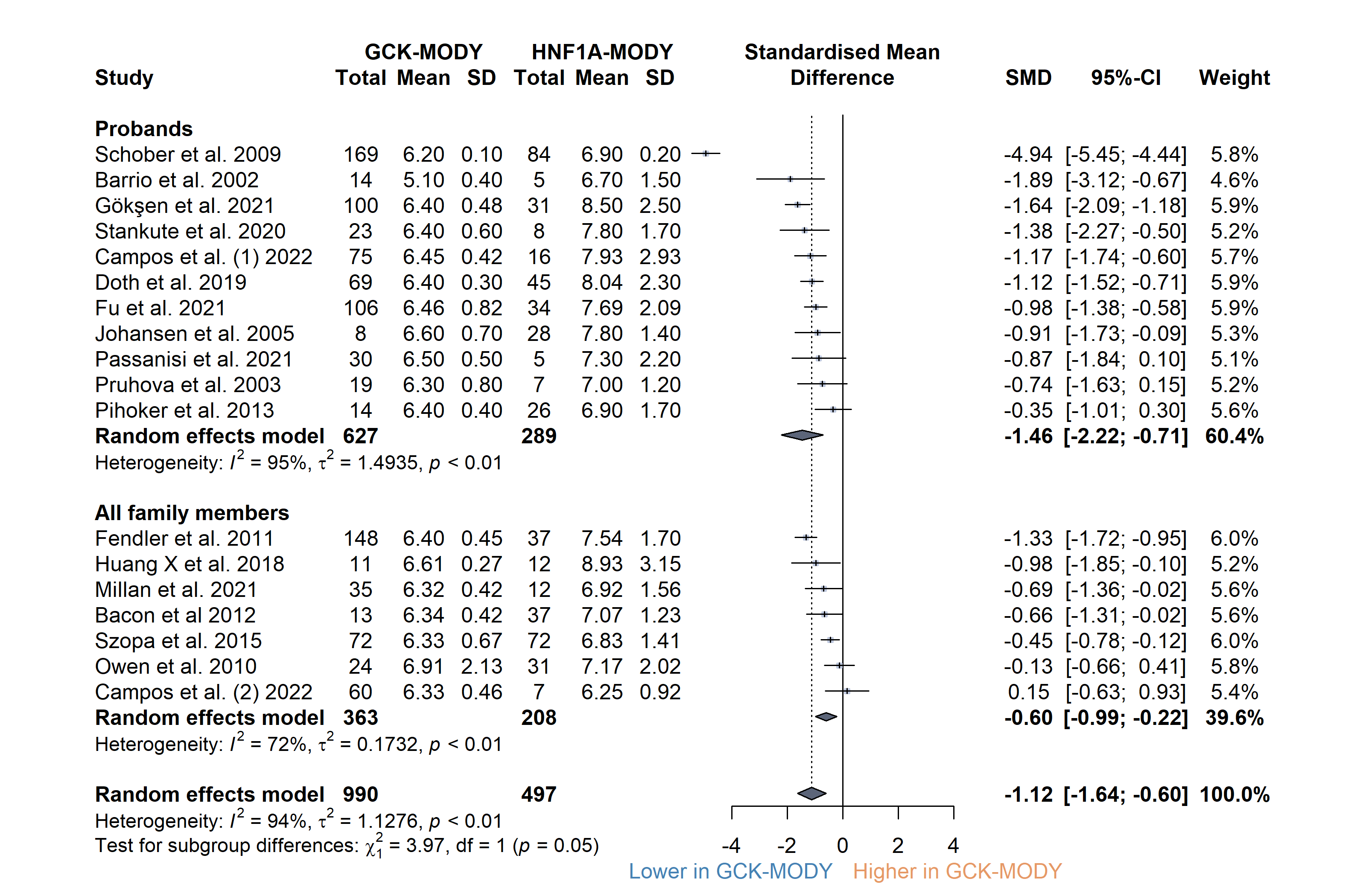


## Figure S12. The forest plot for HbA1c in type 2 diabetes studies


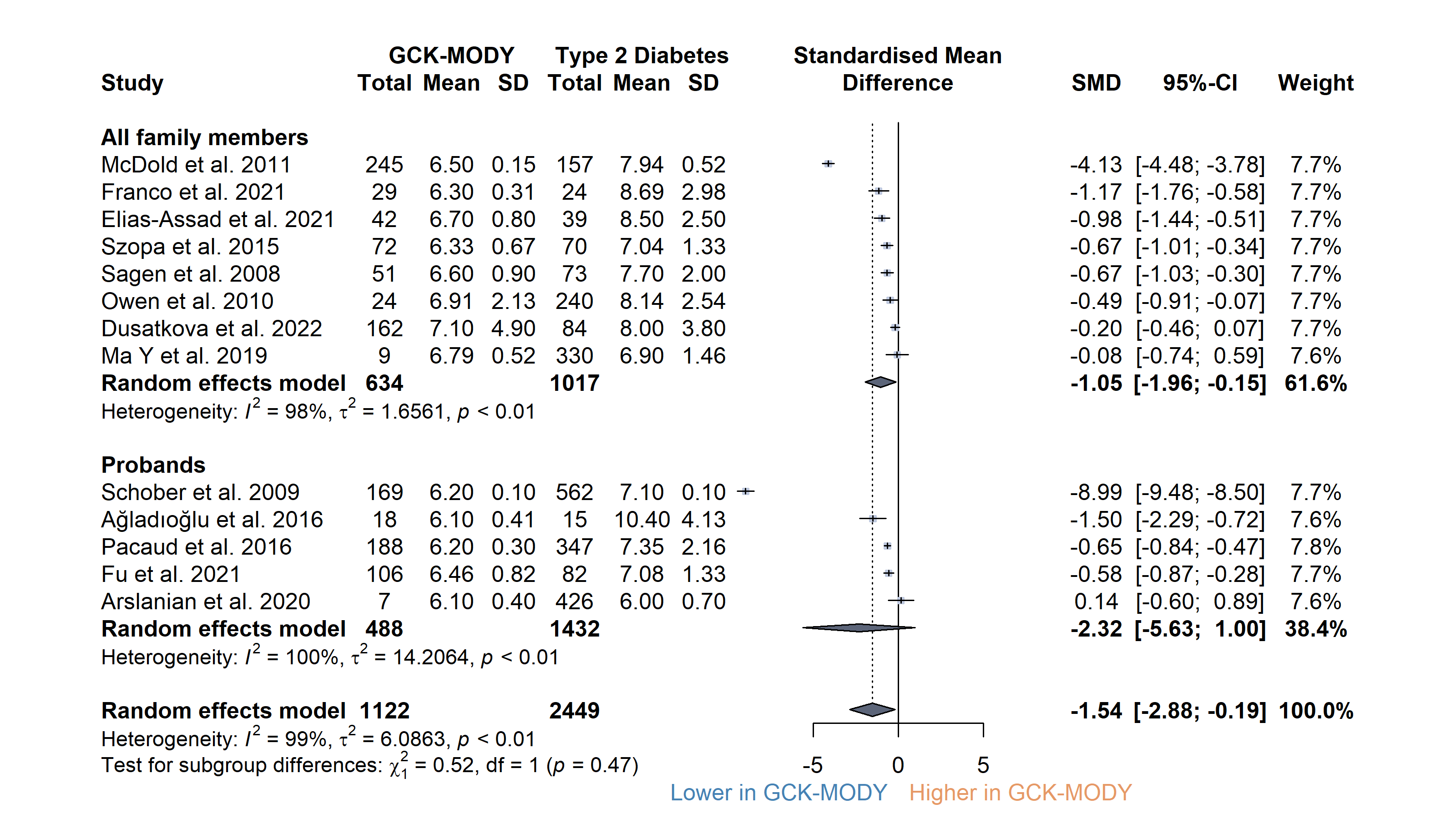


## Figure S13. The forest plot for fasting blood glucose in HNF1A-MODY studies


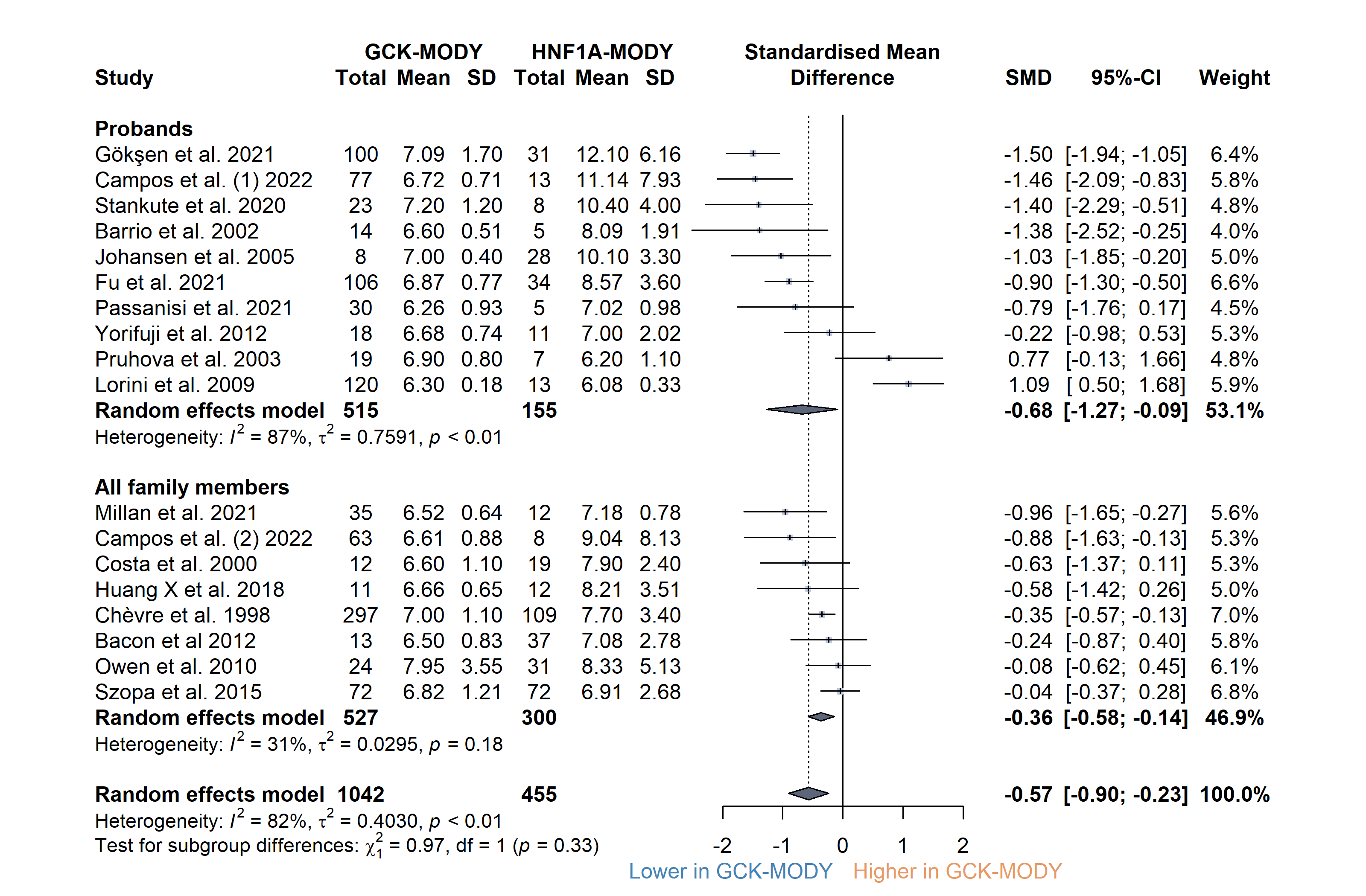


## Figure S14. The forest plot for fasting blood glucose in type 2 diabetes studies


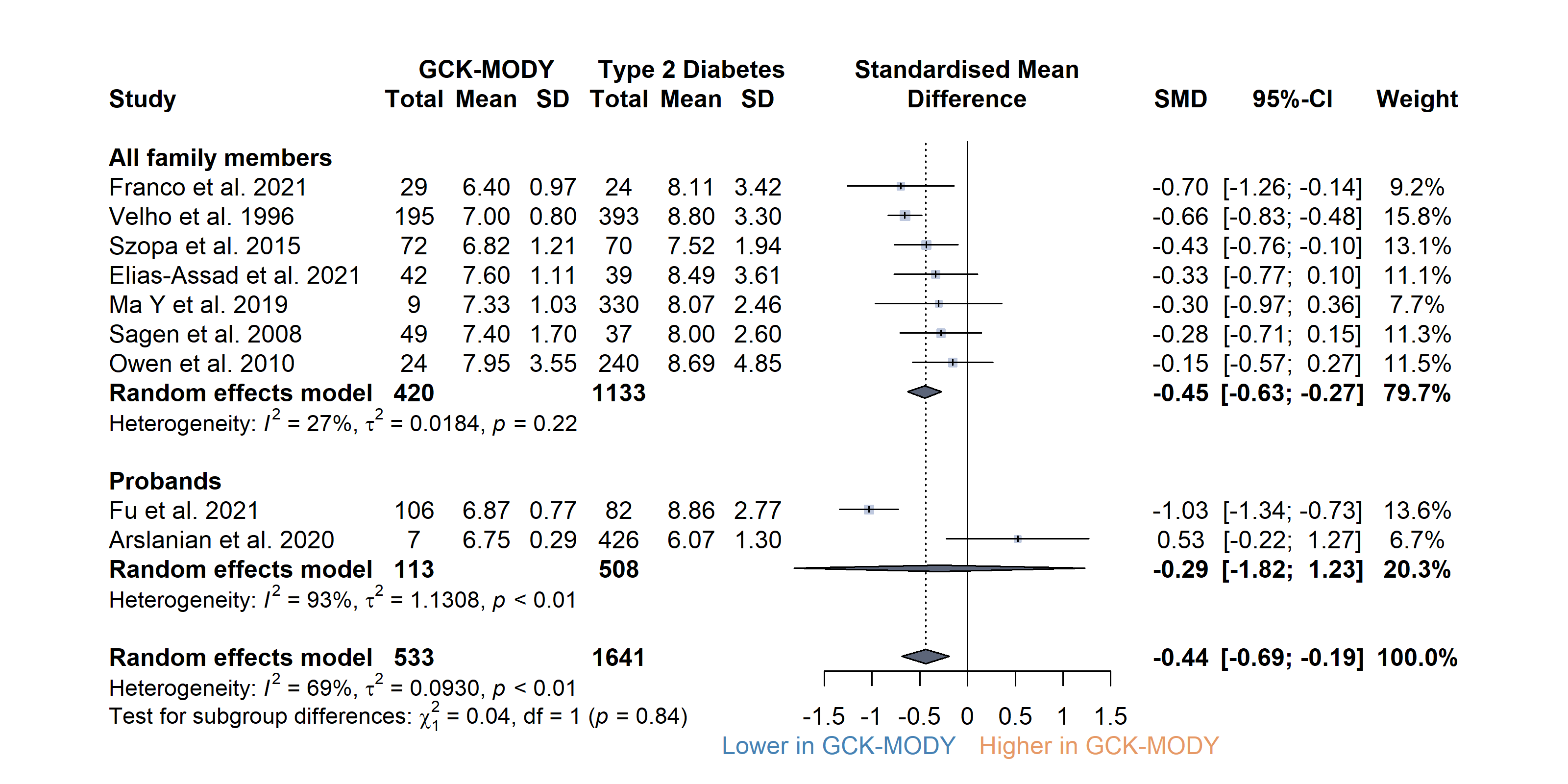


## Figure S15. The forest plot for 2-hour postprandial glucose in HNF1A-MODY studies


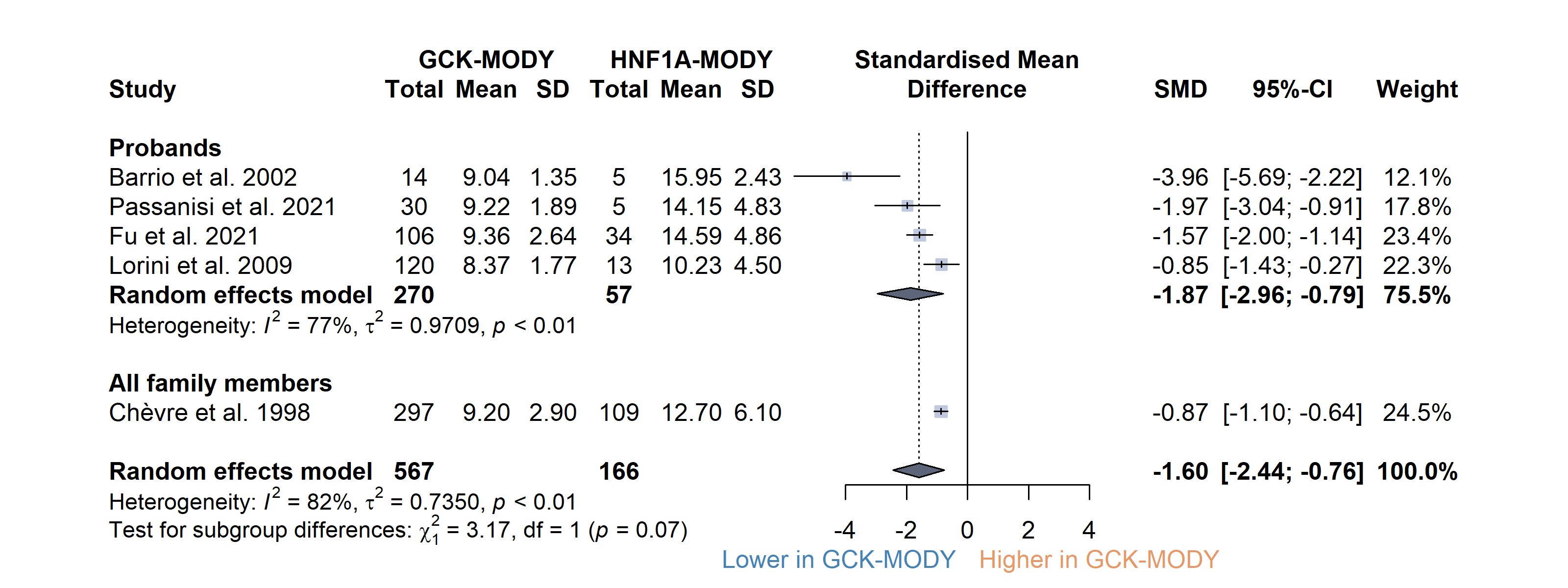


## Figure S16. The forest plot for 2-hour postprandial glucose in type 2 diabetes studies


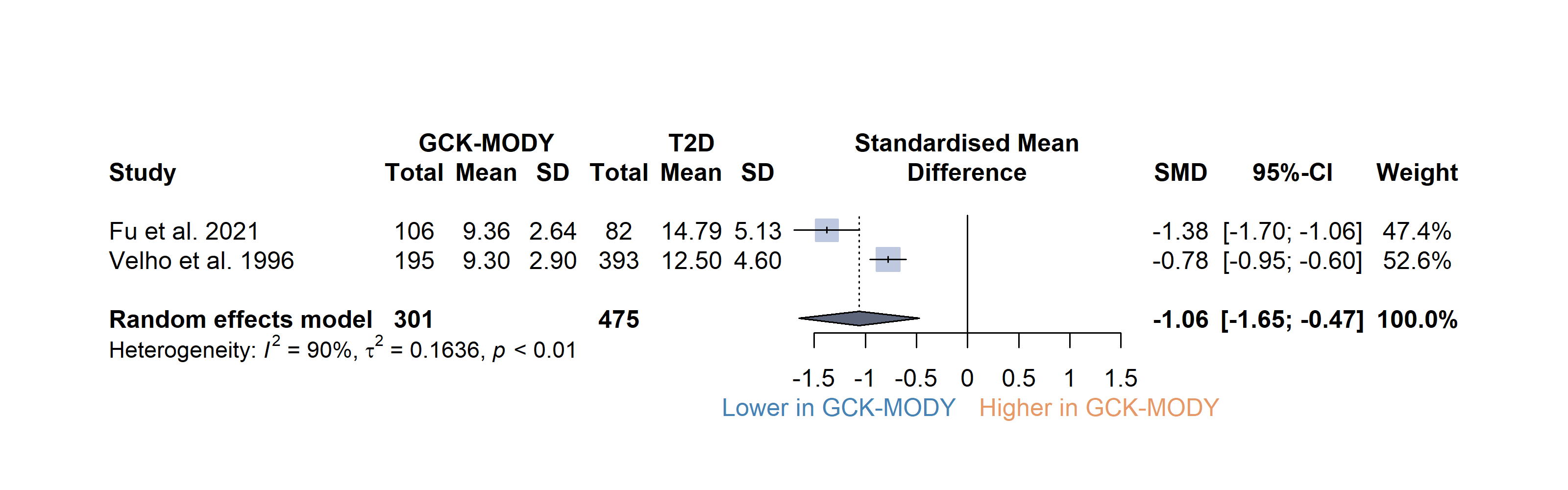


## Figure S17. The forest plot for change in 2-hour postprandial glucose in HNF1A-MODY studies


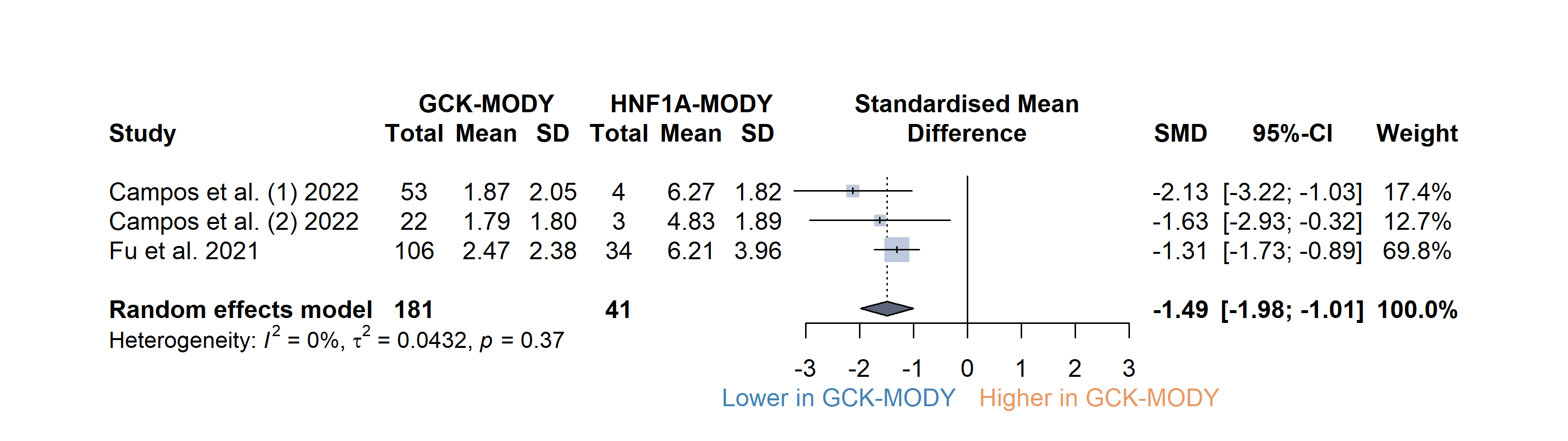


## Figure S18. The forest plot for fasting C peptide in HNF1A-MODY studies


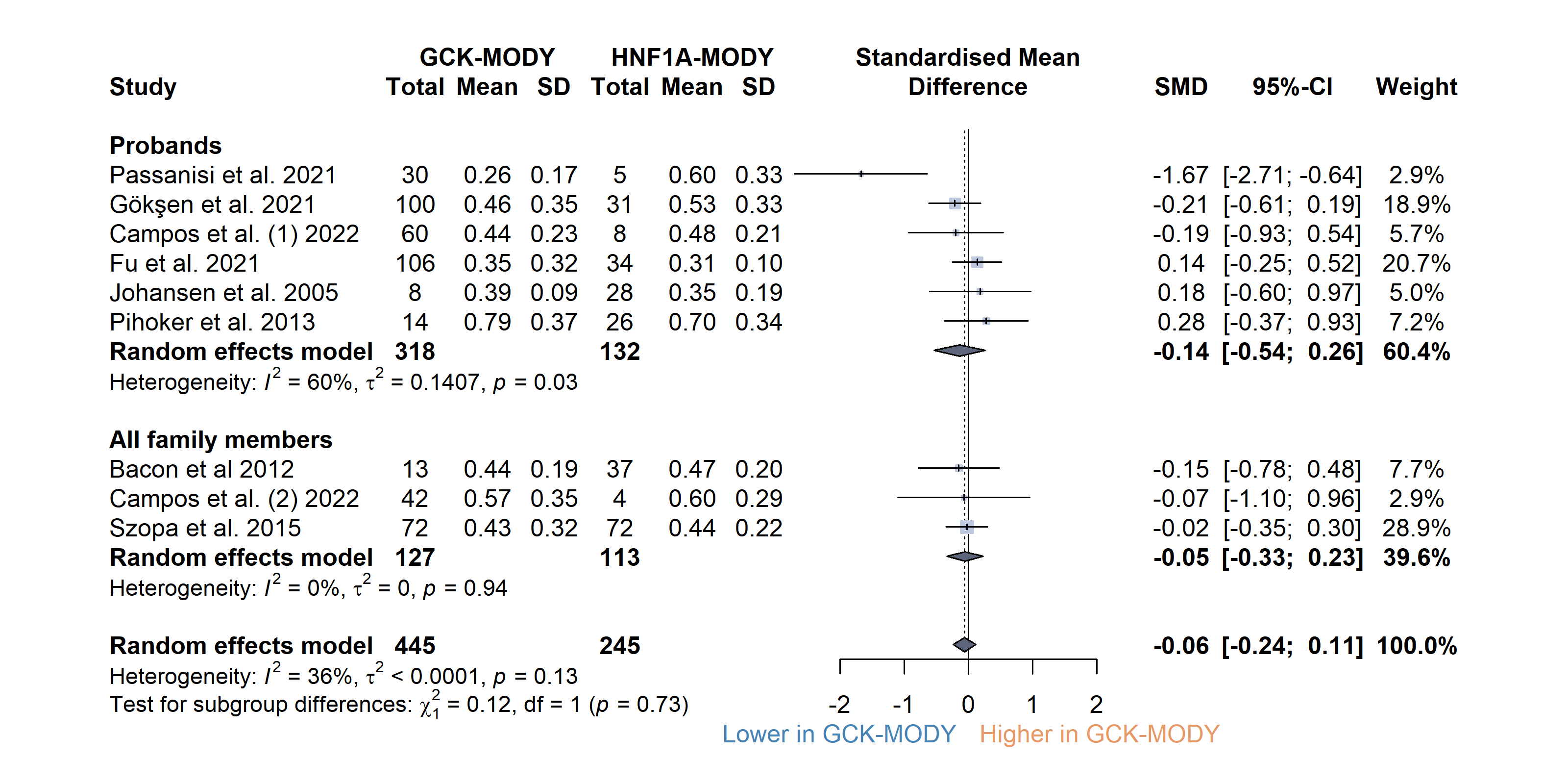


## Figure S19. The forest plot for fasting C peptide in type 2 diabetes studies


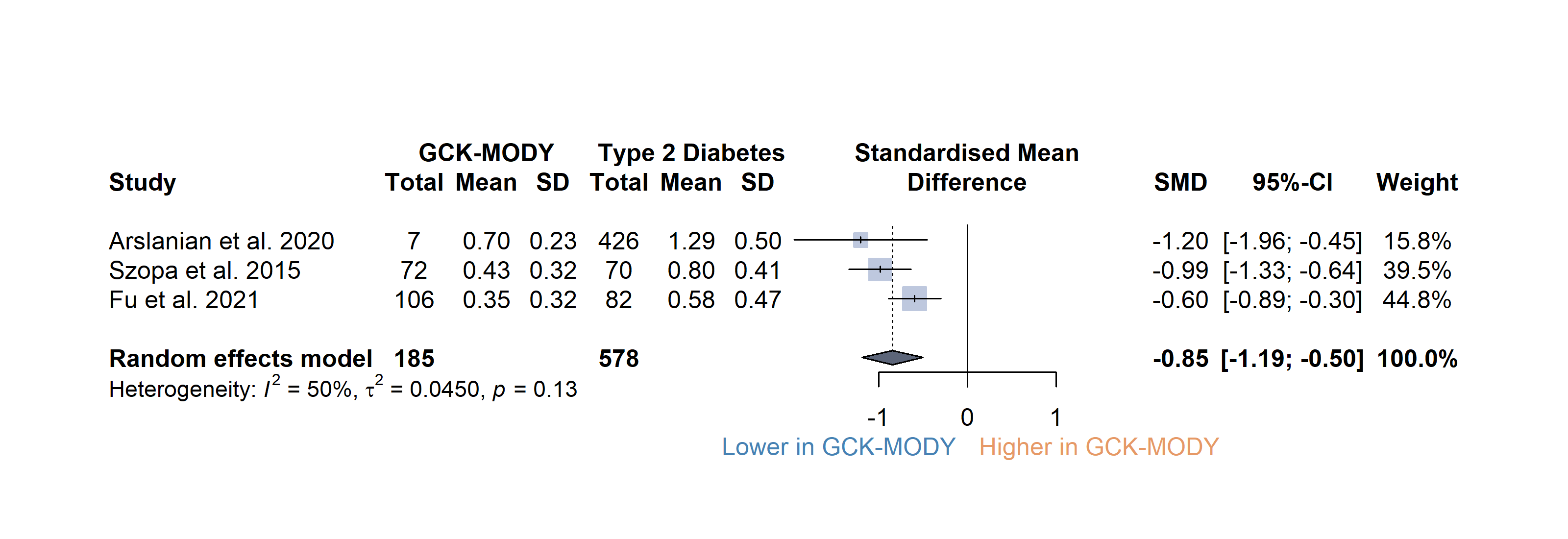


## Figure S20. The forest plot for fasting insulin concentration in HNF1A-MODY studies


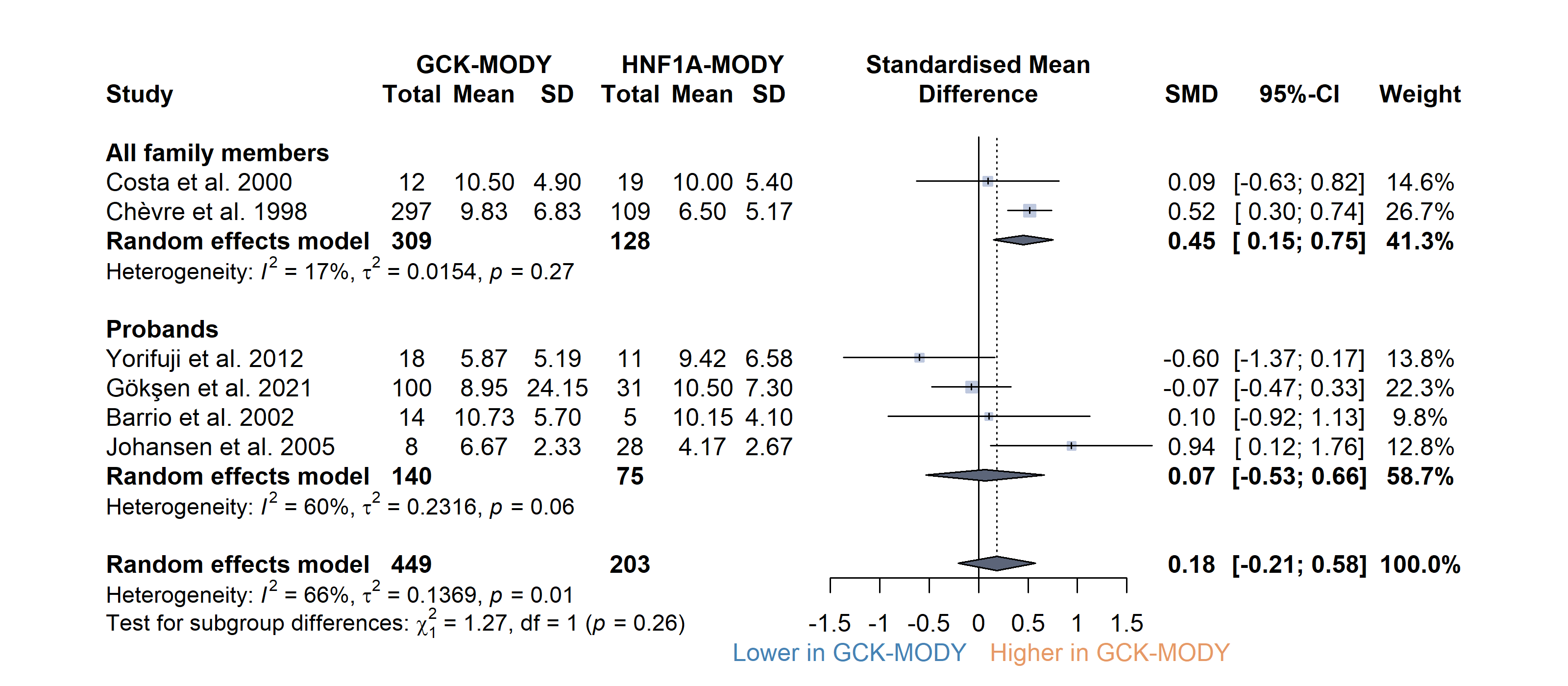


## Figure S21. The forest plot for fasting insulin concentration in type 2 diabetes studies


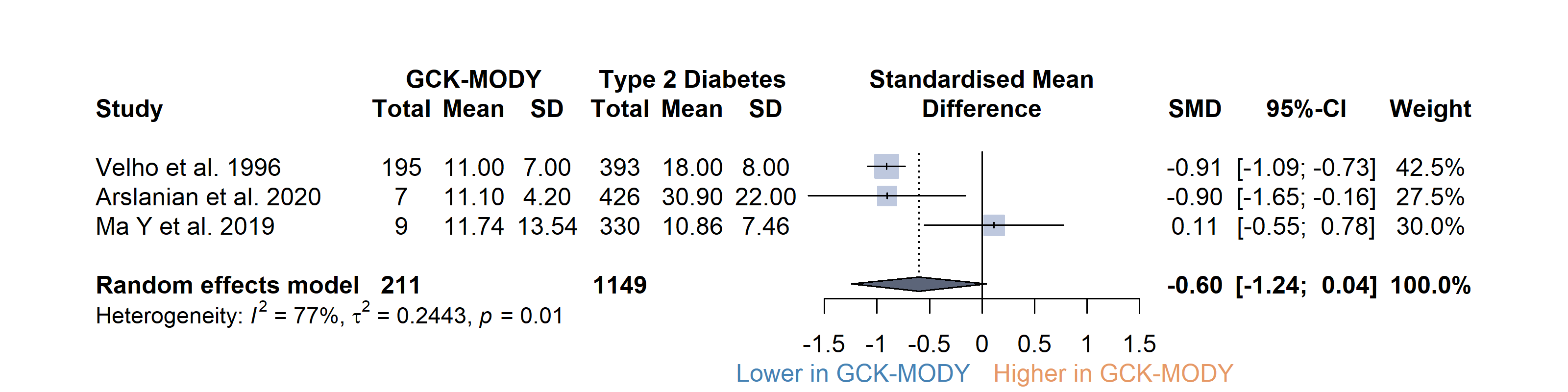


## Figure S22. The forest plot for 2-hour insulin concentration in HNF1A-MODY studies


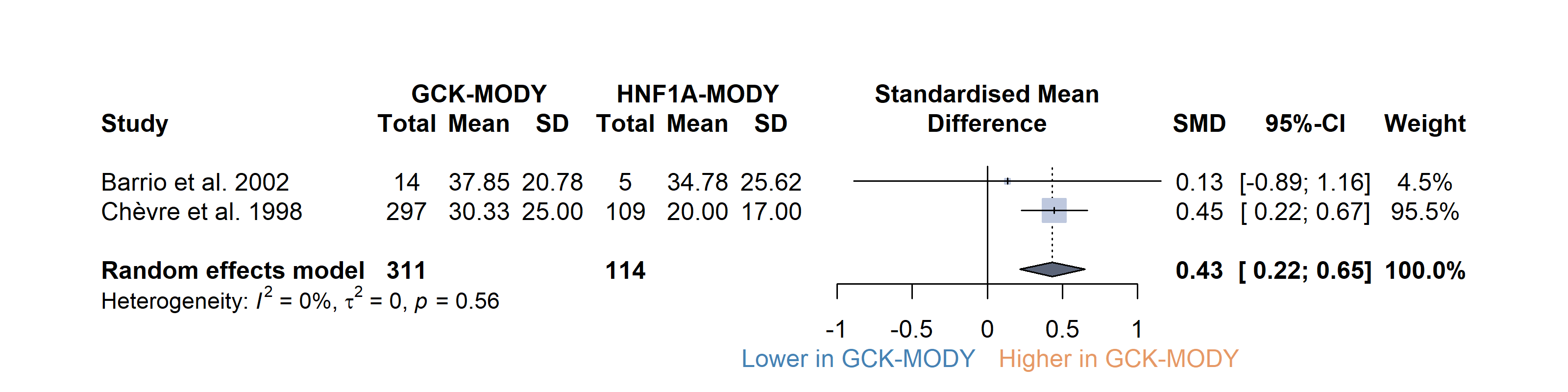


# Forest Plots for Lipid Metabolism Differences between GCK-MODY and HNF1A-MODY/Type 2 Diabetes

## Figure S23. The forest plot for TC in HNF1A-MODY studies


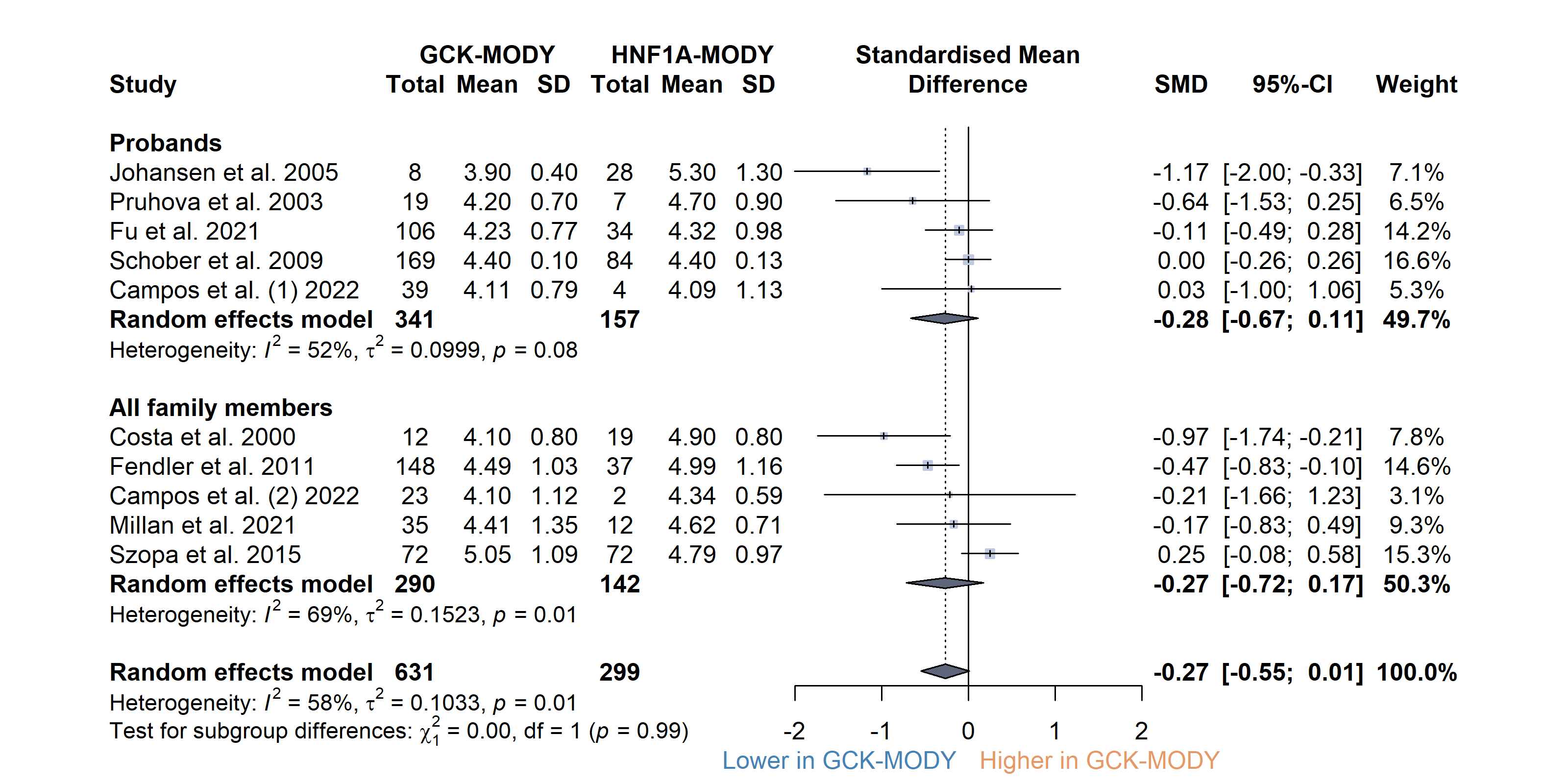


## Figure S24. The forest plot for TC in type 2 diabetes studies


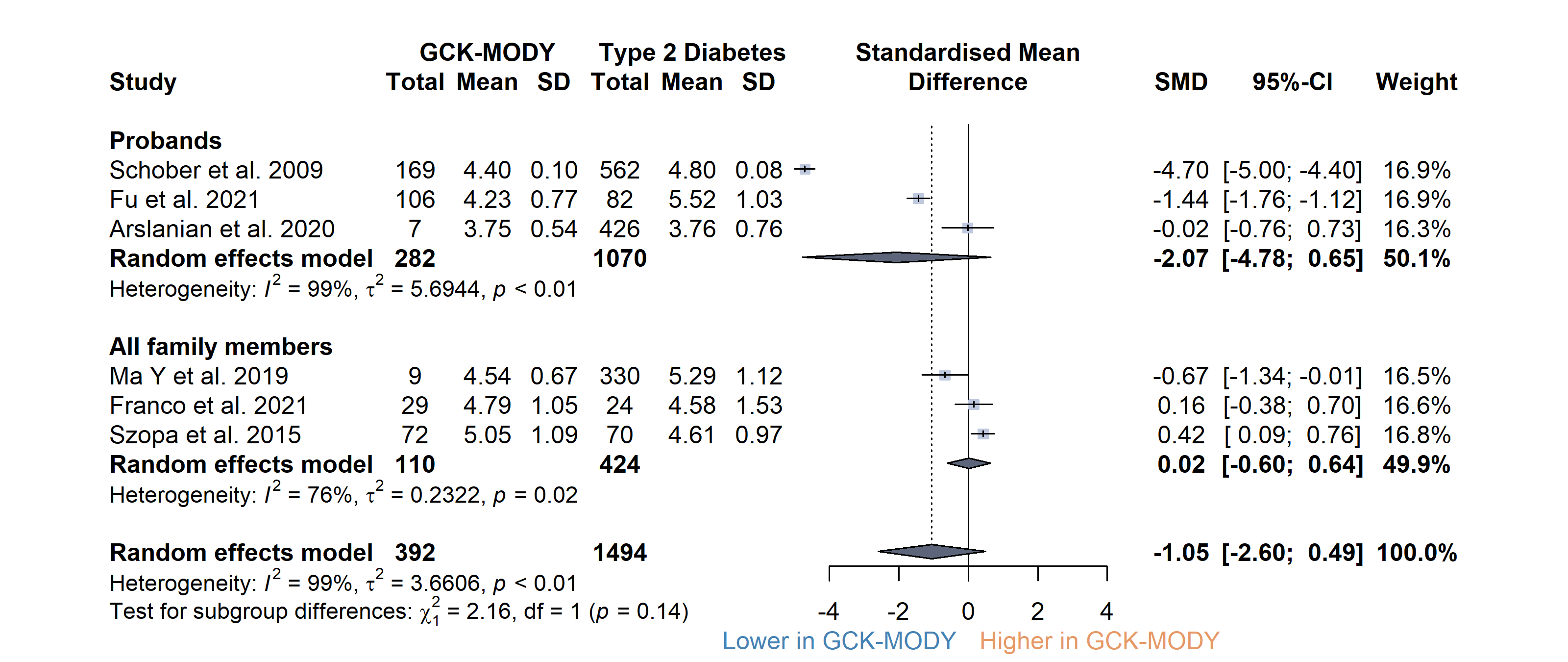


## Figure S25. The forest plot for HDL in HNF1A-MODY studies


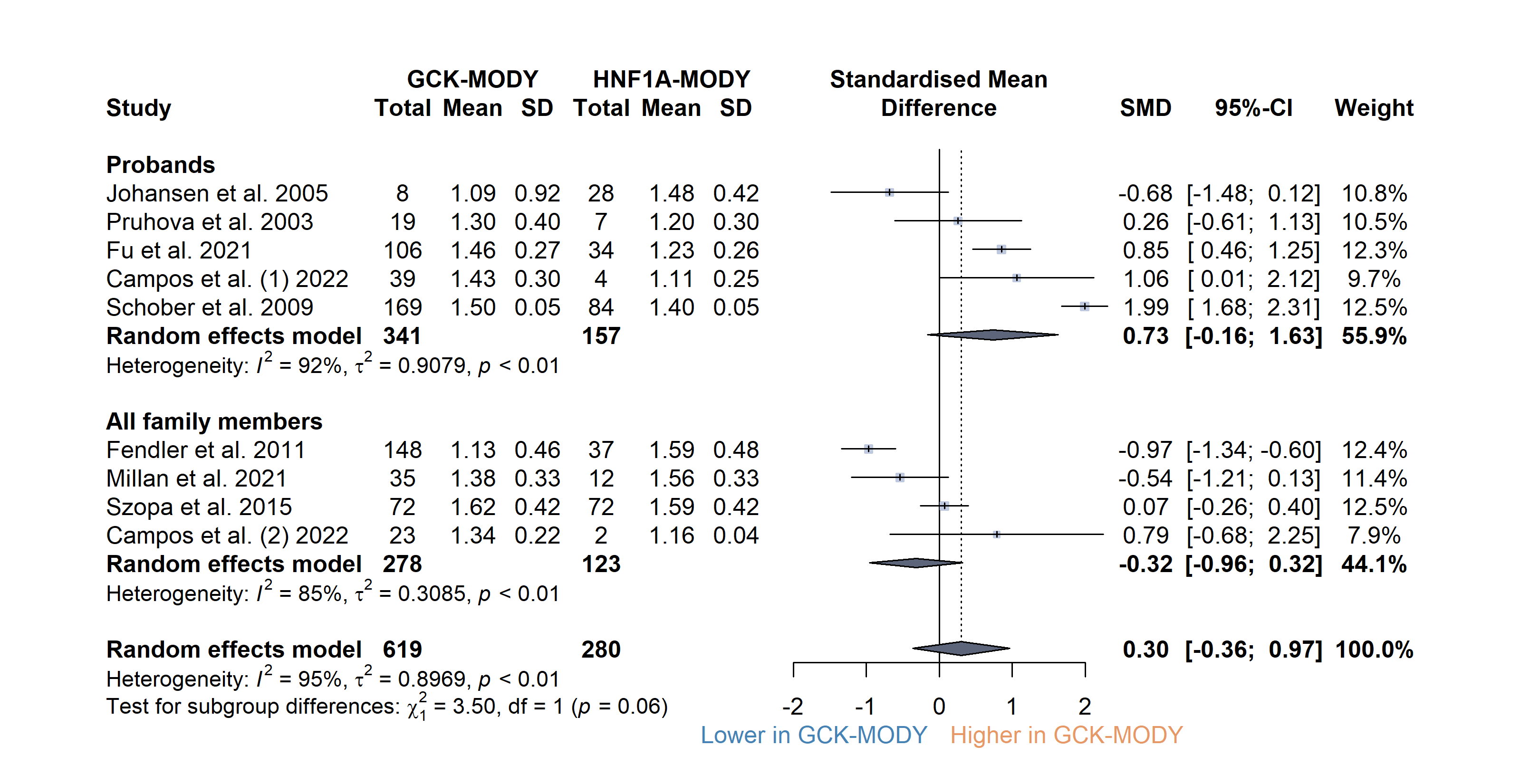


## Figure S26. The forest plot for HDL in type 2 diabetes studies


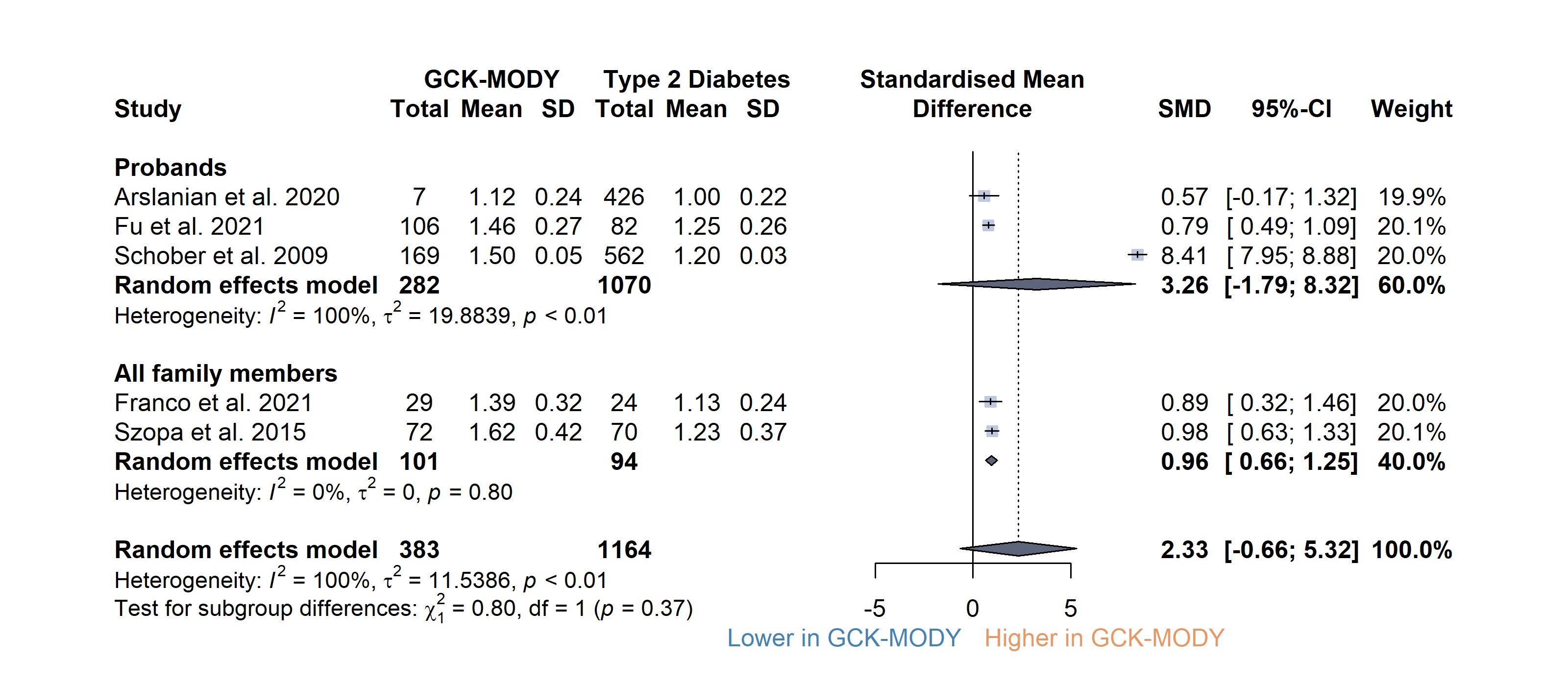


## Figure S27. The forest plot for LDL in HNF1A-MODY studies


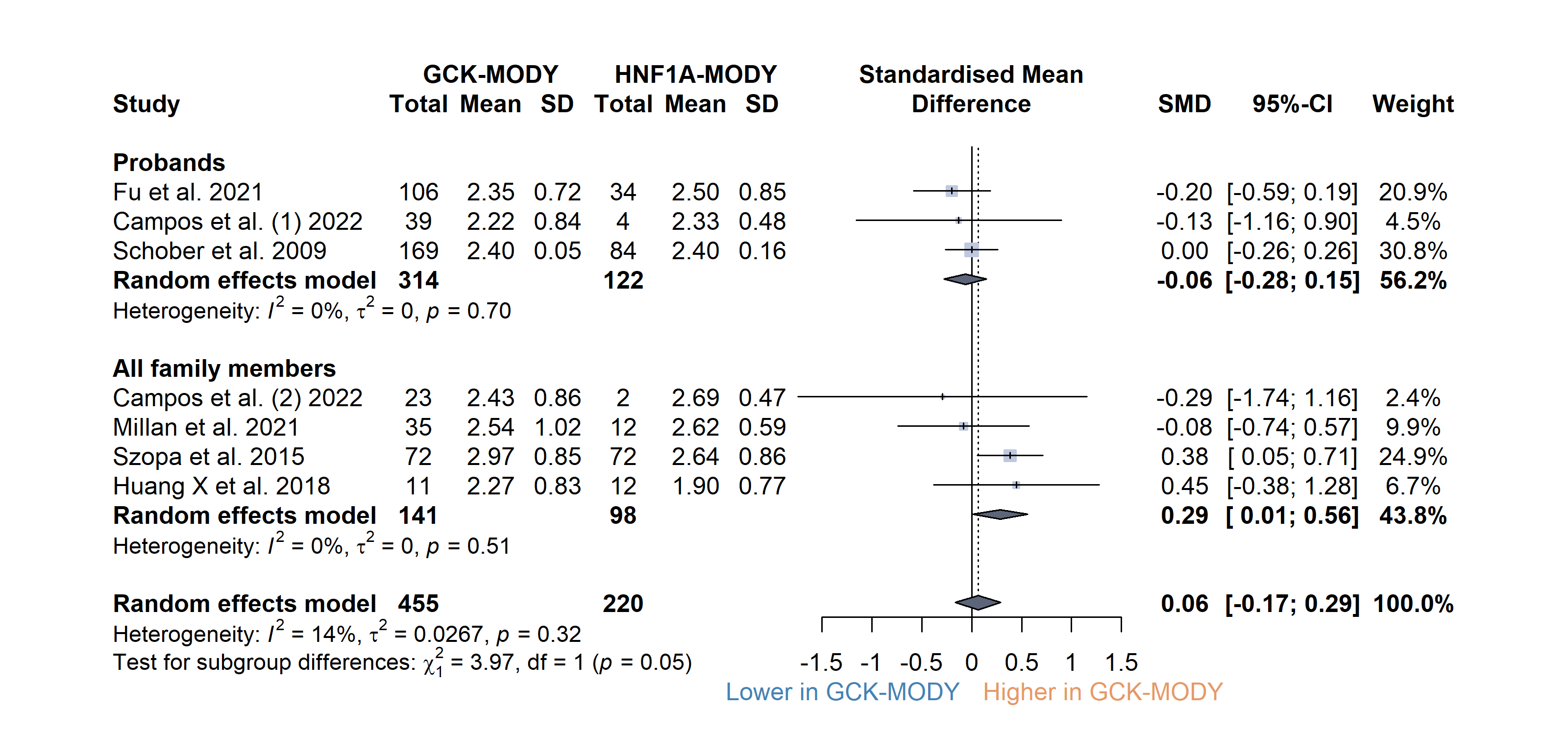


## Figure S28. The forest plot for LDL in type 2 diabetes studies


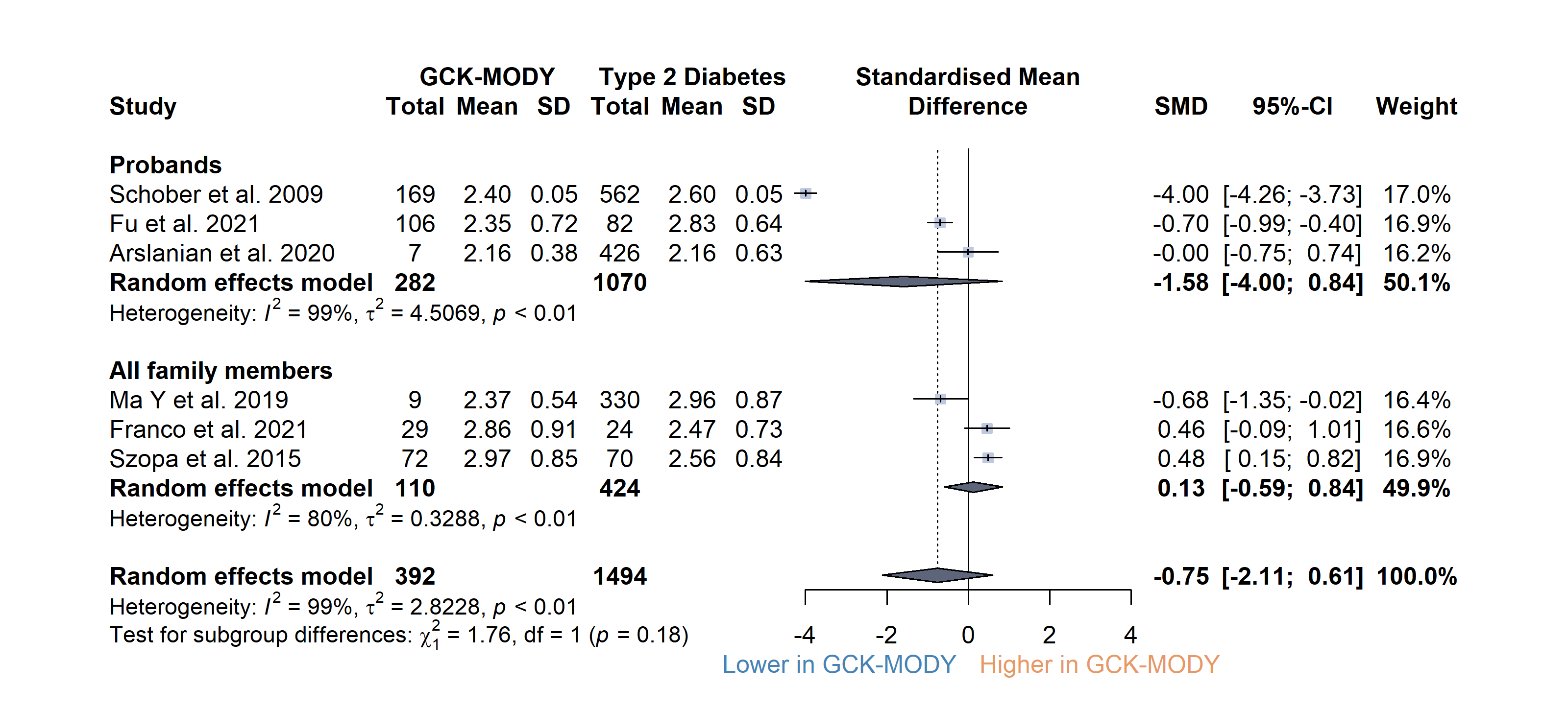


## Figure S29. The forest plot for TG in HNF1A-MODY studies


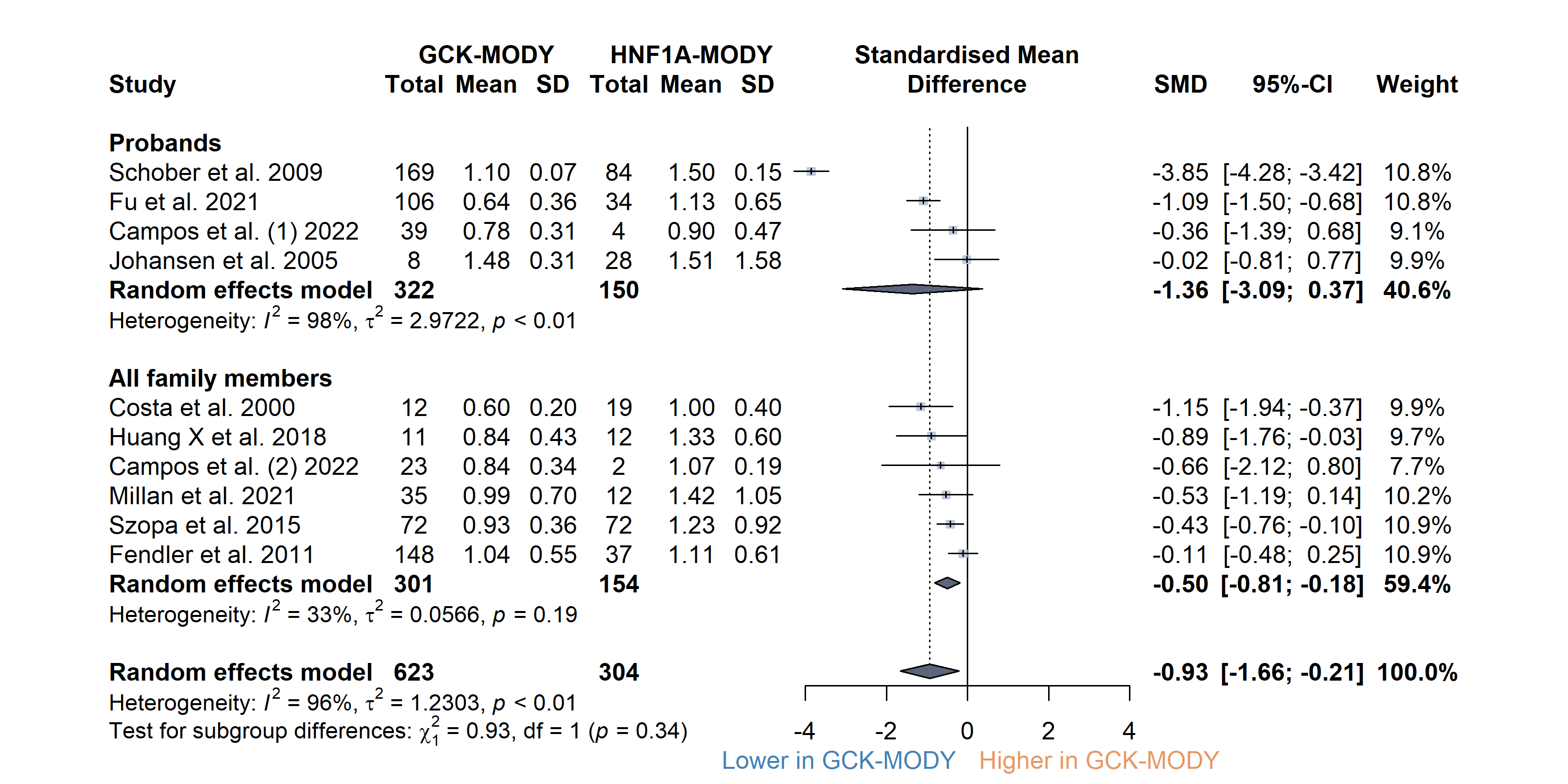


## Figure S30. The forest plot for TG in type 2 diabetes studies


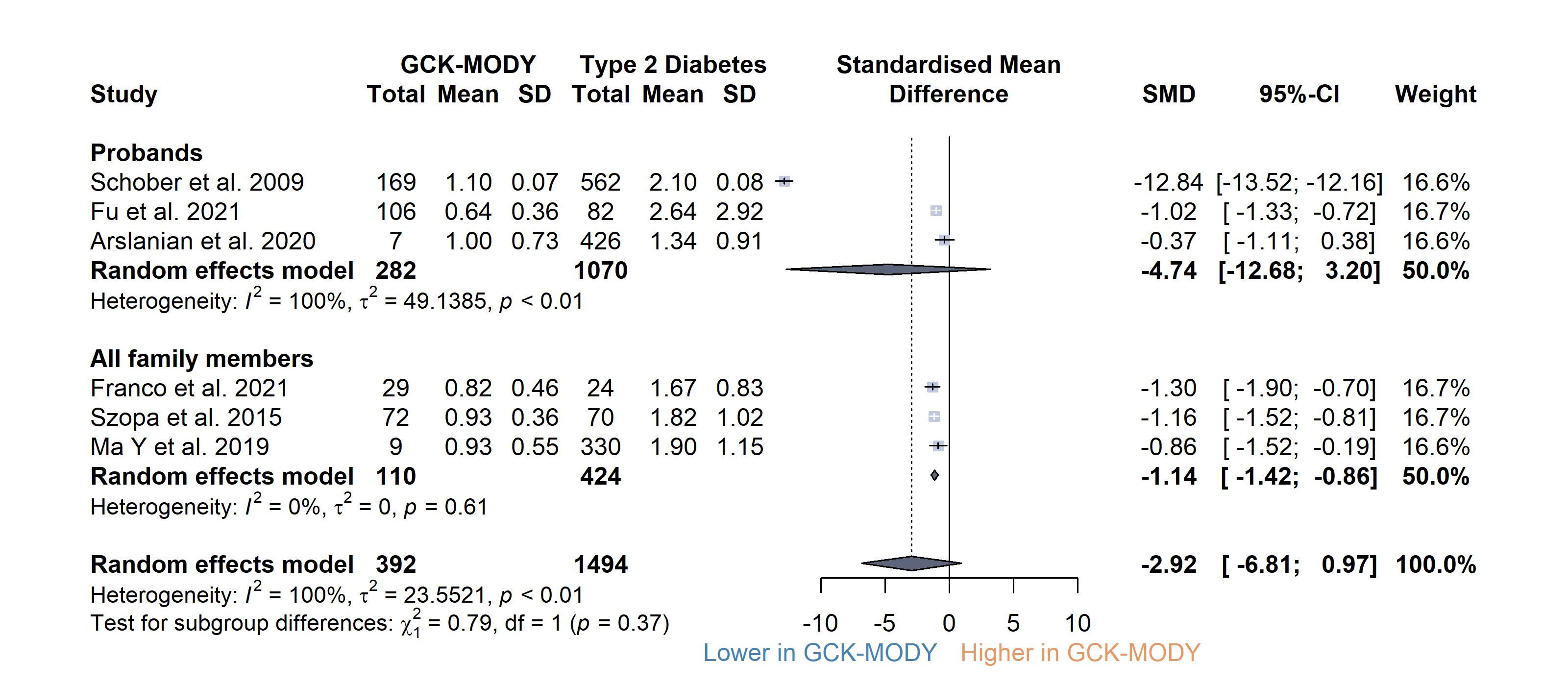


# Materials Relating to the Newcastle-Ottawa Criteria

## Table S5. Definition of terms for bias assessment according to the Newcastle-Ottawa criteria for case-control studies

| **Rating item** | **Definition** |
| --- | --- |
| **Selection** | |
| 1. Adequate case definition | 1 star will be awarded if variations were evaluated as pathogenic or likely pathogenic grade according to the ACMG guideline ^33^. |
| 2. Representativeness  of cases | 1 star will be awarded if the article clearly stated the process of enrollment was a consecutive or random sample from a defined catchment area, and thus to attain a representative sample from the population source. |
| 3. Selection of controls | 1 star will be awarded if it was explicitly stated that patients in comparison groups were recruited consecutively or were from a random sample in the defined catchment area over the same time for the selection of cases, and thus to attain a representative sample from the population source. |
| 4. Definition of controls | *Studies comparing GCK-MODY and HNF1A-MODY patients* 1 star will be awarded if it was clearly specified that included HNF1A-MODY patients were diagnosed by genetic testing and with variants classified as pathogenic or likely pathogenic grade following the ACMG guideline.   *Studies comparing GCK-MODY and type 2 diabetes patients* 1 star will be awarded if all type 2 diabetes patients had no mutation detected, and the included population met the diagnostic criteria with the same cut-off values in the guideline from the American Diabetes Association (ADA) "Standards of Medical Care in Diabetes-2022" ^34^. |
| **Comparability** | |
| 1. Comparability of cases and controls on the basis of the design or analysis | 2 stars will be awarded if studies enrolled all subjects with similar presentations of diabetes, like following MODY criteria.  For studies comparing GCK-MODY and type 2 diabetes patients, 1 star will be awarded if studies enrolled young-onset or well-controlled type 2 diabetes patients when they did not meet the 2-star criterion. |
| **Exposure** |  |
| 1. Ascertainment of exposure | 1 star will be awarded if the study specified the assessment methods (containing the specimen type and the specific assay) used in corresponding assessed clinical indicators. |
| 2. Same method of ascertainment for cases and controls | 1 star will be awarded if the same assessment method and statistical analysis method of the corresponding clinical indicators were used for all subjects in the study. |
| 3. Non-response rate | 1 star will be awarded if studies have more than 80% of patients examined both in the specific case group and comparison group for the specific indicator. |

The maximum number of stars is 9.

For the following two indicators, age at diagnosis and BMI (kg/m^2^), all involved studies were given 1 star in the following two elements in the “Exposure” item, “Ascertainment of exposure” and “Same method of ascertainment for cases and controls”, for the common measurement method around the world.

For details, see <http://www.ohri.ca/programs/clinical_epidemiology/nosgen.pdf>

**Excel files**

Bias assessment of the “Exposure” item for each clinical indicator according to the Newcastle-Ottawa criteria for case-control studies.

Table S6. Bias assessment of the “Exposure” item for each clinical indicator in HNF1A-MODY studies

Table S7. Bias assessment of the “Exposure” item for each clinical indicator in type 2 diabetes studies

## Bias assessment according to the Newcastle-Ottawa criteria for case-control studies

### Table S8. HNF1A-MODY studies except the “Exposure” items

| **Study** | **Year** | **Adequate case definition** | **Representativeness of cases** | **Selection of controls** | **Definition of controls** | **Comparability** | **Subtotal points** | **Information sources** |
| --- | --- | --- | --- | --- | --- | --- | --- | --- |
| Passanisi et al. | 2021 | 1 | 1 | 1 | 1 | 2 | 6 | Article |
| Owen et al. | 2010 | 0 | 0 | 0 | 0 | 0 | 0 | Article |
| Campos et al. (1) | 2022 | 1 | 1 | 1 | 1 | 2 | 6 | Article |
| Campos et al. (2) | 2022 | 1 | 1 | 1 | 1 | 2 | 6 | Article |
| Fu et al. | 2021 | 0 | 1 | 1 | 0 | 2 | 4 | Article |
| Szopa et al. | 2015 | 0 | 0 | 0 | 0 | 0 | 0 | Article, Skupien et al. ^35^ |
| Chèvre et al. | 1998 | 0 | 0 | 0 | 0 | 2 | 2 | Article |
| Costa et al. | 2000 | 0 | 0 | 0 | 0 | 2 | 2 | Article |
| Pruhova et al. | 2003 | 0 | 1 | 1 | 0 | 2 | 4 | Article |
| Fendler et al. | 2011 | 0 | 1 | 0 | 0 | 0 | 1 | Article, Skupien et al. ^35^ |
| Johansen et al. | 2005 | 0 | 1 | 1 | 0 | 2 | 4 | Article |
| Stankute et al. | 2020 | 1 | 1 | 1 | 1 | 2 | 6 | Article, Verkauskiene et al. ^36^ |
| Lorini et al. | 2009 | 0 | 1 | 1 | 0 | 2 | 4 | Article, Lorini et al. ^37^ |
| Millan et al. | 2021 | 0 | 0 | 0 | 0 | 2 | 2 | Article |
| Gökşen et al. | 2021 | 1 | 1 | 1 | 1 | 2 | 6 | Article |
| Donath et al. | 2019 | 1 | 1 | 1 | 1 | 2 | 6 | Article |
| Barrio et al. | 2002 | 0 | 0 | 0 | 0 | 2 | 2 | Article |
| Schober et al. | 2009 | 0 | 0 | 0 | 0 | 2 | 2 | Article |
| Pihoker et al. | 2013 | 0 | 1 | 1 | 0 | 0 | 2 | Article |
| Bacon et al | 2012 | 0 | 0 | 0 | 0 | 0 | 0 | Article, Kyithar et al. ^38^ |
| Nowak et al. | 2013 | 0 | 0 | 0 | 0 | 0 | 0 | Article |
| Yorifuji et al. | 2012 | 0 | 1 | 1 | 0 | 2 | 4 | Article |
| Huang X et al. | 2018 | 0 | 0 | 0 | 0 | 0 | 0 | Article, Liu et al. ^39^ |

### Table S9. Type 2 diabetes studies except the “Exposure” items

| **Study** | **Year** | **Adequate case definition** | **Representativeness of cases** | **Selection of controls** | **Definition of controls** | **Comparability** | **Subtotal points** | **Information**  **sources** |
| --- | --- | --- | --- | --- | --- | --- | --- | --- |
| Owen et al. | 2010 | 0 | 0 | 0 | 0 | 1 | 1 | Article |
| Arslanian et al. | 2020 | 1 | 1 | 1 | 1 | 2 | 6 | Article, Arslanian et al. ^40^ |
| Franco et al. | 2021 | 1 | 1 | 1 | 0 | 1 | 4 | Article, Giuffrida et al. ^41^ |
| Fu et al. | 2021 | 0 | 1 | 1 | 0 | 1 | 3 | Article |
| Szopa et al. | 2015 | 0 | 0 | 0 | 0 | 0 | 0 | Article, Skupien et al. ^35^ |
| Pacaud et al. | 2016 | 0 | 0 | 0 | 0 | 1 | 1 | Article |
| Velho et al. | 1996 | 0 | 1 | 1 | 1 | 0 | 3 | Article, Chevre et al. ^2^, Froguel et al. ^42^ |
| Sagen et al. | 2008 | 0 | 1 | 1 | 0 | 2 | 4 | Article |
| Elias-Assad et al. | 2021 | 1 | 1 | 1 | 1 | 2 | 6 | Article |
| Agladioglu et al. | 2016 | 0 | 1 | 1 | 0 | 2 | 4 | Article |
| Schober et al. | 2009 | 0 | 0 | 0 | 0 | 1 | 1 | Article |
| Dusatkova et al. | 2022 | 0 | 1 | 0 | 0 | 1 | 2 | Article, Pruhova et al. ^43^ |
| McDonald et al. | 2011 | 0 | 0 | 0 | 0 | 0 | 0 | Article |
| Ma Y et al. | 2019 | 1 | 1 | 1 | 1 | 0 | 4 | Article, Liu et al. ^39^ |

# GRADE Profiles

## Table S10. GRADE evidence profile for clinical indicators quantified in ≧2 studies in HNF1A-MODY studies

| Clinical indicators | No. of studies | Study limitations | Inconsistency | Indirectness | Imprecision | Publication bias | Overall quality |
| --- | --- | --- | --- | --- | --- | --- | --- |
| Age at diagnosis | 17 | No serious limitations | Very serious inconsistency | No serious indirectness | No serious imprecision | Undetected | Low ⊕⊕○○ |
| Birth Weight | 5 | No serious limitations | Very serious inconsistency | No serious indirectness | Very serious imprecision | Undetected | Very Low ⊕○○○ |
| BM (SDS) | 5 | No serious limitations | Very serious inconsistency | No serious indirectness | Serious imprecision | Undetected | Very Low ⊕○○○ |
| BMI (kg/m^2^) | 15 | No serious limitations | Very serious inconsistency | No serious indirectness | No serious imprecision | Undetected | Low ⊕⊕○○ |
| hsCRP | 3 | Serious limitations | Very serious inconsistency | No serious indirectness | Serious imprecision | Undetected | Very Low ⊕○○○ |
| HbA1c | 18 | No serious limitations | Very serious inconsistency | No serious indirectness | No serious imprecision | Undetected | Low ⊕⊕○○ |
| FPG | 18 | No serious limitations | Very serious inconsistency | No serious indirectness | No serious imprecision | Undetected | Low ⊕⊕○○ |
| 2h PG | 5 | No serious limitations | Very serious inconsistency | No serious indirectness | Serious imprecision | Undetected | Very Low ⊕○○○ |
| Δ PG | 3 | No serious limitations | No serious inconsistency | No serious indirectness | Very serious imprecision | Undetected | Low ⊕⊕○○ |
| FCP | 9 | No serious limitations | No serious inconsistency | No serious indirectness | No serious imprecision | Undetected | High ⊕⊕⊕⊕ |
| insulin | 6 | No serious limitations | Serious inconsistency | No serious indirectness | No serious imprecision | Undetected | Moderate ⊕⊕⊕○ |
| TC | 10 | No serious limitations | Serious inconsistency | No serious indirectness | No serious imprecision | Undetected | Moderate ⊕⊕⊕○ |
| LDL-C | 7 | Serious limitations | No serious inconsistency | No serious indirectness | No serious imprecision | Undetected | Moderate ⊕⊕⊕○ |
| HDL-C | 9 | No serious limitations | Very serious inconsistency | No serious indirectness | No serious imprecision | Undetected | Low ⊕⊕○○ |
| TG | 10 | Serious limitations | Very serious inconsistency | No serious indirectness | No serious imprecision | Undetected | Very Low ⊕○○○ |

## Table S11. GRADE evidence profile for clinical indicators quantified in ≧2 studies in type 2 diabetes studies

| **Clinical indicators** | **No. of studies** | **Study limitations** | **Inconsistency** | **Indirectness** | **Imprecision** | **Publication bias** | **Overall quality** |
| --- | --- | --- | --- | --- | --- | --- | --- |
| Age at diagnosis | 12 | No serious limitations | Very serious inconsistency | No serious indirectness | No serious imprecision | Undetected | Low ⊕⊕○○ |
| BM (SDS) | 3 | No serious limitations | Very serious inconsistency | No serious indirectness | No serious imprecision | Undetected | Low ⊕⊕○○ |
| BMI (kg/m^2^) | 11 | No serious limitations | Very serious inconsistency | No serious indirectness | No serious imprecision | Undetected | Low ⊕⊕○○ |
| hsCRP | 4 | Serious limitations | No serious inconsistency | No serious indirectness | No serious imprecision | Undetected | Moderate ⊕⊕⊕○ |
| HbA1c | 13 | No serious limitations | Very serious inconsistency | No serious indirectness | No serious imprecision | Undetected | Low ⊕⊕○○ |
| FPG | 9 | No serious limitations | Very serious inconsistency | No serious indirectness | No serious imprecision | Undetected | Low ⊕⊕○○ |
| 2h PG | 2 | No serious limitations | Very serious inconsistency | No serious indirectness | No serious imprecision | Undetected | Low ⊕⊕○○ |
| FCP | 3 | No serious limitations | Serious inconsistency | No serious indirectness | Serious imprecision | Undetected | Low ⊕⊕○○ |
| insulin | 3 | No serious limitations | Very serious inconsistency | No serious indirectness | No serious imprecision | Undetected | Low ⊕⊕○○ |
| TC | 6 | No serious limitations | Very serious inconsistency | No serious indirectness | No serious imprecision | Undetected | Low ⊕⊕○○ |
| LDL-C | 6 | No serious limitations | Very serious inconsistency | No serious indirectness | No serious imprecision | Undetected | Low ⊕⊕○○ |
| HDL-C | 5 | Serious limitations | Very serious inconsistency | No serious indirectness | No serious imprecision | Undetected | Very Low ⊕○○○ |
| TG | 6 | No serious limitations | Very serious inconsistency | No serious indirectness | No serious imprecision | Undetected | Low ⊕⊕○○ |

As all studies had applied genetic testing to diagnose monogenic diabetes and were filtered strictly according to the eligibility criteria, we chose the starting point of “high quality” for all analyses. The quality was rated downwards based on the following items.

**Study limitations**: based on the mean Newcastle-Ottawa criteria for case-control studies (Supplementary Table S8. and Table S9.) total scores for the included studies; rating -1 when <5 and -2 when <3.

**Inconsistency**: based on the point estimate variation, CI overlap, I^2^ and *P*-value for heterogeneity (*P* *^h^*); rating -1 if I^2^ > 50% or *P* *^h^*<0.10 and -2 if I^2^ > 75% or *P* *^h^* <0.01.

**Indirectness**: based on differences in patients, interventions, outcomes, and head-to-head comparison. As this is addressed in study limitations, all analyses were rated as “no serious indirectness”.

**Imprecision**: based on sample size requirements for effect sizes of SMD = 0.4 and 0.2; rating -1 if the total number of cases or total number of controls were < 200 and -2 if < 100.

**Publication bias**: evaluated with funnel plots for biomarkers assessed in 10 or more studies.

# Funnel Plots for Publication Bias and Sensitivity Analysis

## HNF1A-MODY studies

### Figure S31. The contour-enhanced funnel plot for age at diagnosis in HNF1A-MODY studies


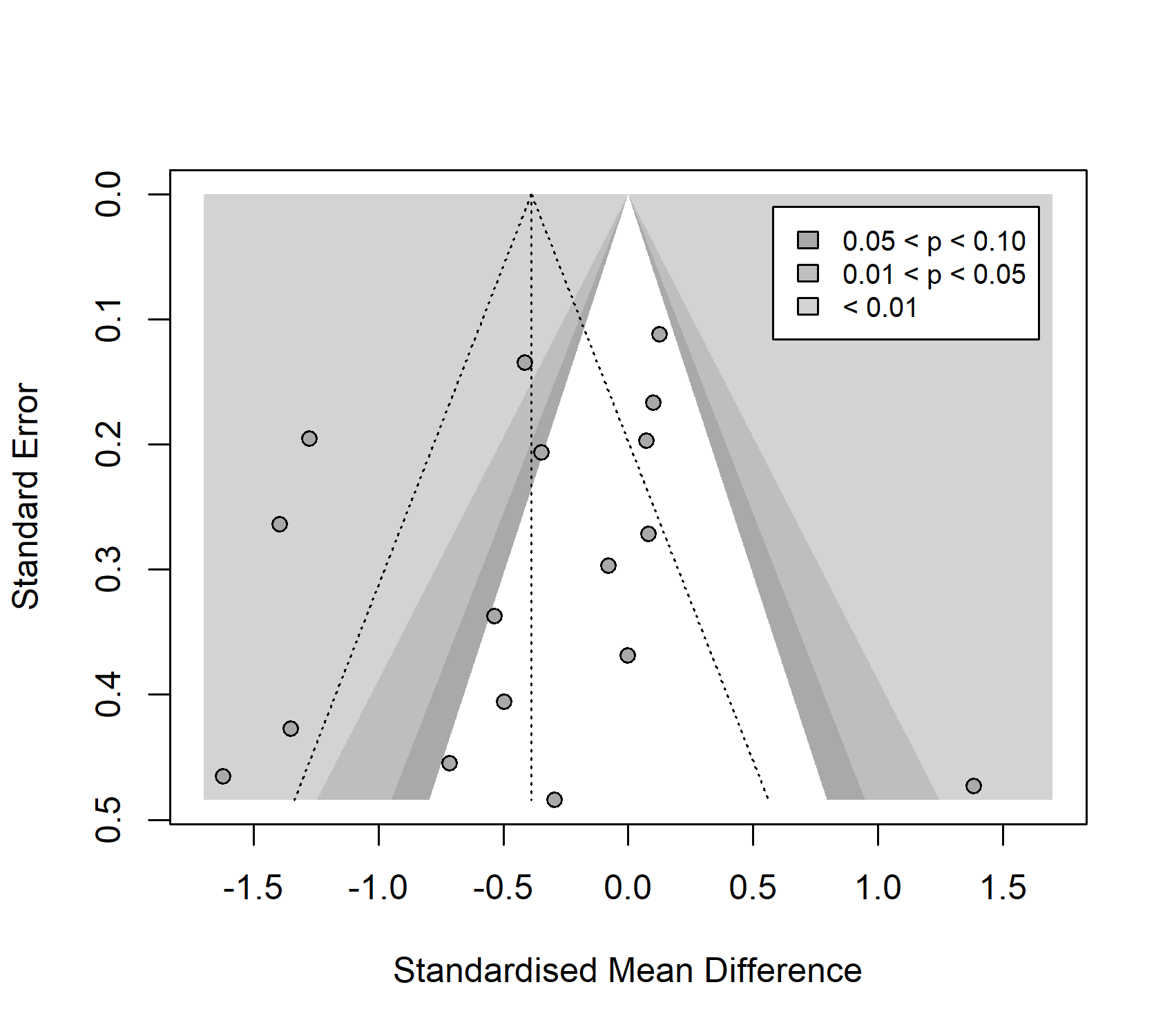


### Figure S32. The trim-and-fill funnel plot for age at diagnosis in HNF1A-MODY studies


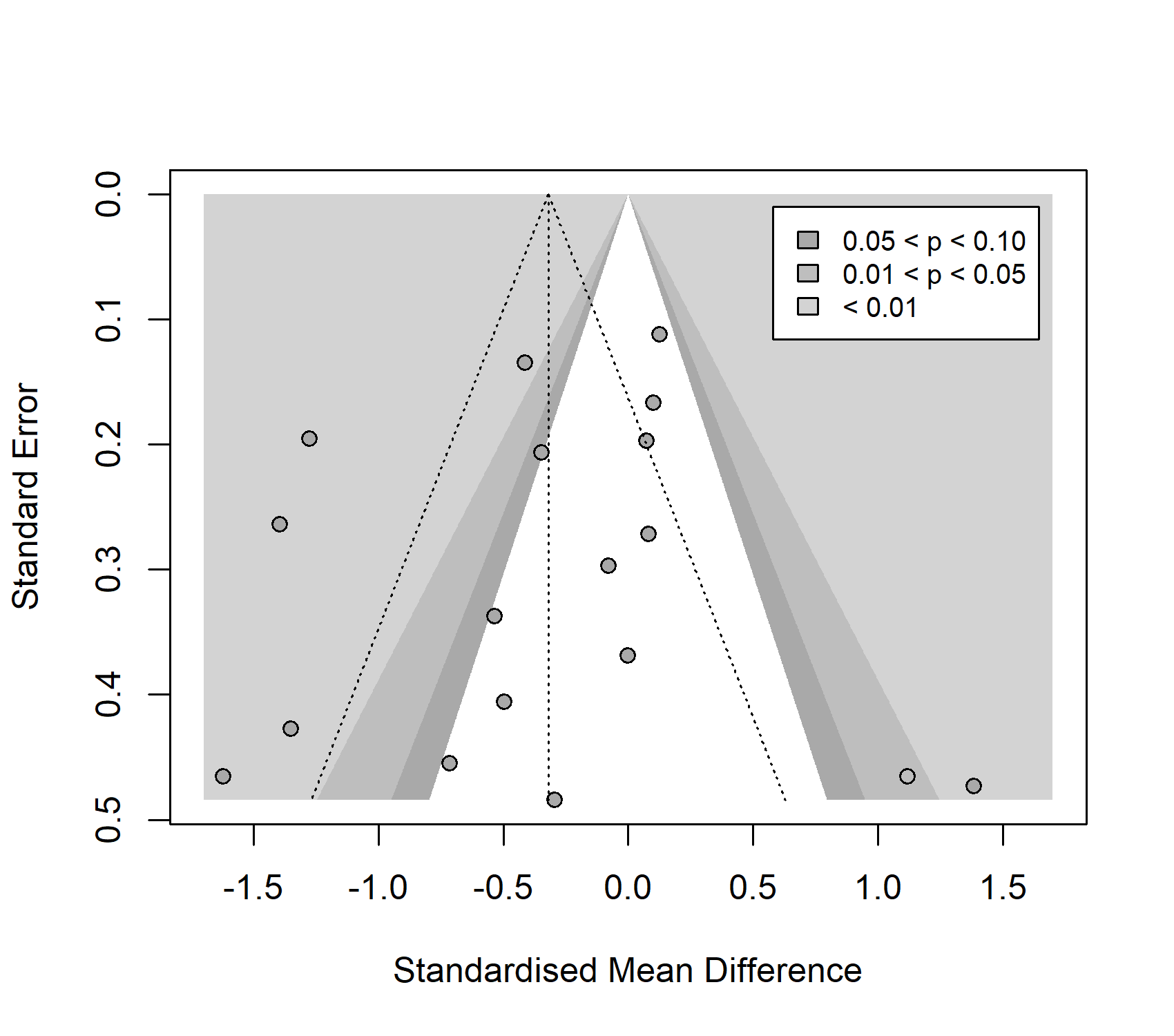


### Figure S33. The contour-enhanced funnel plot for BMI (kg/m^2^) in HNF1A-MODY studies


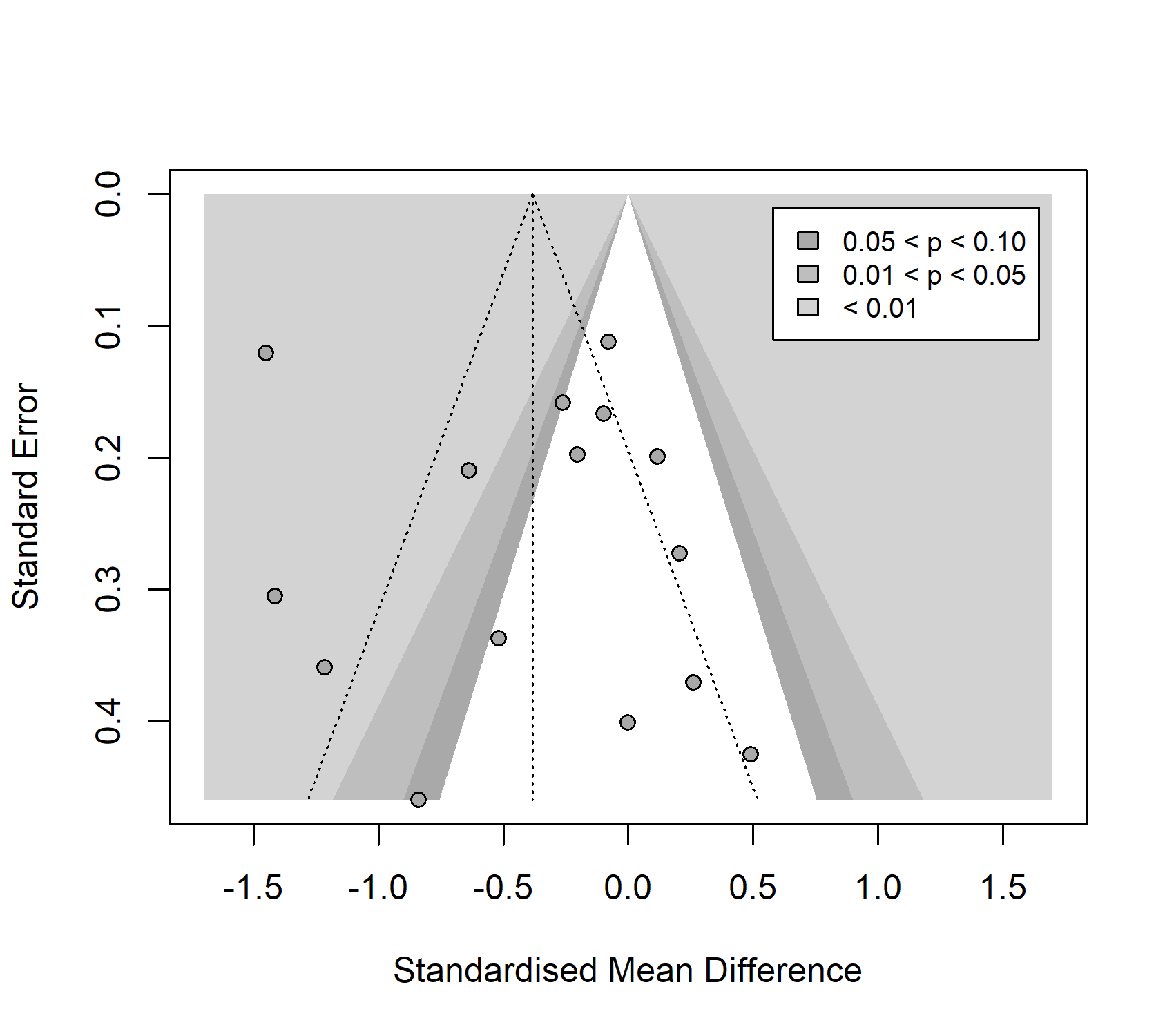


### Figure S34. The trim-and-fill funnel plot for BMI (kg/m^2^) in HNF1A-MODY studies


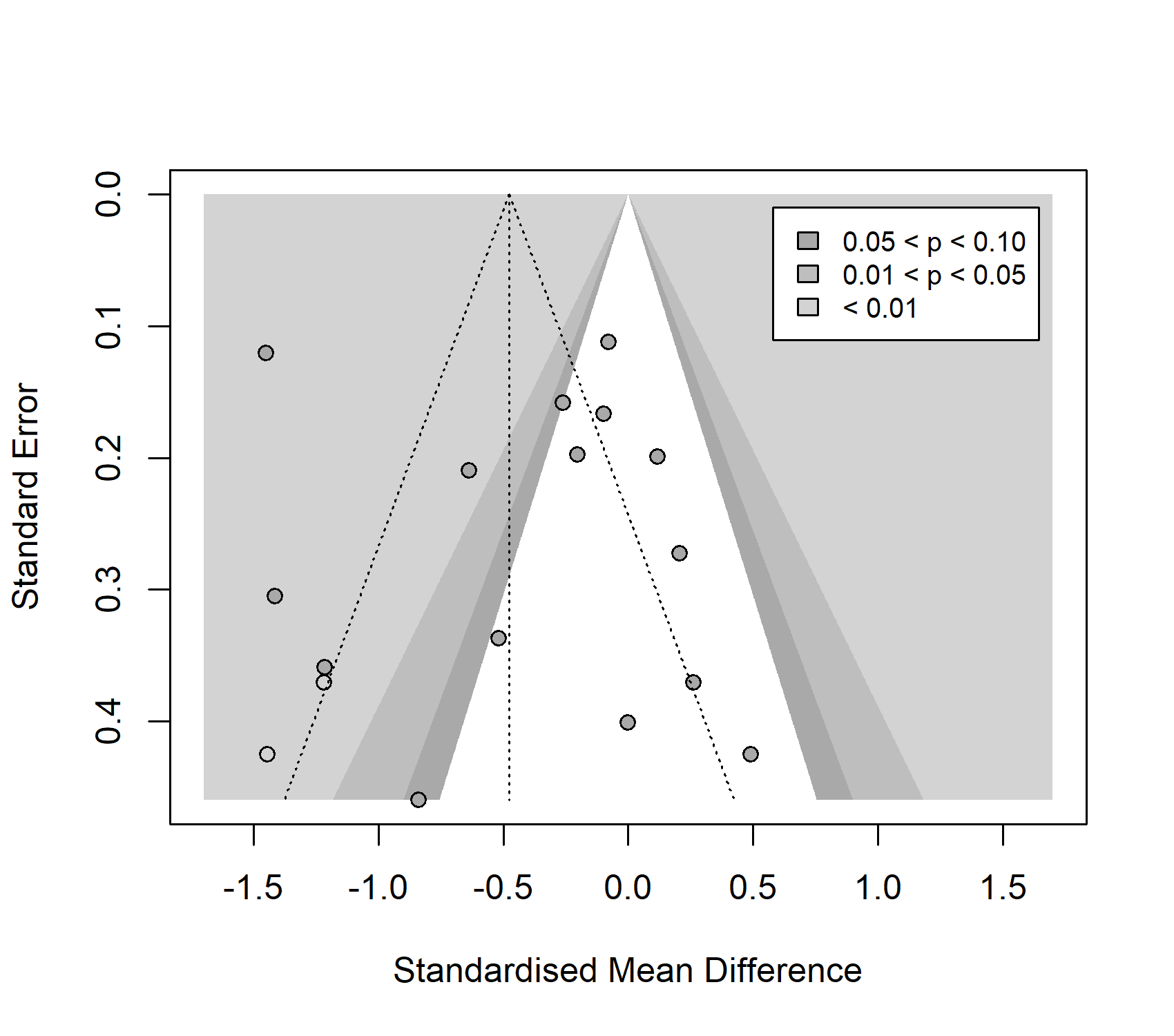


### Figure S35. The contour-enhanced funnel plot for HbA1c in HNF1A-MODY studies


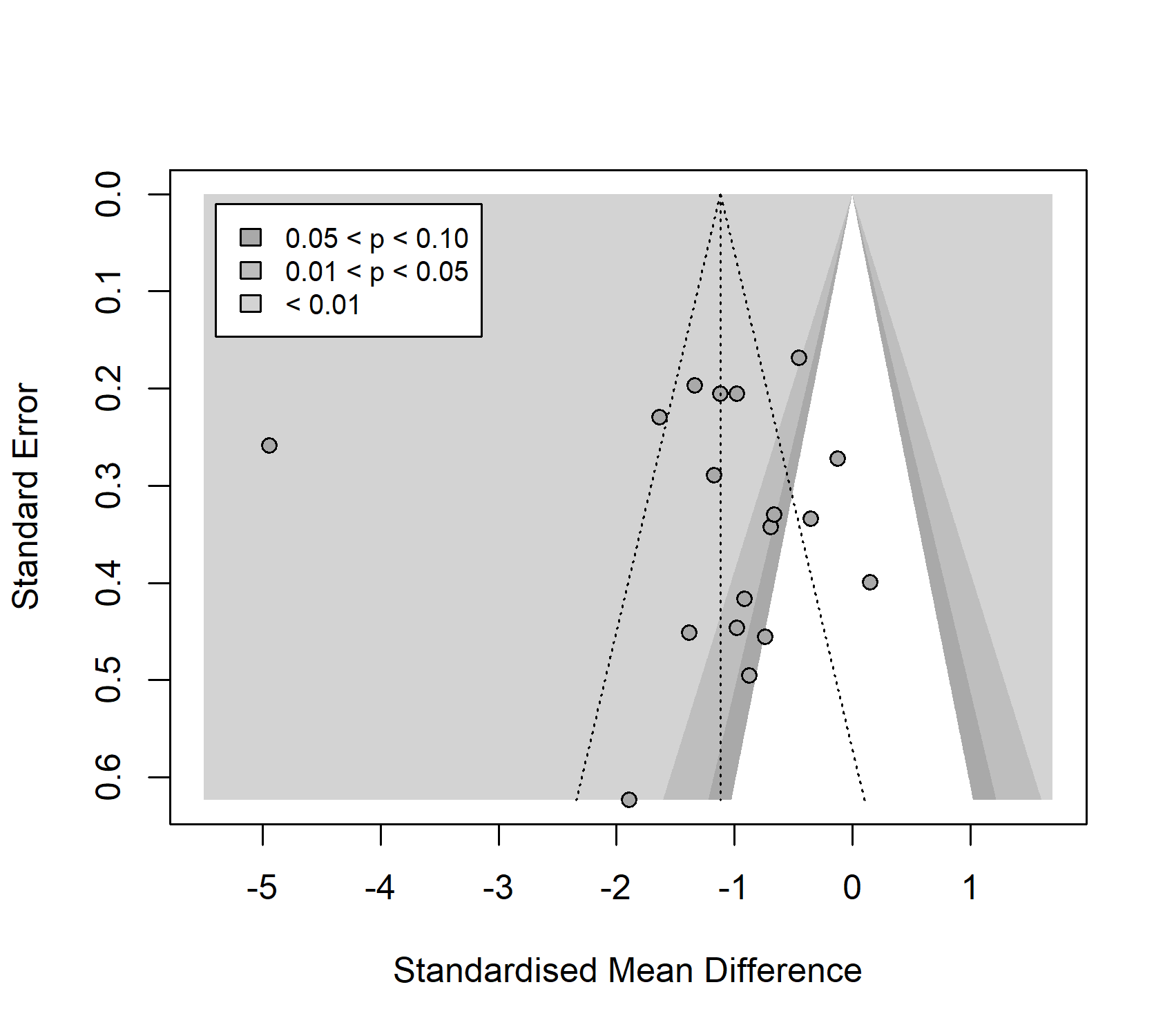


### Figure S36. The trim-and-fill funnel plot for HbA1c in HNF1A-MODY studies


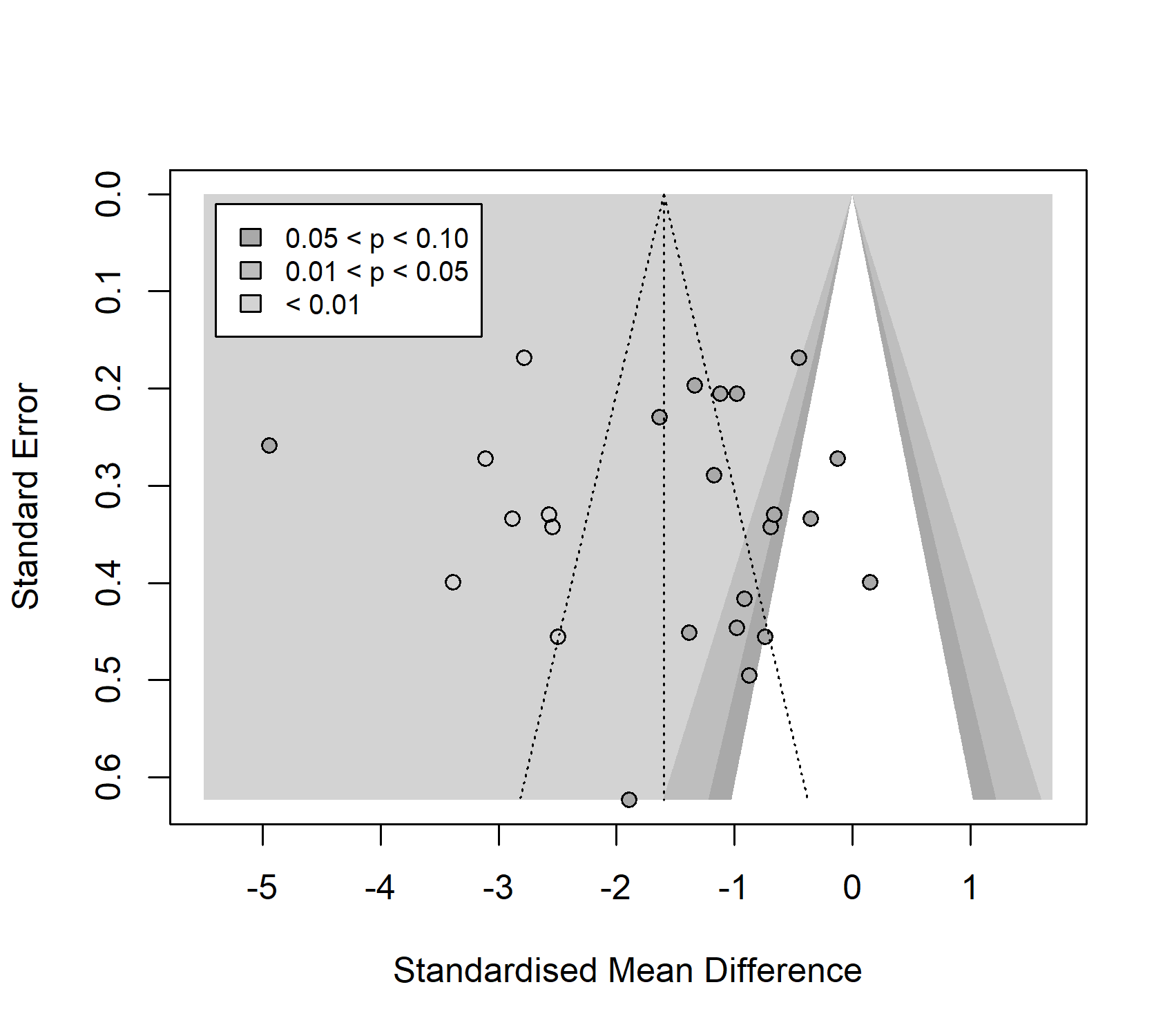


### Figure S37. The contour-enhanced funnel plot for fasting blood glucose in HNF1A-MODY studies


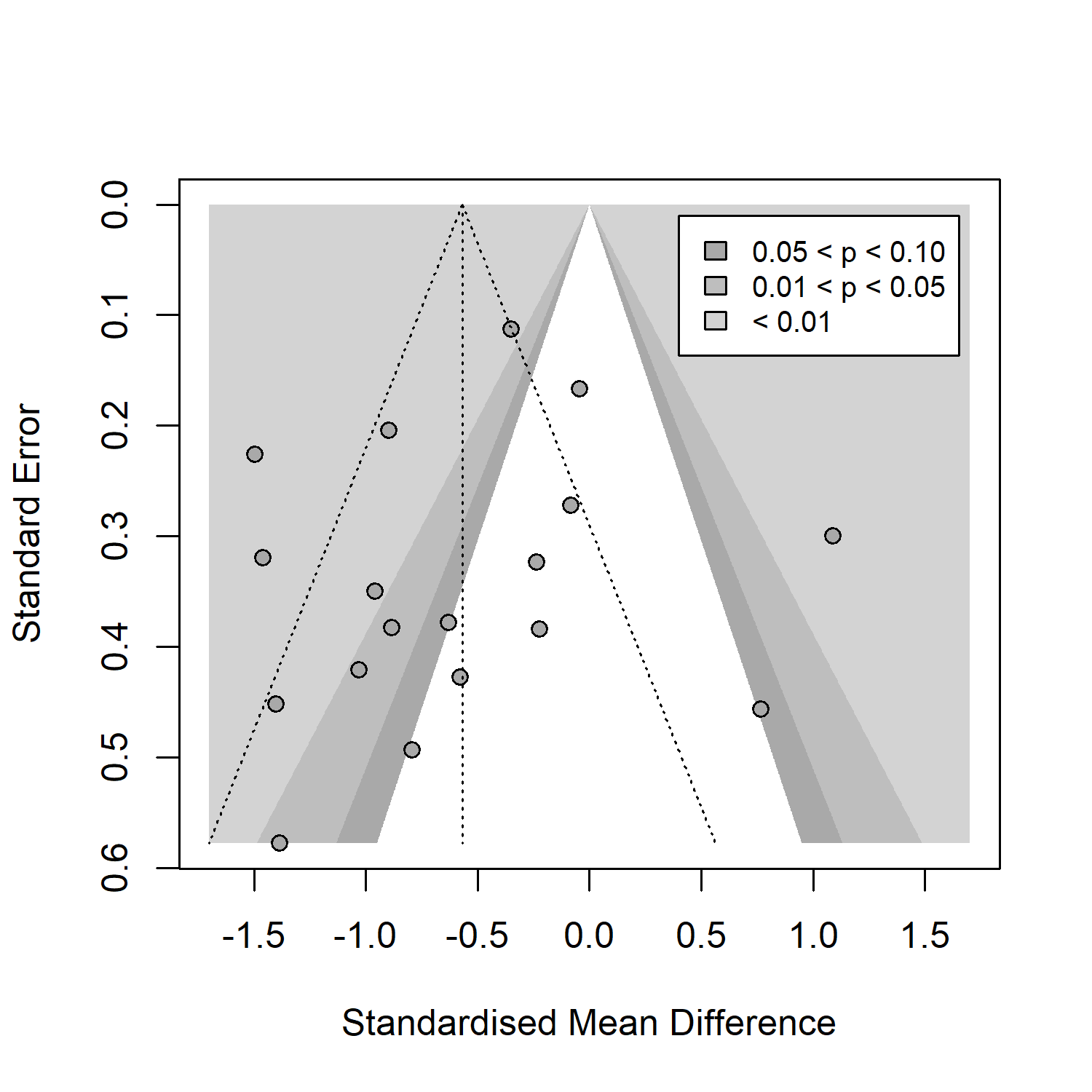


### Figure S38. The trim-and-fill funnel plot for fasting blood glucose in HNF1A-MODY studies


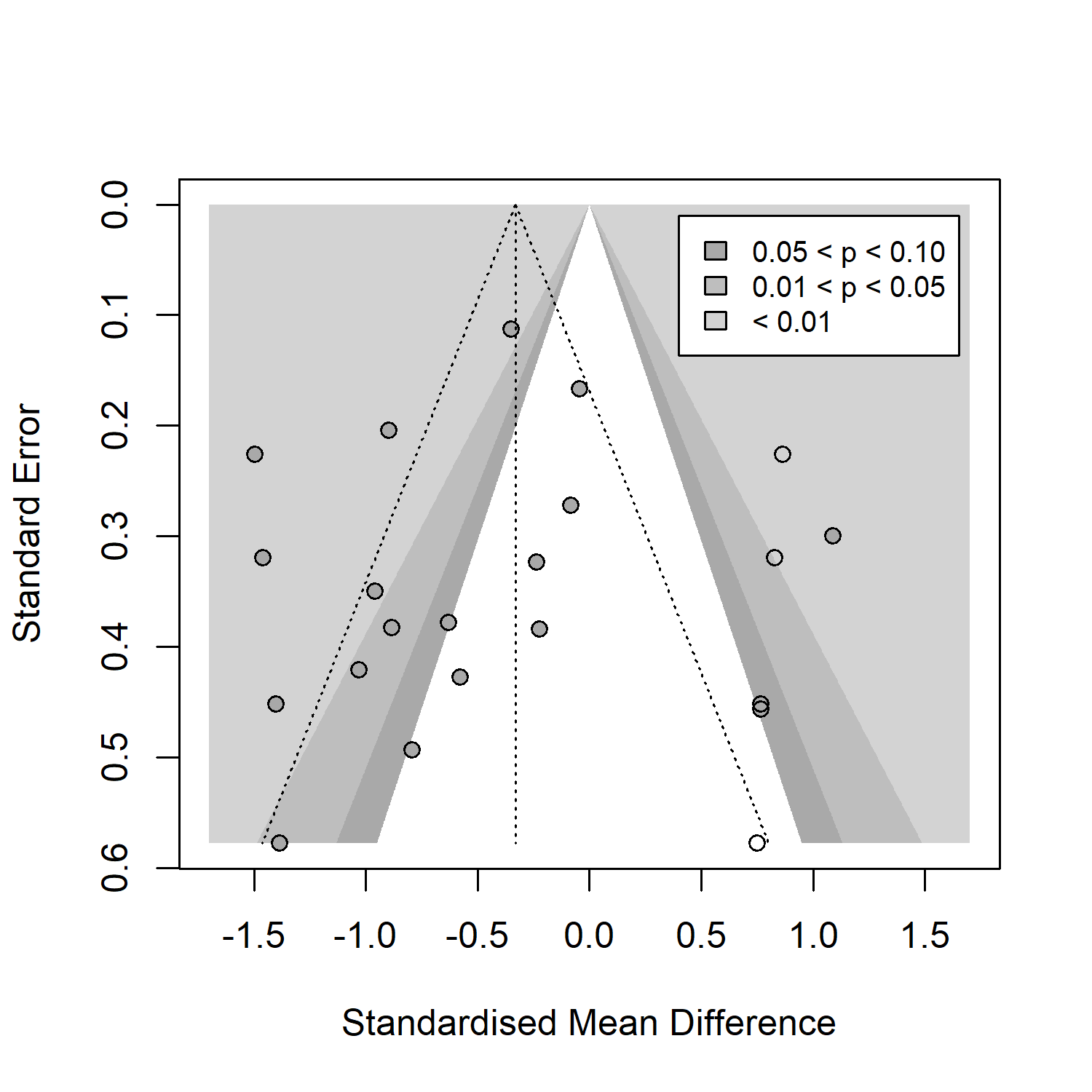


### Figure S39. The contour-enhanced funnel plot for TC in HNF1A-MODY studies


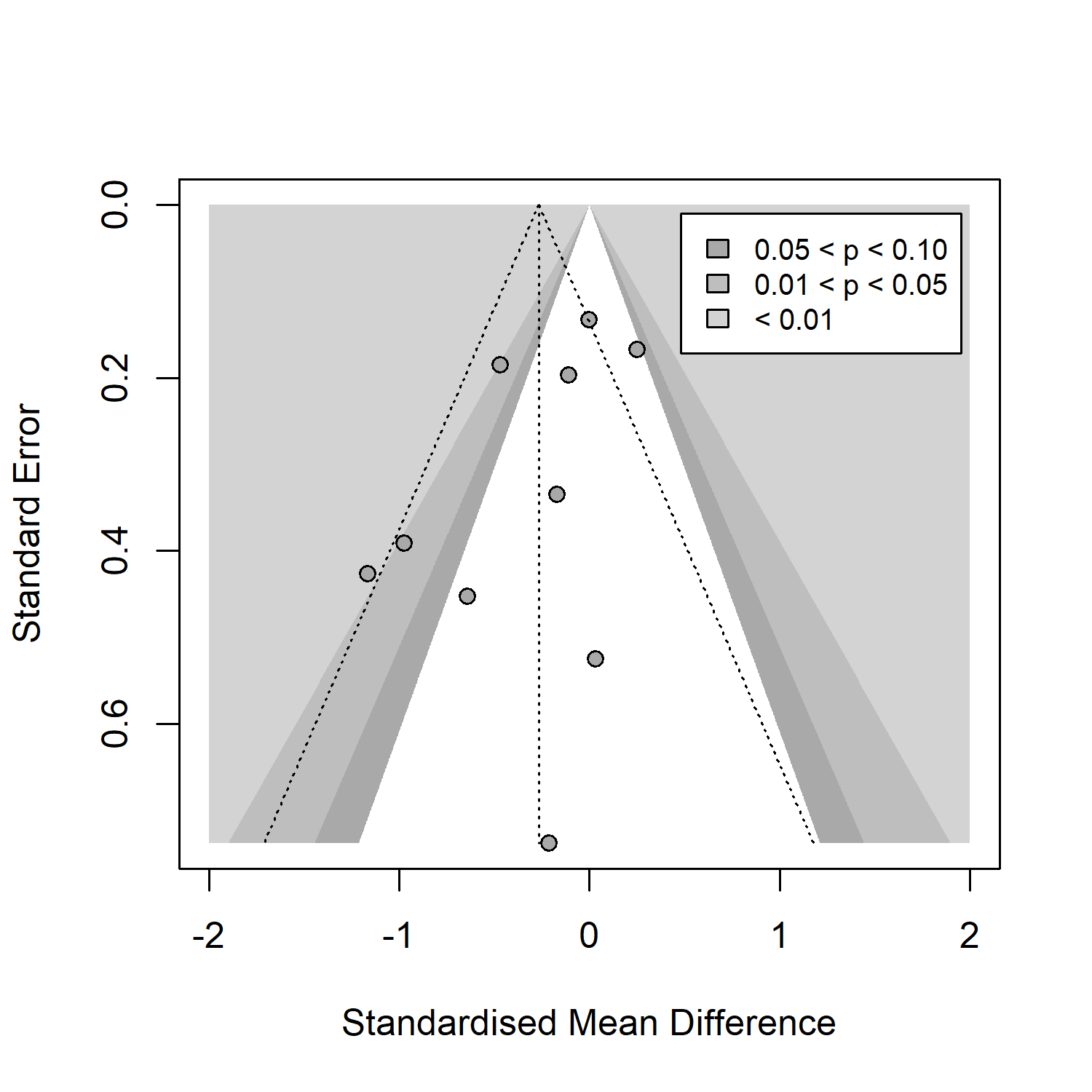


### Figure S40. The trim-and-fill funnel plot for TC in HNF1A-MODY studies


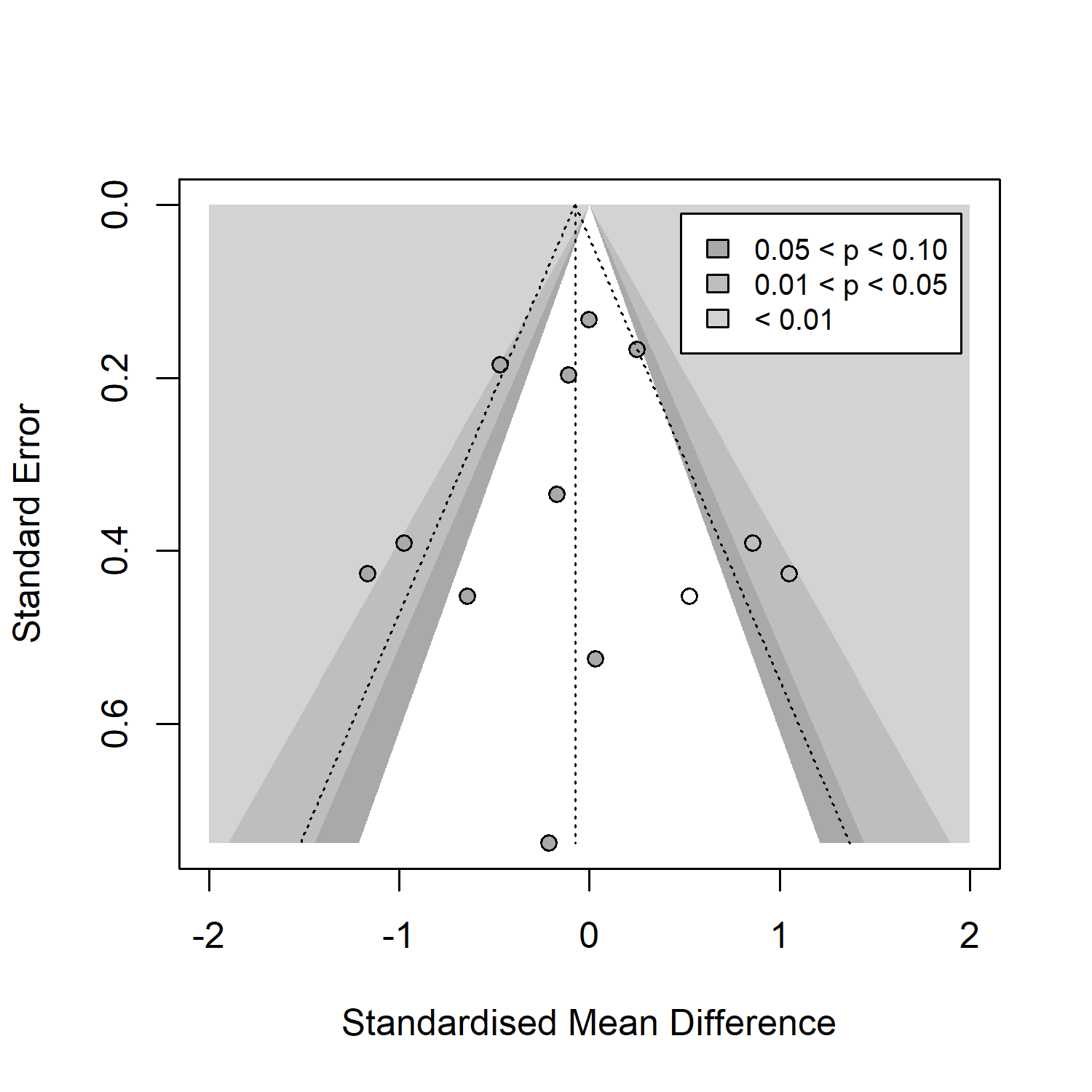


### Figure S41. The contour-enhanced funnel plot for TG in HNF1A-MODY studies


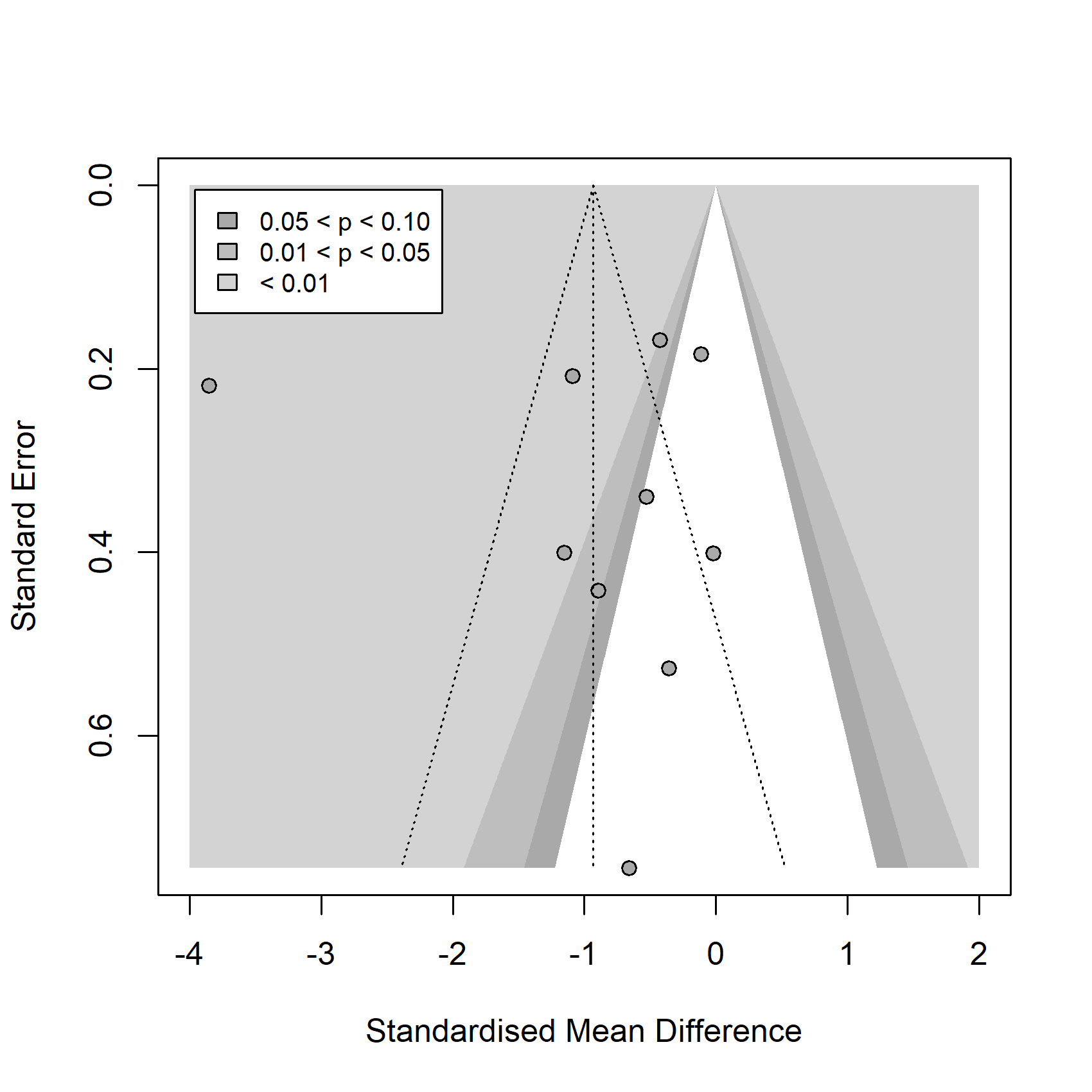


### Figure S42. The trim-and-fill funnel plot for TG in HNF1A-MODY studies


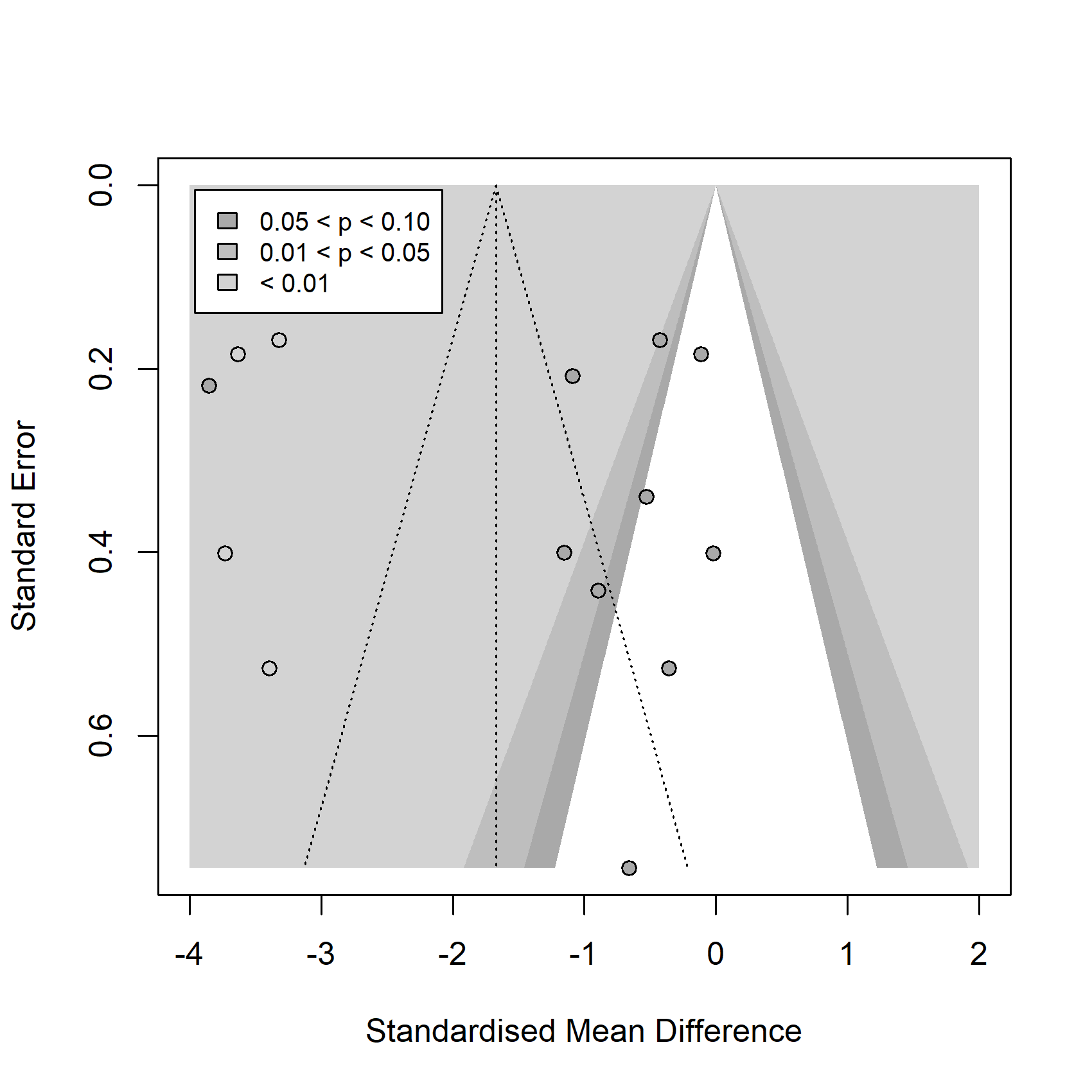


## Type 2 diabetes studies

### Figure S43. The contour-enhanced funnel plot for age at diagnosis in type 2 diabetes studies


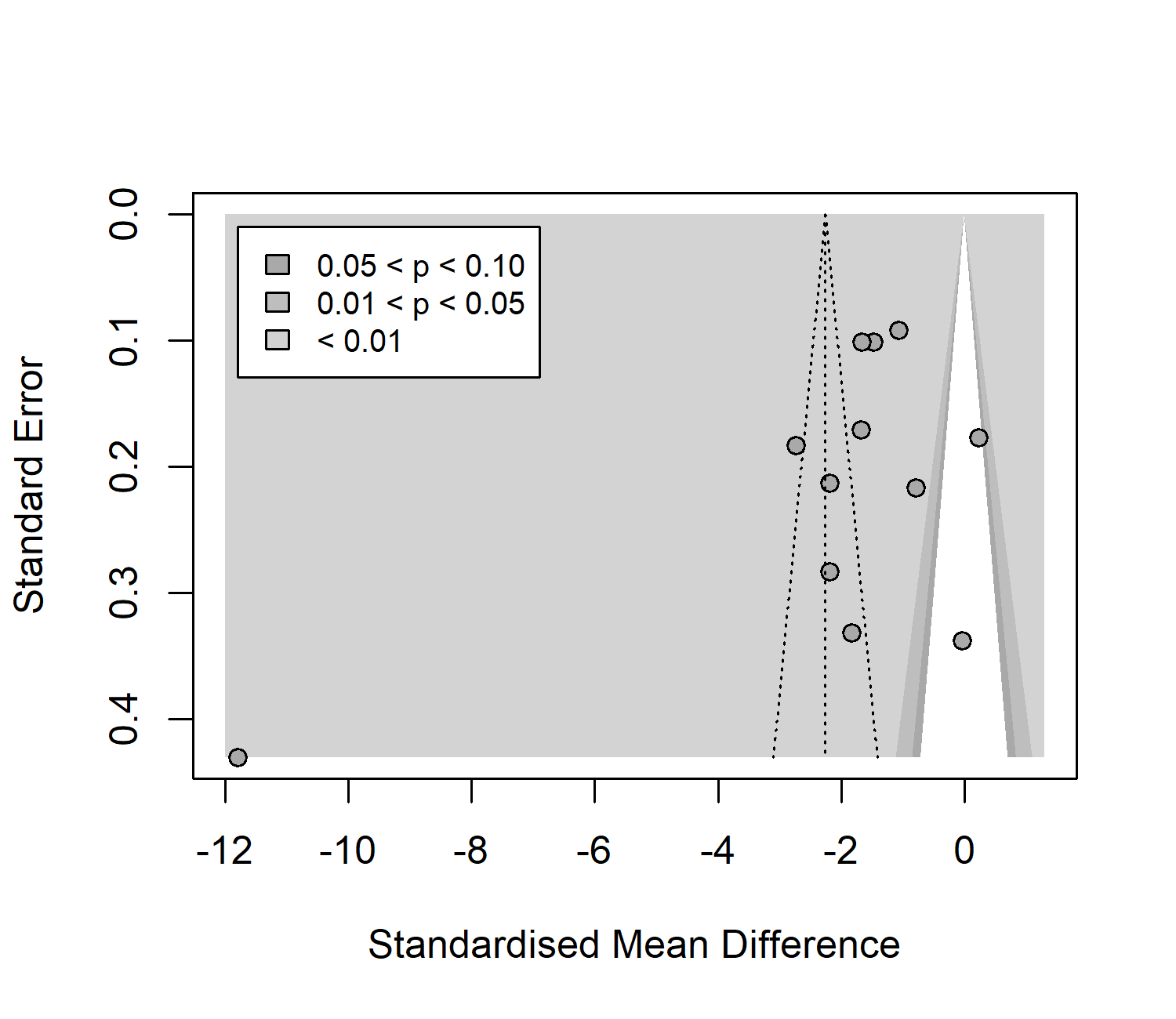


### Figure S44. The trim-and-fill funnel plot for age at diagnosis in type 2 diabetes studies


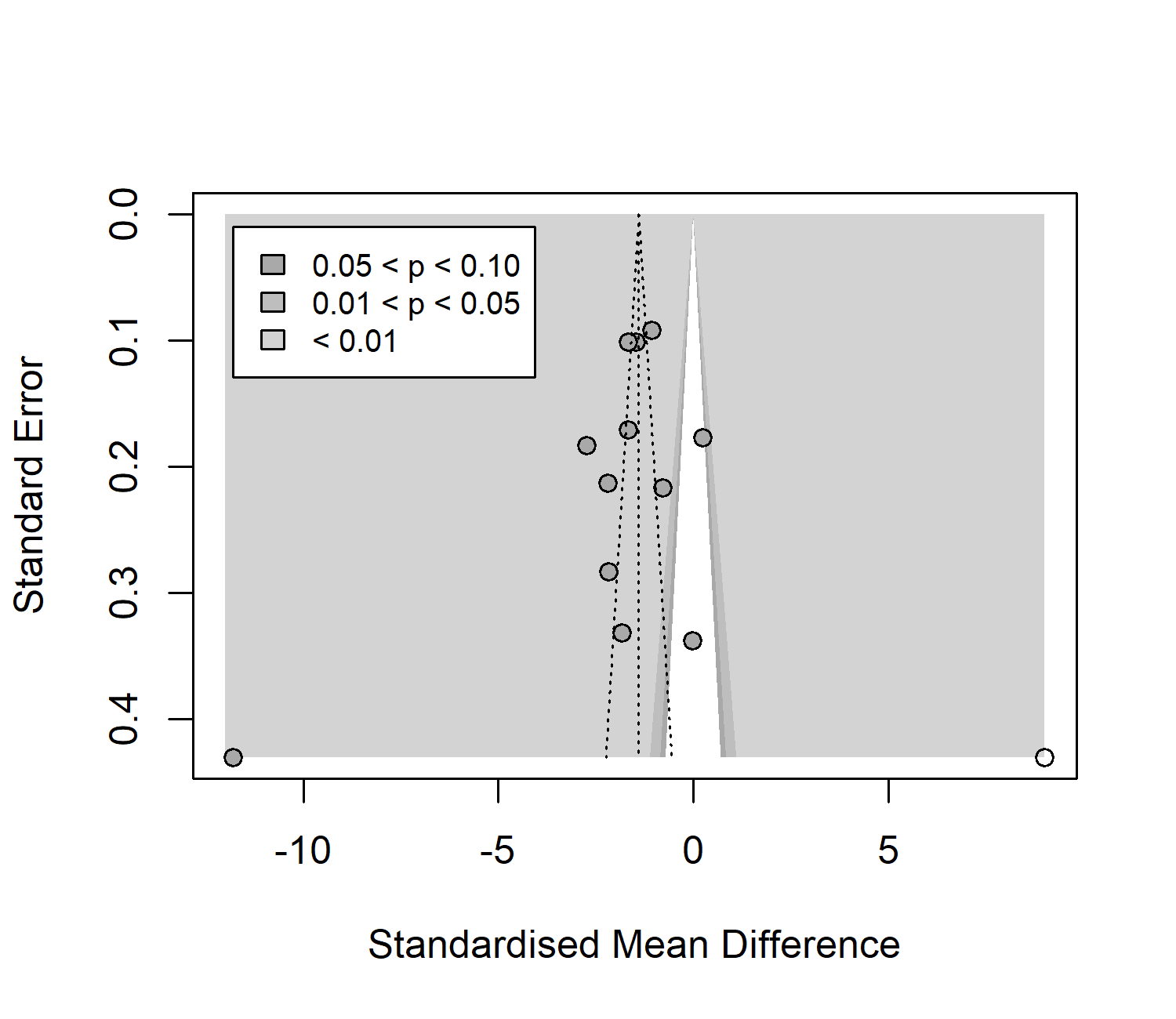


### Figure S45. The contour-enhanced funnel plot for BMI (kg/m^2^) in type 2 diabetes studies


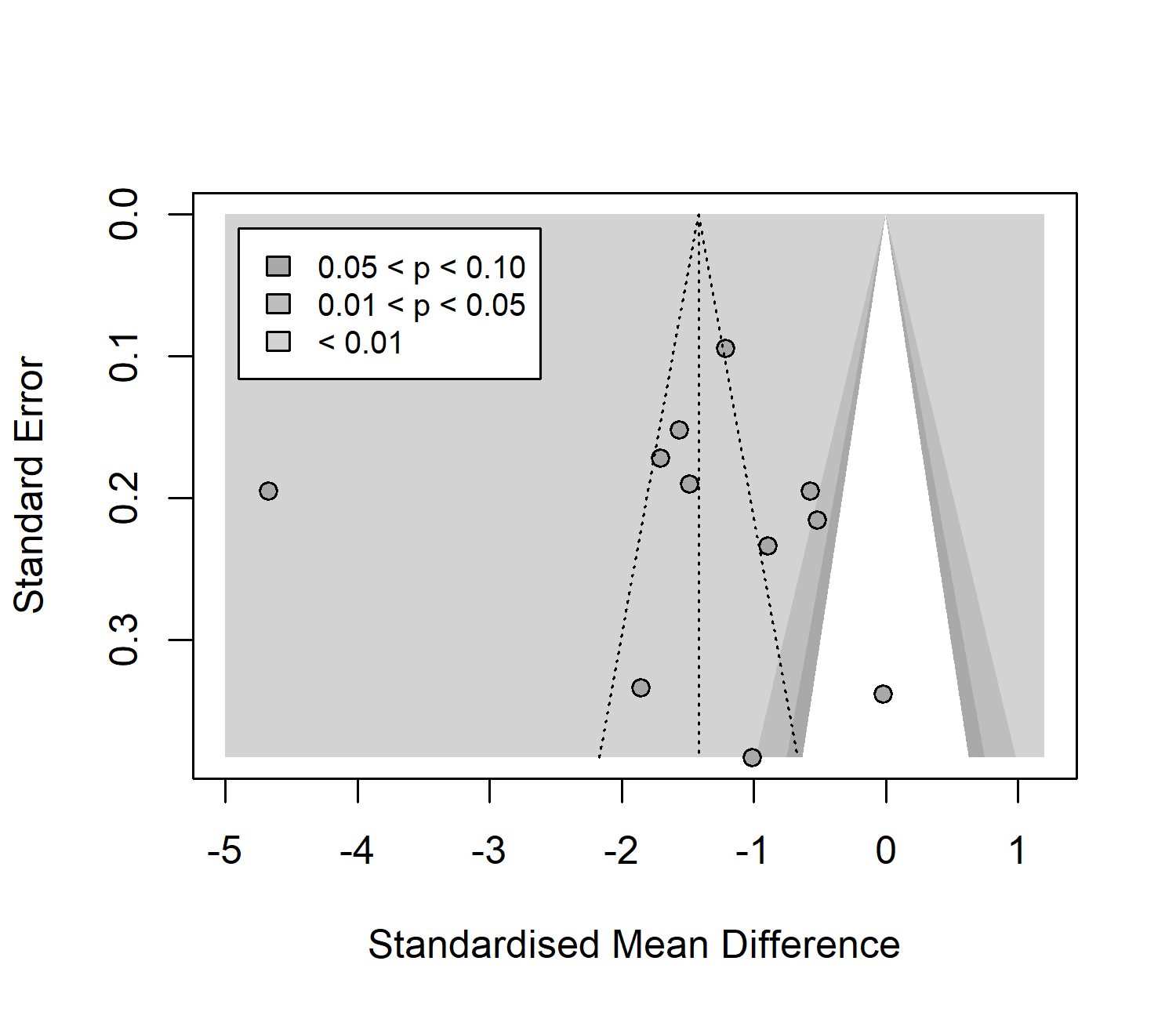


### Figure S46. The trim-and-fill funnel plot for BMI (kg/m^2^) in type 2 diabetes studies


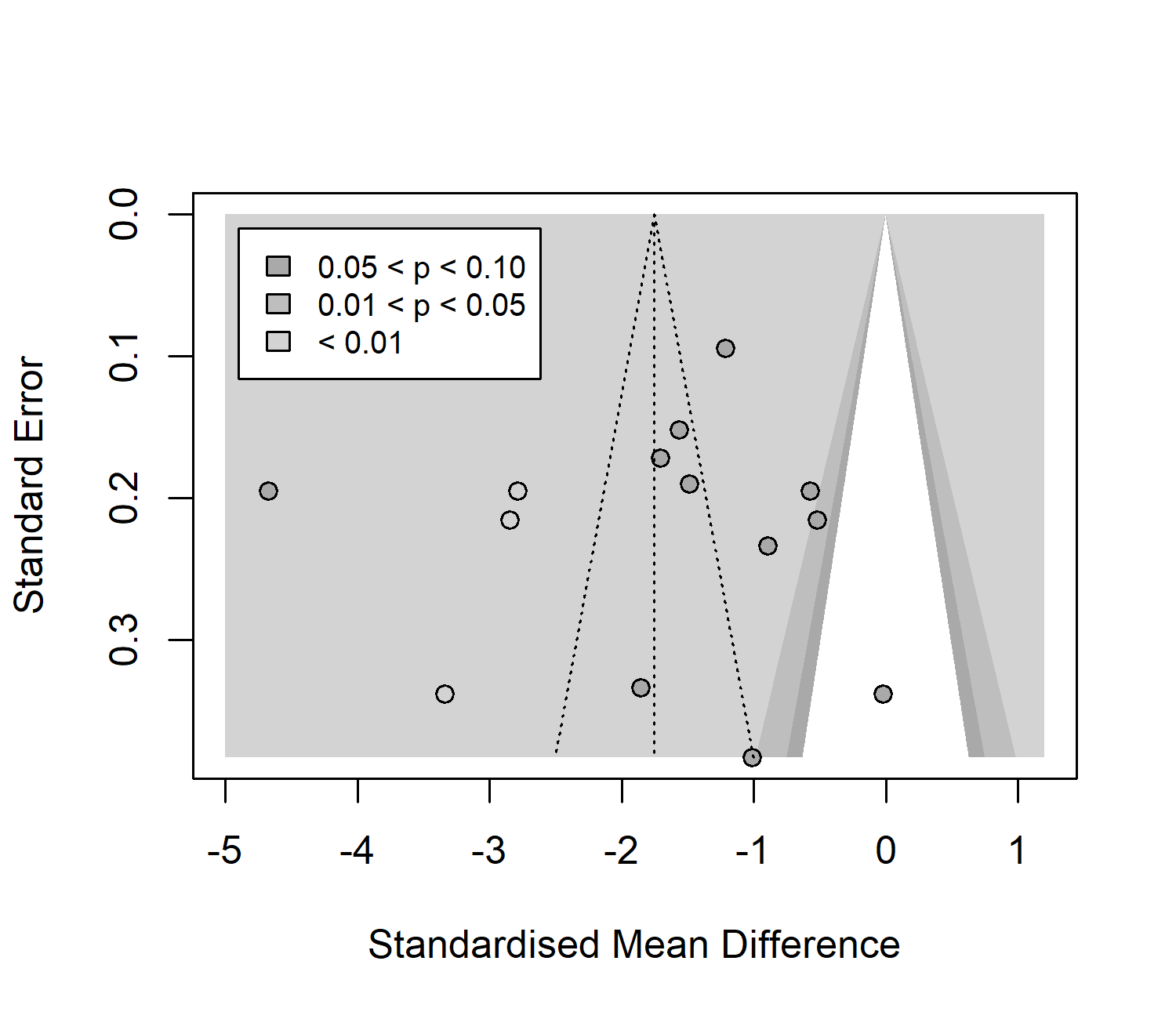


### Figure S47. The contour-enhanced funnel plot for HbA1c in type 2 diabetes studies


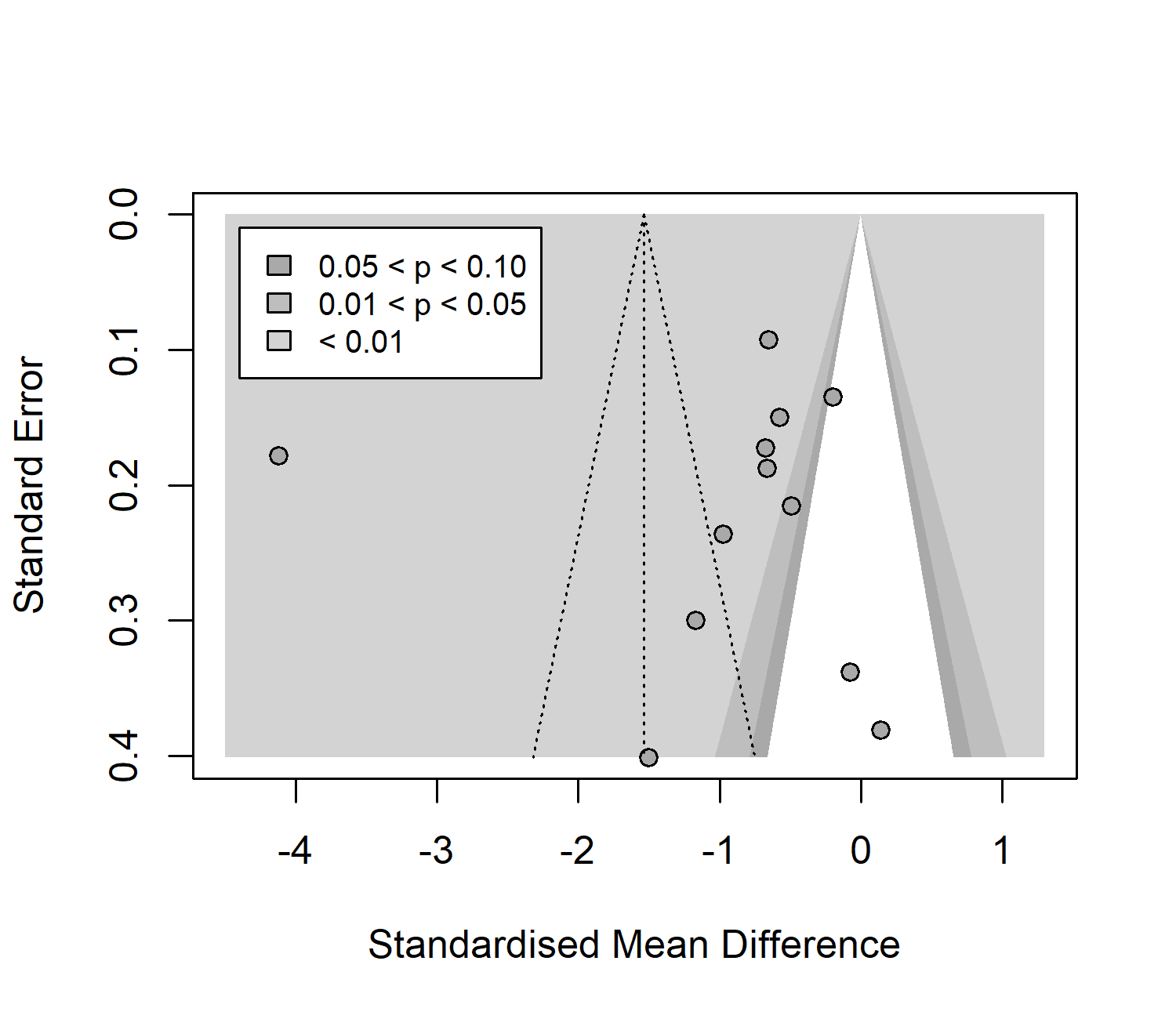


### Figure S48. The trim-and-fill funnel plot for HbA1c in type 2 diabetes studies


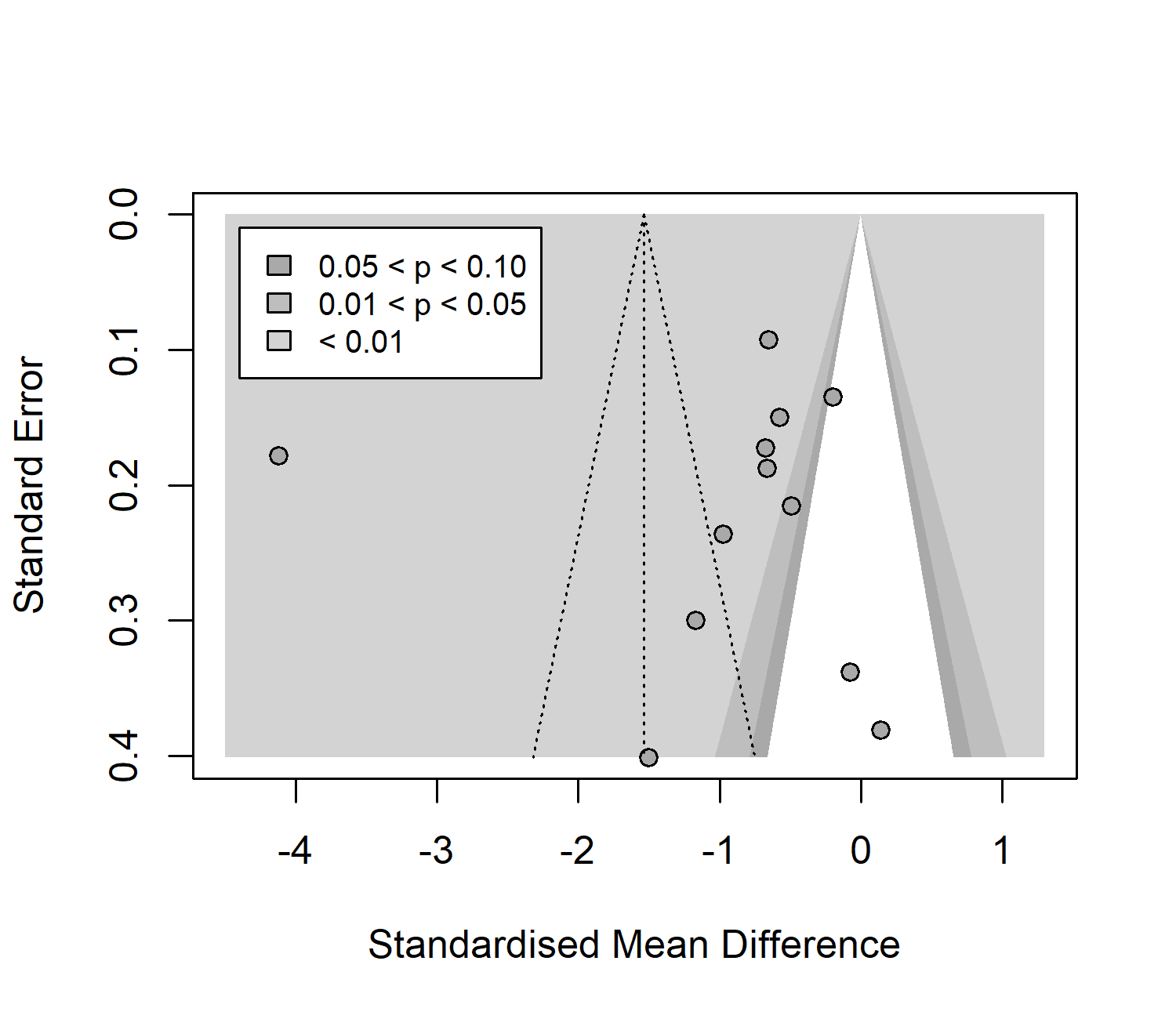


# Materials for Other Indicators in Three Diabetic Subtypes

## Table S12. Diabetes duration of patients in HNF1A-MODY studies

| Group | Disease | | No. of studies | No. of patients | Mean of diabetes duration (years) |
| --- | --- | --- | --- | --- | --- |
| All family members | | GCK-MODY | 6 | 662 | 10.78 |
|  |  | HNF1A-MODY | 6 | 441 | 17.24 |
| Probands | | GCK-MODY | 8 | 552 | 5.14 |
|  |  | HNF1A-MODY | 8 | 349 | 10.62 |

## Table S13. Diabetes duration of patients in type 2 diabetes studies

| Group | Disease | | No. of studies | No. of patients | Mean of diabetes duration (years) |
| --- | --- | --- | --- | --- | --- |
| All family members | | GCK-MODY | 8 | 772 | 11.80 |
|  |  | Type 2 Diabetes | 8 | 1337 | 8.07 |
| Probands | | GCK-MODY | 5 | 488 | 3.31 |
|  |  | Type 2 Diabetes | 5 | 1432 | 1.99 |

## Table S14. Age at recruitment of patients in HNF1A-MODY studies

| Group | Disease | No. of studies | No. of patients | Mean of age at recruitment (years) |
| --- | --- | --- | --- | --- |
| All family members | GCK-MODY | 9 | 851 | 32.60 |
|  | HNF1A-MODY | 9 | 492 | 38.58 |
| Probands | GCK-MODY | 12 | 706 | 13.48 |
|  | HNF1A-MODY | 12 | 321 | 19.62 |

## Table S15. Age at recruitment of patients in type 2 diabetes studies

| Group | Disease | No. of studies | No. of patients | Mean of age at recruitment (years) |
| --- | --- | --- | --- | --- |
| All family members | GCK-MODY | 7 | 565 | 36.41 |
|  | Type 2 Diabetes | 7 | 1039 | 54.29 |
| Probands | GCK-MODY | 3 | 194 | 12.05 |
|  | Type 2 Diabetes | 3 | 1003 | 14.54 |

## Figure S49. The forest plot for age at recruitment in HNF1A-MODY studies


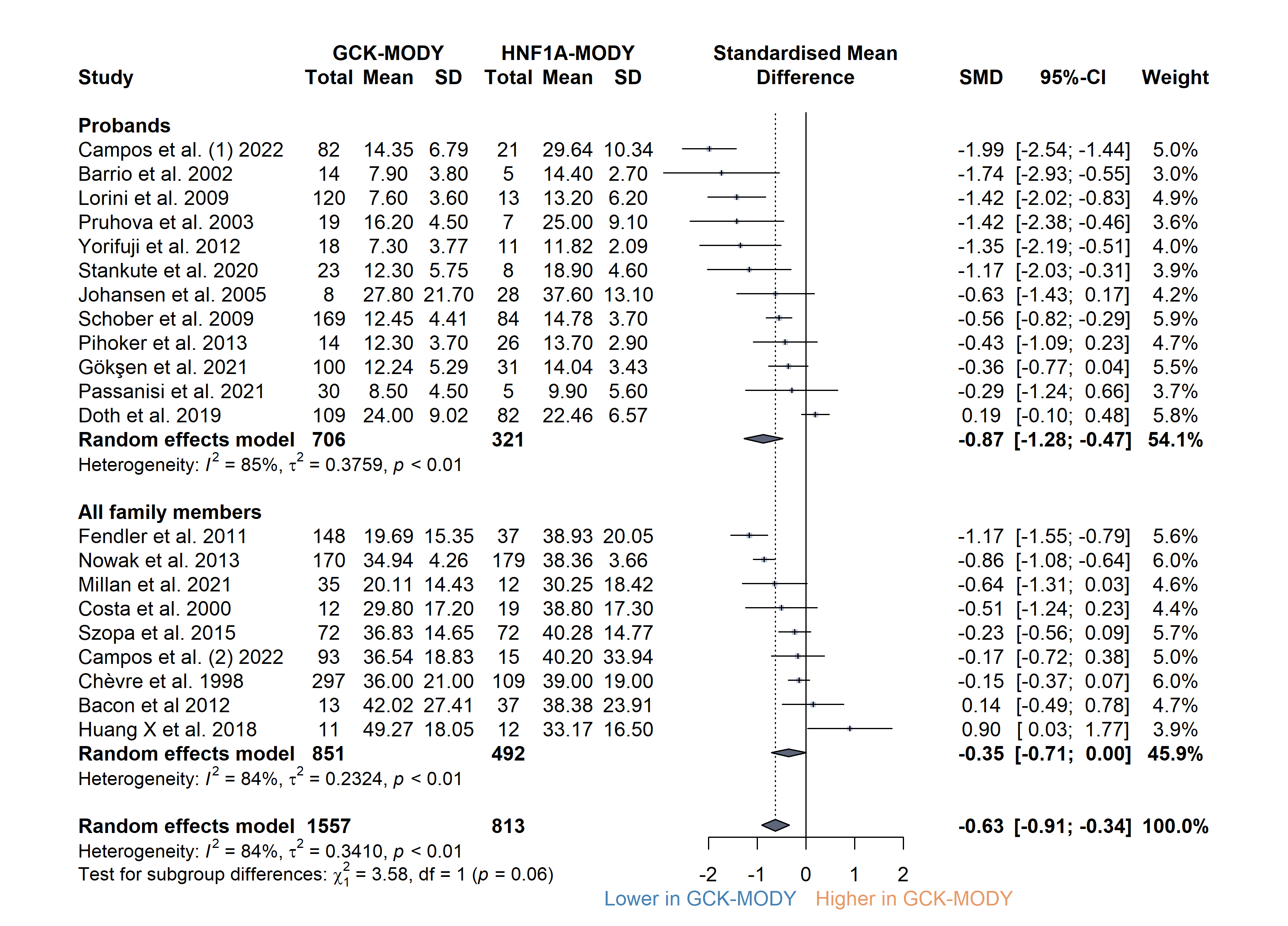


## Figure S50. The forest plot for age at recruitment in type 2 diabetes studies


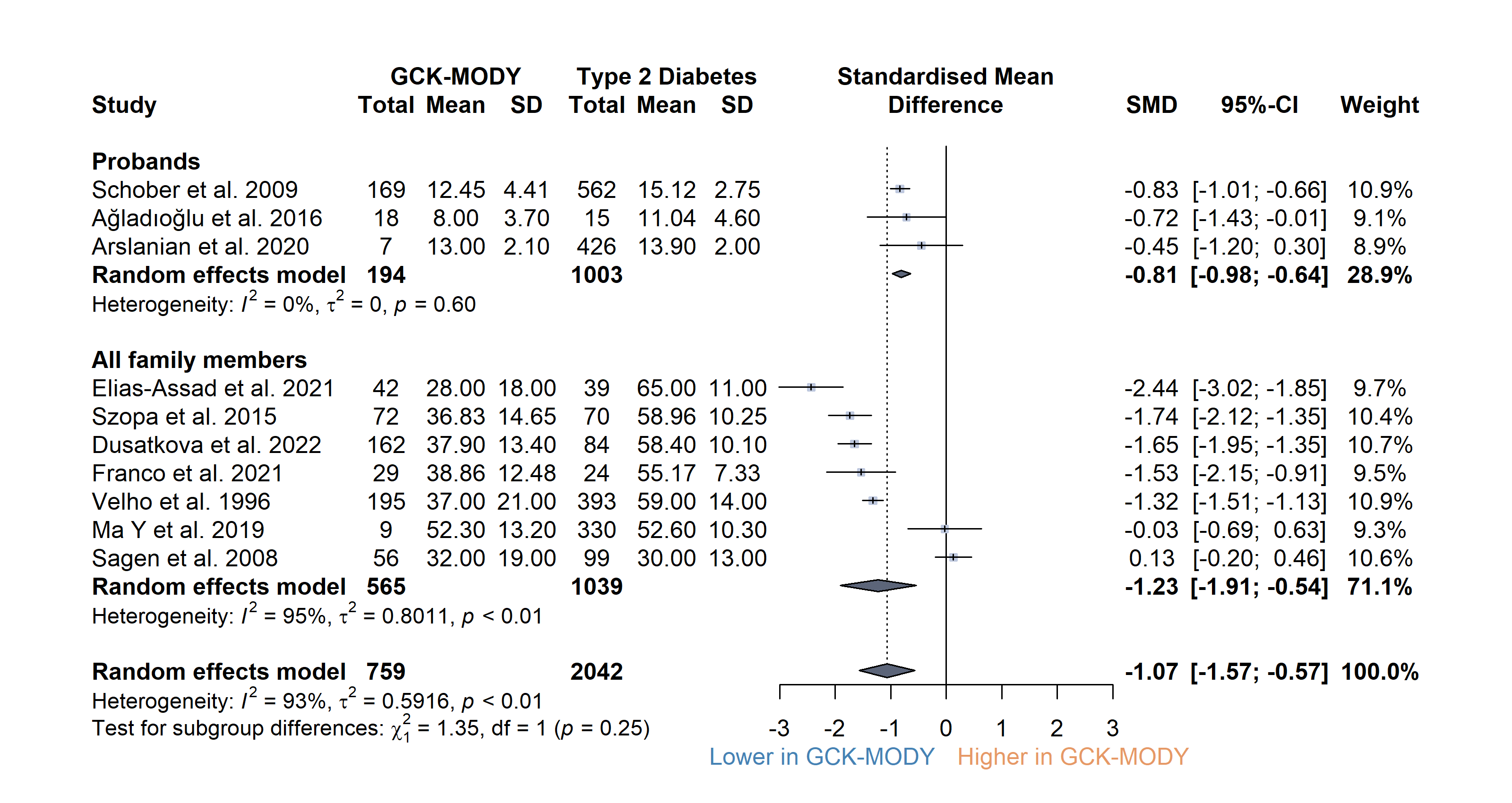


# Reference

1. Bacon S, Kyithar MP, Schmid J, et al. Serum levels of pancreatic stone protein (PSP)/reg1A as an indicator of beta-cell apoptosis suggest an increased apoptosis rate in hepatocyte nuclear factor 1 alpha (HNF1A-MODY) carriers from the third decade of life onward. Article. *BMC Endocr Disord*. Jul 18 2012;12:13. doi:10.1186/1472-6823-12-13

2. Chevre JC, Hani EH, Boutin P, et al. Mutation screening in 18 Caucasian families suggest the existence of other MODY genes. Research Support, Non-U.S. Gov't. *Diabetologia*. Sep 1998;41(9):1017-23. doi:10.1007/s001250051025

3. Costa A, Bescos M, Velho G, et al. Genetic and clinical characterisation of maturity-onset diabetes of the young in Spanish families. Research Support, Non-U.S. Gov't. *Eur J Endocrinol*. Apr 2000;142(4):380-6. doi:10.1530/eje.0.1420380

4. Fendler W, Borowiec M, Antosik K, et al. HDL cholesterol as a diagnostic tool for clinical differentiation of GCK-MODY from HNF1A-MODY and type 1 diabetes in children and young adults. *Clin Endocrinol (Oxf)*. Sep 2011;75(3):321-7. doi:10.1111/j.1365-2265.2011.04052.x

5. Owen KR, Thanabalasingham G, James TJ, et al. Assessment of high-sensitivity C-reactive protein levels as diagnostic discriminator of maturity-onset diabetes of the young due to HNF1A mutations. *Diabetes Care*. Sep 2010;33(9):1919-24. doi:10.2337/dc10-0288

6. Nowak N, Szopa M, Thanabalasingham G, et al. Cystatin C is not a good candidate biomarker for HNF1A-MODY. Article. *Acta Diabetol*. Oct 2013;50(5):815-20. doi:10.1007/s00592-012-0378-1

7. Millan AL, Trobo SI, de Dios A, et al. MODY patients exhibit shorter telomere length than non-diabetic subjects. Research Support, Non-U.S. Gov't. *Diabetes Metab Res Rev*. Feb 2021;37(2):e3374. doi:10.1002/dmrr.3374

8. Szopa M, Kapusta M, Matejko B, et al. Comparison of Glomerular Filtration Rate Estimation from Serum Creatinine and Cystatin C in HNF1A-MODY and Other Types of Diabetes. Comparative Study

Research Support, Non-U.S. Gov't. *J Diabetes Res*. 2015;2015:183094. doi:10.1155/2015/183094

9. Huang X, Gong S, Ma Y, et al. Lower Circulating miR-122 Level in Patients with HNF1A Variant-Induced Diabetes Compared with Type 2 Diabetes. Article. *Journal of Diabetes Research*. 2018;2018doi:10.1155/2018/7842064

10. Johansen A, Ek J, Mortensen HB, Pedersen O, Hansen T. Half of clinically defined maturity-onset diabetes of the young patients in Denmark do not have mutations in HNF4A, GCK, and TCF1. Research Support, Non-U.S. Gov't. *J Clin Endocrinol Metab*. Aug 2005;90(8):4607-14. doi:10.1210/jc.2005-0196

11. Donath X, Saint-Martin C, Dubois-Laforgue D, et al. Next-generation sequencing identifies monogenic diabetes in 16% of patients with late adolescence/adult-onset diabetes selected on a clinical basis: a cross-sectional analysis. Research Support, Non-U.S. Gov't. *BMC Med*. Jul 11 2019;17(1):132. doi:10.1186/s12916-019-1363-0

12. Campos Franco P, Santos de Santana L, Dantas Costa-Riquetto A, Santomauro Junior AC, Jorge AAL, Gurgel Teles M. Clinical and genetic characterization and long-term evaluation of individuals with maturity-onset diabetes of the young (MODY): The journey towards appropriate treatment. Article in Press. *Diabetes Res Clin Pract*. May 2022;187:109875. doi:10.1016/j.diabres.2022.109875

13. Pruhova S, Ek J, Lebl J, et al. Genetic epidemiology of MODY in the Czech republic: new mutations in the MODY genes HNF-4alpha, GCK and HNF-1alpha. Article. *Diabetologia*. Feb 2003;46(2):291-5. doi:10.1007/s00125-002-1010-7

14. Stankute I, Verkauskiene R, Blouin JL, et al. Systematic Genetic Study of Youth With Diabetes in a Single Country Reveals the Prevalence of Diabetes Subtypes, Novel Candidate Genes, and Response to Precision Therapy. Research Support, Non-U.S. Gov't. *Diabetes*. May 2020;69(5):1065-1071. doi:10.2337/db19-0974

15. Barrio R, Bellanne-Chantelot C, Moreno JC, et al. Nine novel mutations in maturity-onset diabetes of the young (MODY) candidate genes in 22 Spanish families. *J Clin Endocrinol Metab*. Jun 2002;87(6):2532-9. doi:10.1210/jcem.87.6.8530

16. Passanisi S, Salzano G, Bombaci B, Lombardo F. Clinical and genetic features of maturity-onset diabetes of the young in pediatric patients: a 12-year monocentric experience. Article. *Diabetol Metab Syndr*. Sep 8 2021;13(1):96. doi:10.1186/s13098-021-00716-6

17. Lorini R, Klersy C, d'Annunzio G, et al. Maturity-onset diabetes of the young in children with incidental hyperglycemia: a multicenter Italian study of 172 families. Multicenter Study. *Diabetes Care*. Oct 2009;32(10):1864-6. doi:10.2337/dc08-2018

18. Goksen D, Yesilkaya E, Ozen S, et al. Molecular Diagnosis of Monogenic Diabetes and Their Clinical/Laboratory Features in Turkish Children. Article. *J Clin Res Pediatr Endocrinol*. Nov 25 2021;13(4):433-438. doi:10.4274/jcrpe.galenos.2021.2021.0056

19. Pihoker C, Gilliam LK, Ellard S, et al. Prevalence, characteristics and clinical diagnosis of maturity onset diabetes of the young due to mutations in HNF1A, HNF4A, and glucokinase: results from the SEARCH for Diabetes in Youth. *J Clin Endocrinol Metab*. Oct 2013;98(10):4055-62. doi:10.1210/jc.2013-1279

20. Yorifuji T, Fujimaru R, Hosokawa Y, et al. Comprehensive molecular analysis of Japanese patients with pediatric-onset MODY-type diabetes mellitus. Research Support, Non-U.S. Gov't. *Pediatr Diabetes*. Feb 2012;13(1):26-32. doi:10.1111/j.1399-5448.2011.00827.x

21. Schober E, Rami B, Grabert M, et al. Phenotypical aspects of maturity-onset diabetes of the young (MODY diabetes) in comparison with Type 2 diabetes mellitus (T2DM) in children and adolescents: experience from a large multicentre database. Comparative Study

Multicenter Study

Research Support, Non-U.S. Gov't. *Diabet Med*. May 2009;26(5):466-73. doi:10.1111/j.1464-5491.2009.02720.x

22. Fu J, Ping F, Wang T, et al. A Clinical Prediction Model to Distinguish Maturity-Onset Diabetes of the Young From Type 1 and Type 2 Diabetes in the Chinese Population. *Endocr Pract*. Aug 2021;27(8):776-782. doi:10.1016/j.eprac.2021.05.002

23. Franco LF, Szarf G, Dotto RP, et al. Cardiovascular risk assessment by coronary artery calcium score in subjects with maturity-onset diabetes of the young caused by glucokinase mutations. *Diabetes Res Clin Pract*. Jun 2021;176:108867. doi:10.1016/j.diabres.2021.108867

24. Dusatkova P, Pavlikova M, Spirkova A, et al. Quality of Life and Treatment Satisfaction in Participants with Maturity-Onset Diabetes of the Young: A Comparison to Other Major Forms of Diabetes. Comparative Study. *Exp Clin Endocrinol Diabetes*. Feb 2022;130(2):85-93. doi:10.1055/a-1200-1482

25. McDonald TJ, Shields BM, Lawry J, et al. High-sensitivity CRP discriminates HNF1A-MODY from other subtypes of diabetes. *Diabetes Care*. Aug 2011;34(8):1860-2. doi:10.2337/dc11-0323

26. Ma Y, Han X, Zhou X, et al. A new clinical screening strategy and prevalence estimation for glucokinase variant-induced diabetes in an adult Chinese population. *Genet Med*. Apr 2019;21(4):939-947. doi:10.1038/s41436-018-0282-3

27. Sagen JV, Bjorkhaug L, Molnes J, et al. Diagnostic screening of MODY2/GCK mutations in the Norwegian MODY Registry. Research Support, Non-U.S. Gov't. *Pediatr Diabetes*. Oct 2008;9(5):442-9. doi:10.1111/j.1399-5448.2008.00399.x

28. Elias-Assad G, Saab R, Molnes J, et al. Maturity onset diabetes of the young type 2 (MODY2): Insight from an extended family. *Diabetes Res Clin Pract*. May 2021;175:108791. doi:10.1016/j.diabres.2021.108791

29. Velho G, Vaxillaire M, Boccio V, Charpentier G, Froguel P. Diabetes complications in NIDDM kindreds linked to the MODY3 locus on chromosome 12q. *Diabetes Care*. Sep 1996;19(9):915-9. doi:10.2337/diacare.19.9.915

30. Arslanian S, El Ghormli L, Haymond MH, et al. Beta cell function and insulin sensitivity in obese youth with maturity onset diabetes of youth mutations vs type 2 diabetes in TODAY: Longitudinal observations and glycemic failure. Randomized Controlled Trial

Research Support, N.I.H., Extramural. *Pediatr Diabetes*. Jun 2020;21(4):575-585. doi:10.1111/pedi.12998

31. Pacaud D, Schwandt A, de Beaufort C, et al. A description of clinician reported diagnosis of type 2 diabetes and other non-type 1 diabetes included in a large international multicentered pediatric diabetes registry (SWEET). Multicenter Study

Observational Study

Research Support, Non-U.S. Gov't. *Pediatr Diabetes*. Oct 2016;17 Suppl 23:24-31. doi:10.1111/pedi.12426

32. Agladioglu SY, Aycan Z, Cetinkaya S, et al. Maturity onset diabetes of youth (MODY) in Turkish children: sequence analysis of 11 causative genes by next generation sequencing. *J Pediatr Endocrinol Metab*. Apr 2016;29(4):487-96. doi:10.1515/jpem-2015-0039

33. Richards S, Aziz N, Bale S, et al. Standards and guidelines for the interpretation of sequence variants: a joint consensus recommendation of the American College of Medical Genetics and Genomics and the Association for Molecular Pathology. *Genet Med*. May 2015;17(5):405-24. doi:10.1038/gim.2015.30

34. American Diabetes Association Professional Practice C. 2. Classification and Diagnosis of Diabetes: Standards of Medical Care in Diabetes-2022. *Diabetes Care*. Jan 1 2022;45(Suppl 1):S17-S38. doi:10.2337/dc22-S002

35. Skupien J, Gorczynska-Kosiorz S, Klupa T, et al. Molecular background and clinical characteristics of HNF1A MODY in a Polish population. Research Support, Non-U.S. Gov't. *Diabetes & Metabolism*. Nov 2008;34(5):524-8. doi:10.1016/j.diabet.2008.05.004

36. Verkauskiene R, Danyte E, Dobrovolskiene R, et al. The course of diabetes in children, adolescents and young adults: does the autoimmunity status matter? *BMC Endocr Disord*. Nov 15 2016;16(1):61. doi:10.1186/s12902-016-0145-3

37. Lorini R, Alibrandi A, Vitali L, et al. Risk of type 1 diabetes development in children with incidental hyperglycemia: A multicenter Italian study. *Diabetes Care*. Jul 2001;24(7):1210-6. doi:10.2337/diacare.24.7.1210

38. Kyithar MP, Bacon S, Pannu KK, et al. Identification of HNF1A-MODY and HNF4A-MODY in Irish families: phenotypic characteristics and therapeutic implications. Research Support, Non-U.S. Gov't. *Diabetes Metab*. Dec 2011;37(6):512-9. doi:10.1016/j.diabet.2011.04.002

39. Liu W, Han X, Zhou X, et al. Brain derived neurotrophic factor in newly diagnosed diabetes and prediabetes. *Mol Cell Endocrinol*. Jul 5 2016;429:106-13. doi:10.1016/j.mce.2016.04.002

40. Arslanian S, El Ghormli L, Bacha F, et al. Adiponectin, Insulin Sensitivity, beta-Cell Function, and Racial/Ethnic Disparity in Treatment Failure Rates in TODAY. *Diabetes Care*. Jan 2017;40(1):85-93. doi:10.2337/dc16-0455

41. Giuffrida FMA, Moises RS, Weinert LS, et al. Maturity-onset diabetes of the young (MODY) in Brazil: Establishment of a national registry and appraisal of available genetic and clinical data. *Diabetes Res Clin Pract*. Jan 2017;123:134-142. doi:10.1016/j.diabres.2016.10.017

42. Froguel P, Velho G, Cohen D, Passa P. Strategies for the collection of sibling-pair data for genetic studies in type 2 (non-insulin-dependent) diabetes mellitus. *Diabetologia*. Sep 1991;34(9):685. doi:10.1007/BF00401001

43. Pruhova S, Dusatkova P, Sumnik Z, et al. Glucokinase diabetes in 103 families from a country-based study in the Czech Republic: geographically restricted distribution of two prevalent GCK mutations. Research Support, Non-U.S. Gov't. *Pediatr Diabetes*. Dec 2010;11(8):529-35. doi:10.1111/j.1399-5448.2010.00646.x
